# Genetic Diversity and Sequence Conservation of Peptide-Binding Regions of MHC Class I Genes in Pig, Cattle, Chimpanzee, and Human

**DOI:** 10.3390/genes15010007

**Published:** 2023-12-20

**Authors:** Seungyeon Youk, Mingue Kang, Byeongyong Ahn, Yangmo Koo, Chankyu Park

**Affiliations:** 1 Department of Stem Cell and Regenerative Biotechnology, Konkuk University, Gwangjin-gu, Seoul 05029, Republic of Korea; dbrtmddus@daum.net (S.Y.); mingue5349@gmail.com (M.K.); ahn.b@outlook.com (B.A.); 2 Genetic & Breeding Department, Korea Animal Improvement Association, Seocho, Seoul 06668, Republic of Korea; greatman009@daum.net

**Keywords:** MHC, peptide-binding region, genetic diversity, divergence time

## Abstract

Comparative analyses of MHC gene diversity and evolution across different species could offer valuable insights into the evolution of MHC genes. Intra- and inter-species sequence diversity and conservation of 12 classical major histocompatibility complex (MHC) class I genes from cattle, chimpanzees, pigs, and humans was analyzed using 20 representative allelic groups for each gene. The combined analysis of paralogous loci for each species revealed that intra-locus amino-acid sequence variations in the peptide-binding region (PBR) of MHC I genes did not differ significantly between species, ranging from 8.44% for *SLA* to 10.75% for BoLA class I genes. In contrast, intraspecies differences in the non-PBRs of these paralogous genes were more pronounced, varying from 4.59% for *SLA* to 16.89% for *HLA*. Interestingly, the Shannon diversity index and rate of nonsynonymous substitutions for PBR were significantly higher in *SLA* and BoLA than those in *Patr* and *HLA*. Analysis of peptide-binding pockets across all analyzed MHC class I genes of the four species indicated that pockets A and E showed the lowest and highest diversity, respectively. The estimated divergence times suggest that primate and artiodactyl MHC class I genes diverged 60.41 Mya, and BoLA and *SLA* genes diverged 35.34 Mya. These results offer new insights into the conservation and diversity of MHC class I genes in various mammalian species.

## 1. Introduction

The major histocompatibility complex (MHC) encodes glycoproteins responsible for delivering peptide fragments derived from various antigens to the cell surface. These fragments are then presented to T-cells and serve as antigen-recognition receptors in the adaptive immune system [1,2,3]. MHC class I molecules are expressed on nearly all cells and primarily induce immune responses in cytotoxic T-cells (CD8+ T-cells) [4,5]. Conversely, MHC class II molecules are predominantly expressed on antigen-presenting cells (APCs), such as dendritic cells or B-cells, and stimulate immune responses from CD4+ T-cells in response to peptides derived from extracellular pathogens, facilitating antibody production and local hypersensitivity reactions [4,5,6].

In mammals, classical MHC class I genes typically comprise eight exons, each encoding distinct functional domains. The presentation of peptides is primarily facilitated by the peptide-binding region (PBR), consisting of α1 and α2 domains encoded by exons 2 and 3. Due tothese functional characteristics, the sequences of MHC classes I and II within the PBR exhibit extensive sequence variations. Specifically, the PBR of MHC class I molecules contains six distinct binding pockets labeled A through F, which anchor antigenic peptides [7,8,9,10].

The genetic diversity of MHC molecules plays a crucial role in the adaptive immune responses of vertebrates, with numerous studies exploring how MHC polymorphisms influence the resistance and susceptibility of animals to infectious and autoimmune diseases [11,12,13,14,15]. However, most studies have focused on analyzing the genetic diversity within single species, as there are limited numbers of species with sufficiently diverse allelic groups to enable a comparative analysis of MHC gene diversity [16,17,18].

The evolutionary dynamics of MHC genes adhere to the birth and death model of genes, in which new genes emerge through repeated gene duplications [19,20]. Some of these duplicated genes remain stable over long evolutionary periods, while others are either deleted or become pseudogenes [21]. In addition, balancing selection is considered to play a key role in maintaining MHC gene diversity [22]. However, positive selection strongly influences genetic variation in the epitope-binding region [23]. Previous studies have shown that nonsynonymous substitutions are more frequent in the PBR of MHC than in the non-PBR in humans, mice, birds, cattle, and pigs [24,25,26,27,28]. However, the degree of differences or similarities between different species remains to be investigated.

The human MHC, also known as the human leukocyte antigen (*HLA*) [29], is considered to have evolved through replication, followed by diversification, coevolution, and sequence exchange. *HLA-A*, *HLA-B*, and *HLA-C* are the major classical MHC class I genes, with 7712, 9164, and 7672 alleles, respectively (Immuno Polymorphism Database IPD, “https://www.ebi.ac.uk/ipd/imgt/hla” (accessed on 5 October 2023). This indicates the extreme genetic diversity of MHC genes [30]. Chimpanzees (Pan troglodytes), the closest genetic relative to humans, show approximately 99% genomic similarity to humans. However, MHC class I genes consisting of *Patr-A*, *Patr-B*, and *Patr-C* exhibit only 86% sequence identity to class I genes in humans [31,32,33]. Progress in understanding the genetic diversity of MHC systems of cattle and pigs, known as the bovine leukocyte antigen (BoLA) and swine leukocyte antigen (*SLA*), respectively, have also been made using comprehensive typing methods [18,30,34,35,36,37,38,39].

MHC-A (homologs of *HLA-A*) and MHC-B (homologs of *HLA-B*) in primates have been present for at least 30 million years, as indicated by their presence in New and Old World primates [40,41,42]. However, the MHC-C locus (homolog of *HLA-C*), which exhibits sequence similarity to the MHC-B locus, is only found in gorillas, chimpanzees, and humans [43,44]. MHC-C locus is considered to have arisen due to replication of the MHC-B locus in the lineage leading to Old World primates and after a split between Old World primates and Hominidaea approximately 22.3 million years ago [45,46]. However, the divergence time of the MHC class I genes in pigs and cattle has not been investigated.

Here, we performed comparative inter-species analyses of the genetic diversity and conservation of MHC class I genes in pigs, cattle, humans, and chimpanzees. This analysis was based on many reported allelic sequences to IPD for each species. Our results broaden our understanding of species-specific differences and inter-species similarities in the genetic diversity and antigen-recognition capabilities of MHC class I genes in mammals.

## 2. Materials and Methods

### 2.1. Acquisition of DNA Sequences

A total of 25,735 nucleotide sequences of three classical MHC class I genes (*SLA-1*, *SLA-2*, *SLA-3*, *BoLA-1*, *BoLA-2*, *BoLA-3*, *Ovar-N*, *Patr-A*, *Patr-B*, *Patr-C*, *HLA-A*, *HLA-B, HLA-C*) of pigs (*Sus scrofa*, NCBI Taxonomy ID = 9823), cattle (*Bos taurus*, NCBI Taxonomy ID = 9913, sheep (*Ovis aries*, NCBI Taxonomy ID = 9940), chimpanzees (*Pan troglodytes*, NCBI Taxonomy ID = 9598), and humans (*Homo sapiens*, NCBI Taxonomy ID = 9606) were downloaded from the Immuno Polymorphism Database (IPD, “https://www.ebi.ac.uk/ipd/mhc” (accessed on 5 October 2023)) (Table S1). Sequences with loss-of-function characteristics, including premature stop codons, were excluded.

### 2.2. Sequence Alignment and Phylogenetic Analysis

In the MHC allele naming nomenclature, the first two digits of the 6-digit allele names following the locus name indicate the allelic group designated by the MHC nomenclature committee for each species [47,48,49,50]. Thus, alleles that are identical in the first 2-digits of allele names indicate high sequence similarity. Datasets for the peptide-binding region (PBR) and non-PBR regions were prepared separately. PBR corresponds to exons 2 (270 nucleotides, 89 amino acids) and 3 (276 nucleotides, 92 amino acids), and non-PBR corresponds to the remaining exons, including exons 1 and 4–8. For the PBR dataset, one sequence per allelic group with the lowest sequence similarity to the sequences of other allelic groups was selected, resulting in 20 sequences for loci with 20 or more 2-digit allelic groups (*SLA-1*, *BoLA-2*, *BoLA-3*, *Patr-A*, *Patr-B*, *HLA-A*, *HLA-B*, and *Ovar-N*). However, for loci with fewer than 20 allelic groups (*HLA-C*, *Patr-C*, *BoLA-1*, and *SLA-3*), additional sequences with the lowest similarity to the selected sequences were selected from the redundant allelic groups. Most *BoLA* sequences were from *Bos taurus*, except for three sequences from *Bos indicus* (Table S2). Finally, 258 sequences were prepared to be included in the PBR sequence dataset, yielding an equal number of alleles (*n* = 20) (Table S2). For the non-PBR dataset, 120 sequences consisted of 10 allelic sequences for each gene, as the number of alleles containing the full-length sequence information of all eight exons was limited, except for *HLA*. All sequence alignments were performed using the DNA alignment tool CLC Main Workbench 3 (CLC Bio, Aarhus, Denmark). Phylogenetic analysis was conducted using the neighbor-joining method implemented in MEGA11: Molecular Evolutionary Genetic Analysis, version 11 [51]. A total of 1000 bootstrap replicates were used to estimate support for the nodes in the obtained tree. The dataset for the phylogenetic analysis constitutes with a single representative sequence for each allelic group deduced from the alignment all alleles for all available allelic groups of each gene including *SLA-1* (*n* = 24), *SLA-2* (*n* = 22), *SLA-3* (*n* = 7), *BoLA-1* (*n* = 16), *BoLA-2* (*n* = 32), *BoLA-3* (*n* = 27), *Patr-A* (*n* = 24), *Patr-B* (*n* = 37), *Patr-C* (*n* = 15), *HLA-A* (*n* = 21), *HLA-B* (*n* = 36), and *HLA-C* (*n* = 14) (Table S2).

### 2.3. Determination of Consensus Amino-Acid Sequences and Genetic Distance Analysis

Consensus amino-acid sequences for each MHC gene were determined by sequence alignment using the CLC Main Workbench 3 (CLC Bio, Aarhus, Denmark). Sequence differences and similarities in the allelic sequences were determined by aligning them to the consensus amino-acid sequence of each MHC gene. Pairwise sequence differences were estimated by counting the number of nucleotide and amino-acid differences between the two sequences. Genetic distances between different loci were computed using the between-group mean distance option in MEGA11 [51] and expressed as percentages relative to the total sequence length.

### 2.4. Computation of Amino-Acid Conservation, Diversity, and Nucleotide Substitution Rate

Amino-acid frequencies at each amino-acid position and amino-acid conservation rates were estimated from the input alignment file using the Biopython v.1.81 SeqIO and AlignIO packages [52]. The amino-acid diversity for each MHC class I locus was estimated using the Shannon diversity index and Protein Variability Server (PVS) program [53]. The number of nonsynonymous substitutions for nonsynonymous sites (Ka), synonymous substitutions for synonymous sites (Ks), and Ka/Ks ratios for each MHC gene were calculated using DNAsp v6.12.0 [54]. The average Ka/Ks ratio was calculated by averaging the Ka/Ks ratios at all sites for each allele.

### 2.5. Estimation of Gene Divergence Times

The divergence times of MHC class I genes were estimated with 10 amino-acid sequences of different allelic groups for each gene using RelTime-ML in MEGA11 [51]. Platypus Ornithorhynchus anatinus (NCBI Taxonomy ID: 9258) was used as the outgroup. Calibration times were set according to previous estimations [40,45,46], in which 30 million years ago [55] were used as the calibration times between MHC-A and MHC-B [55].

### 2.6. Statistical Analysis

The statistical significance of differences in the levels of sequence diversity of MHC class I genes between different species was tested by Student’s *t*-test, considering each MHC class I paralogous gene of each species as experimental repeats. Statistical significance for differences in the degree of sequence variation of classical MHC class I genes among different species was tested using one-way ANOVA, while paralogous genes of each species were considered to be experimental repeats. Differences in the sequence diversity of the peptide-binding pockets were tested for the Shannon diversity index of each gene using two-way ANOVA, while paralogous genes and binding pockets were tested as two different parameters. Subsequently, a post hoc analysis of the ANOVA results was conducted using Tukey’s HSD. All analyses were conducted using R [22].

## 3. Results

### 3.1. Intraspecies Variation of Nucleotide Diversity in the Peptide-Binding Region of Classical MHC Class I Genes

The IPD database, “https://www.ebi.ac.uk/ipd/mhc” (accessed on 5 October 2023) includes 21 *HLA-A*, 36 *HLA-B*, 14 *HLA-C*, 24 *SLA-1*, 22 *SLA-2*, 7 *SLA-3*, 24 *Patr-A*, 37 *Patr-B*, 15 *Patr-C*, 15 *BoLA-1*, 32 *BoLA-2*, and 27 *BoLA-3* allelic groups based on the 2-digit MHC nomenclature system (Table S2). Then, 20 representative sequences from each of these genes were analyzed to understand the intraspecies species variations in the genetic diversity of the peptide-binding region (PBR), except *BoLA-1*, for which only 15 allelic groups of 18 alleles were available. The analysis included (Table S1) a total of 238 sequences with alength of 546 bp (181 amino acids) (Table S2, Figure S1).

**Figure 1:**
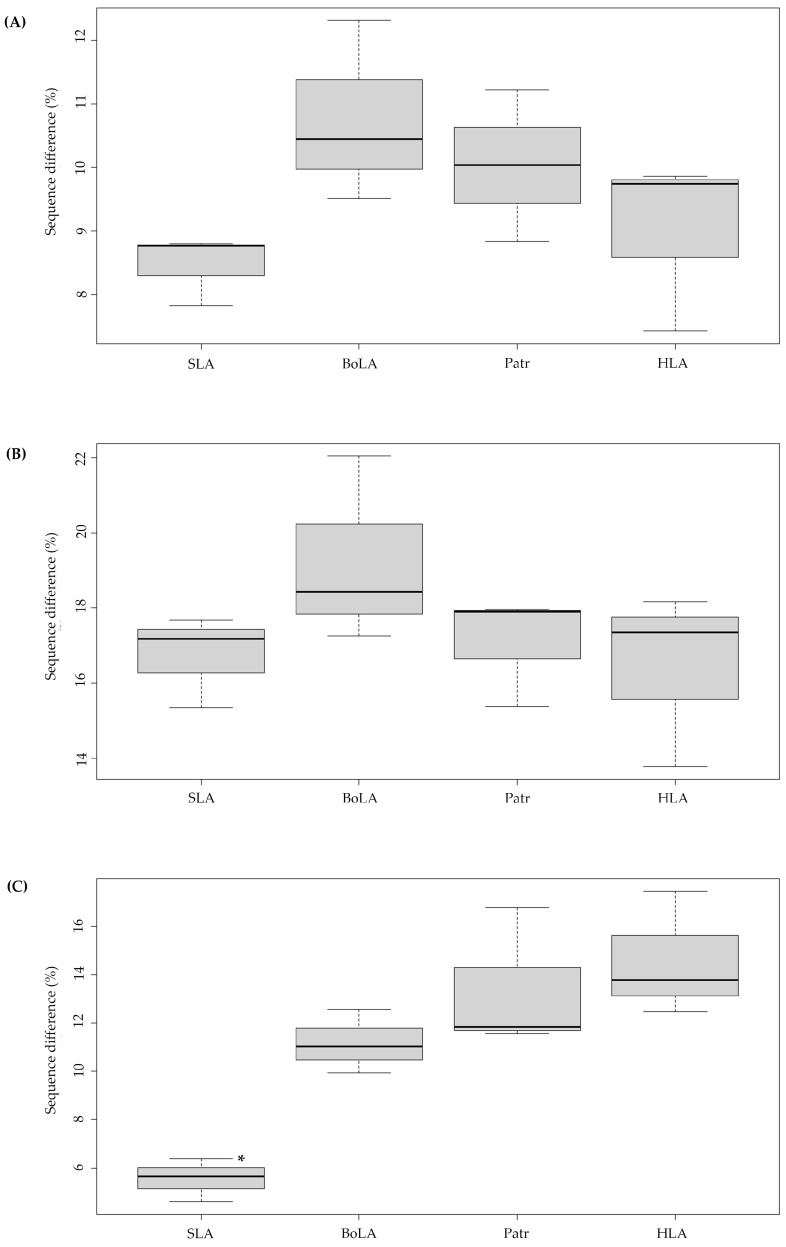
Boxplots showing the degree of sequence variations in the peptide-binding region and non-peptide-binding region of classical MHC class I paralogous genes in pigs, cattle, chimpanzees, and humans. *SLA*, Swine leukocyte antigen; *BoLA*, Bovine leukocyte antigen; *Patr*, Pan troglodytes leukocyte antigen; *HLA*, Human leukocyte antigen. *SLA* indicates the results of pairwise comparisons of selected *SLA-1*, *-2*, *-3* alleles; *BoLA* indicates the results of *BoLA-1*, *-2*, and *-3*; *Patr* indicates the results of *Patr-A*, *-B*, and *-C*; *HLA* indicates the results of *HLA-A*, *-B*, and *-C*. Y-axis indicates the difference in pairwise comparisons of the nucleotide (**A**) and amino-acid sequences (**B**) of the peptide-binding region between the alleles (*n* = 20) of each paralogous MHC gene (*n* = 3). (**C**) Pairwise amino-acid sequence differences in the nonpeptide-binding region between the alleles (*n* = 10) of each paralogous MHC gene (*n* = 3). “*” indicates statistical significance (*p* < 0.05) of the inter-species difference for the level of sequence variation.

The number of variable nucleotide sites identified across the PBR of the three MHC class I genes for each species was 7.82–8.79% for *SLA*, 9.51–12.31% for *BoLA*, 8.83–11.22% for *Patr*, and 7.42–9.86% for *HLA*, showing variations among class I paralogous genes within each species. The number of variable sites was lowest for *SLA* and highest for *BoLA*, yet the difference was not statistically significant (*p* = 0.162) (Figure 1A). The inter-species nucleotide sequence difference of PBR across the genes of the four different species was the lowest between *HLA* and *Patr*, as expected, while the differences were 5.10%, 6.65%, and 3.85% for *HLA-A* and *Patr-A*, *HLA-B* and *Patr-B*, and *HLA-C* and *Patr-C*, respectively (Table 1). The largest difference (average 16.56%) was observed between *BoLA-1* and *Patr-A*, in line with their phylogenetic distances. A significant difference was also found between *BoLA* and *SLA* (average 14.85%) despite both the cattle and pigs containing artiodactyls.

### 3.2. High Amino-Acid Sequence Diversity in the PBR of Pigs and Cattle MHC Class I Genes

We next analyzed the levels of amino-acid sequence variation in the PBR and non-PBRs of the *SLA*, *BoLA*, *Patr*, and *HLA* class I genes. The average amino-acid sequence differences between the PBR of the three paralogous MHC class I genes of each species, calculated by pairwise comparisons of alleles of paralogous genes, ranged from 15.3% to 17.68% for *SLA*, 17.25% to 22.04% for *BoLA*, 15.38% to 17.96% for *Patr*, and 13.77% to 18.16% for *HLA* (Figure 1B), indicating smaller differences at the amino-acid level than at the nucleotide sequence level (Table 1 and Table S3). In addition, sequence differences between paralogous non-PBRs for each species ranged from 4.59% to 6.27% for *SLA*, 10.11% to 12.64% for *BoLA*, 11.25% to 16.31% for *Patr*, and 12.06% to 16.89% for *HLA* (Figure 1C), indicating a significantly higher level of intraspecies diversity than that of PBR, likely due to differences in species divergence times.

We calculated the Shannon diversity index (H) for PBR and non-PBRs for each MHC class I gene (Table 2). The average H values of the three paralogous PBRs for *SLA*, *BoLA*, *Patr*, and *HLA* class I genes were 0.34, 0.33, 0.19, and 0.20, respectively. In contrast, the H values for the paralogous non-PBR *SLA*, *BoLA*, *Patr*, and *HLA* were 0.07, 0.10, 0.06, and 0.08, respectively, indicating a higher level of sequence conservation of non-PBR compared to PBR for all four species. In addition, the H values of the α1 and α2 domains constituting the PBR were similar in all analyzed genes except for *Patr-B* (Hα1 = 0.37, Hα2 = 0.2, *p*-value = 0.04), indicating that sequence variations are observed in the entire sequence of PBR of MHC class I genes. Interestingly, the H value of PBR of *SLA* and *BoLA* genes was significantly higher with an average of 0.34 and 0.33, respectively, compared to the result for *Patr* (0.19) and *HLA* genes (0.20), indicating higher diversity of PBR in artiodactyls than hominid species. PBR diversity was significantly low in *Patr-A*, *Patr-C*, and *HLA-C*, with H values of 0.15, 0.14, and 0.15, respectively. This indicates that the number of residing polymorphic sites per allele was larger for *SLA* and *BoLA* class I genes than for *Patr* and *HLA*.

Since the *HLA-B* allelic group included the highest number of alleles (*n* = 36), we further analyzed the inter-species diversity of MHC class I genes with increasing numbers of participating allelic groups to evaluate a potential effect of the size of participating allelic groups on PBR diversity(Table S4). The obtained result was consistent with that obtained using 20 allelic groups, suggesting that inter-species differences observed in H values were not due to differences in the number of participating allelic groups or alleles.

### 3.3. Increased Nonsynonymous Substitutions in MHC Class I PBR in Artiodactyl Species than in Primates

Next, we determined nonsynonymous (Ka) and synonymous substitution (Ks) sites in the PBR sequences of 13 pig, cattle, sheep, chimpanzee, and human MHC class I genes (Table 3). The average number of nonsynonymous substitution sites in the PBR of *HLA* and *Patr* class I genes was 39.67 and 38.33, respectively. In contrast, 62 and 58 nonsynonymous substitution sites were detected for *SLA* and *BoLA*, respectively, indicating a significant increase in nonsynonymous mutations in artiodactyls compared to hominid species. Among the *HLA* and *Patr* class I genes, *Patr-B* showed the highest nonsynonymous substitution sites (58), similar to the values in artiodactyl species. *Patr-B* also showed a higher number of synonymous substitutions than other MHC class I genes in humans and chimpanzees. Ka/Ks ratios for the α1 and α2 domains of 12 MHC class I genes were higher than 1 for the PBR α2 domain of all five species (Table 3), indicating the occurrence of positive selection. However, the value for the PBR α1 domain of *HLA-C*, *Patr-A*, *Patr-C*, and *BoLA-1* was lower than 1. The numbers of shared nonsynonymous substitution sites across paralogous genes in each species for *SLA* and *BoLA* were 17 and 19, respectively, which was higher than those in other species (Table S5). Human and chimpanzee MHC class I genes were found to share only four and two intraspecies conserved nonsynonymous sites, respectively. *BoLA* class I genes and *Ovar-N*, which wereincluded to evaluate the similarity in the nucleotide substitution rates of MHC class I genes between cattle and sheep, shared 11 conserved nonsynonymous substitution sites. Only one nonsynonymous site (position 349 of the α2 domain) was shared across *SLA, BoLA*, and *Ovar-N*. Interestingly, no shared nonsynonymous substitution sites were observed between *Patr* and *HLA* class I genes when 20 sequences from different allelic groups were compared. This may be due to the smaller number of variable sites between *Patr* and *HLA* class I genes than among the artiodactyls.

### 3.4. Phylogenetics Analysis

**Figure 2:**
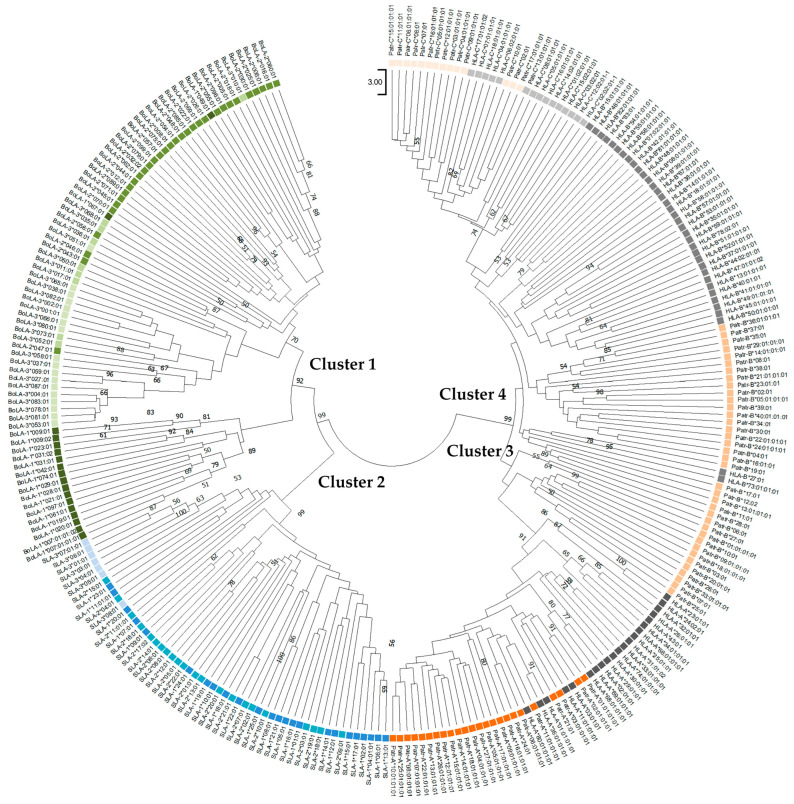
Phylogenetic tree of the peptide-binding region of classical MHC class I genes for pigs, cattle, chimpanzees, and humans. Twenty amino-acid sequences of different allelic groups were used for each gene. Boxes in different colors indicate different species; green, blue, orange, and gray represent cattle, pigs, chimpanzees, and humans, respectively. Therefore, paralogous genes of the same species are also depicted in the same color. Bootstrap values > 50 in 1000 repeats are shown above the nodes.

**Figure 3:**
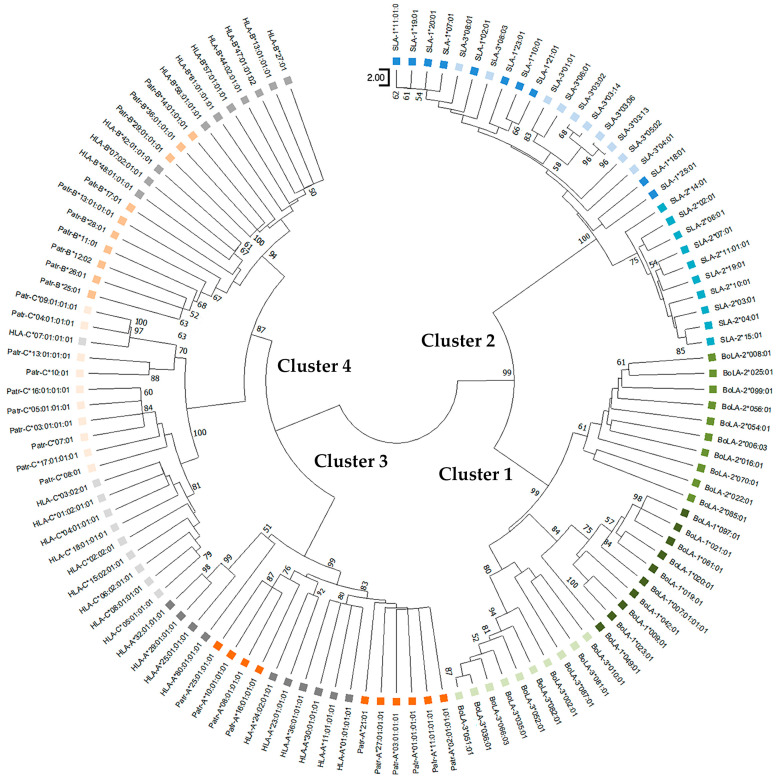
Phylogenetic relationships of the nonpeptide-binding region of classical MHC class I genes for pigs, cattle, chimpanzees, and humans. Ten amino-acid sequences of different allelic groups were used for each gene. Boxes in different colors indicate different species; green, blue, orange, and gray represent cattle, pigs, chimpanzees, and humans, respectively. Therefore, paralogous genes of the same species are also depicted in the same color. Bootstrap values > 50 in 1000 repeats are shown above the nodes.

Phylogenetic trees were constructed using the amino-acid sequences of orthologous PBRs (181 amino acids) and non-PBR (176–186 amino acids) sequences of the *SLA*, *BoLA*, *Patr*, and *HLA* class I genes (Figure 2 and Figure 3). The neighbor-joining tree constructed using PBR sequences yielded four main branches/clusters (Clusters 1–4) (Figure 2). Cluster 1 consisted of only *BoLA* alleles, although locus-specific sub-clustering of the three paralogous genes (*BoLA-1*, *BoLA-2*, and *BoLA-3*) was incomplete. For example, the *BoLA-1* and *-2*, including BoLA-2*03:01, BoLA-1*049:01, BoLA-1*067:01, BoLA-2*046*01, BoLA-2*043:01, and BoLA-2*047:01, was not found to cluster to specific loci together. Cluster 2 was composed only of *SLA* class I genes; however, the consistency of the locus-specific clustering of *SLA-1*, *-2*, and *-3* was lower than that of *BoLA* in Cluster 1. Cluster 3 consisted of the *Patr-A* and *HLA-A* alleles, together with several *Patr-B* alleles. Most of the *Patr-A* and *HLA-A* alleles formed two separate subclusters yet shared the same branch at a higher level, suggesting a close genetic relationship between the two genes. However, *Patr-B* formed separate subclusters from *Patr-A* and *HLA-A* within Cluster 3. Cluster 4 consisted of *HLA-B*, *Patr-B*, *HLA-C*, and *Patr-C* alleles. Although sequences belonging to the same locus mostly clustered together, several *HLA-B* and *HLA-C* alleles were intermixed with *Patr-B* and *Patr-C*, further supporting a common ancestry between *HLA-B* and *Patr-B* and between *HLA-C* and *Patr-C*.

The phylogenetic tree of the non-PBR also formed four main clusters (Clusters 1–4), similar to that of the PBR (Figure 3). Cluster 1 of the non-PBR tree comprised locus-specific subclusters of the *BoLA-1*, *BoLA-2*, and *BoLA-3* alleles. Cluster 2 exhibited locus-specific subclusters for *SLA-2*; however, the *SLA-1* and *SLA-3* alleles were intermixed and shared branches at a deeper level. Cluster 3 contained alleles of *Patr-A* and *HLA-A* loci. Cluster 4 comprised alleles of *Patr-B* and *HLA-B* as well as *Patr-C* and *HLA-C*. Tree of non-PBR sequences. Locus specificity was clearer than that of PBR, and paralogous genes formed subclusters more tightly in a locus-specific manner than in PBR.

### 3.5. Intraspecies Conservation of the Genetic Diversity Level for Peptide-Binding Pockets A and E

We determined the residues constituting the peptide-binding pockets (A to F) in the PBR of pig, cattle, and chimpanzee MHC class I sequences based on available *HLA* structures [7,8,56,57] (Table S6). We then calculated the H values of the pocket residues (Table 4). The mean H of *SLA-1*, *-2*, and *-3* for the six pockets ranged from 0.54 for pocket A to 1.55 for pocket E, showing significant differences across pockets (Table S7). The sequence diversity of peptide-binding pockets varied somewhat among species; however, the characteristic feature of A and E pockets, being the pockets of lowest and highest diversity, respectively, were well conserved across all 13 MHC class I molecules of five different species, including sheep. The diversity levels in pockets B, C, and F were variable, with larger variations in *SLA* and *BoLA* than in *Patr* and *HLA*, in line with our results of the inter-species comparison of PBR diversity.

### 3.6. Presence of Conserved Sites in the Epitope-Binding Region of MHC Class I Genes across Different Species

**Figure 4:**
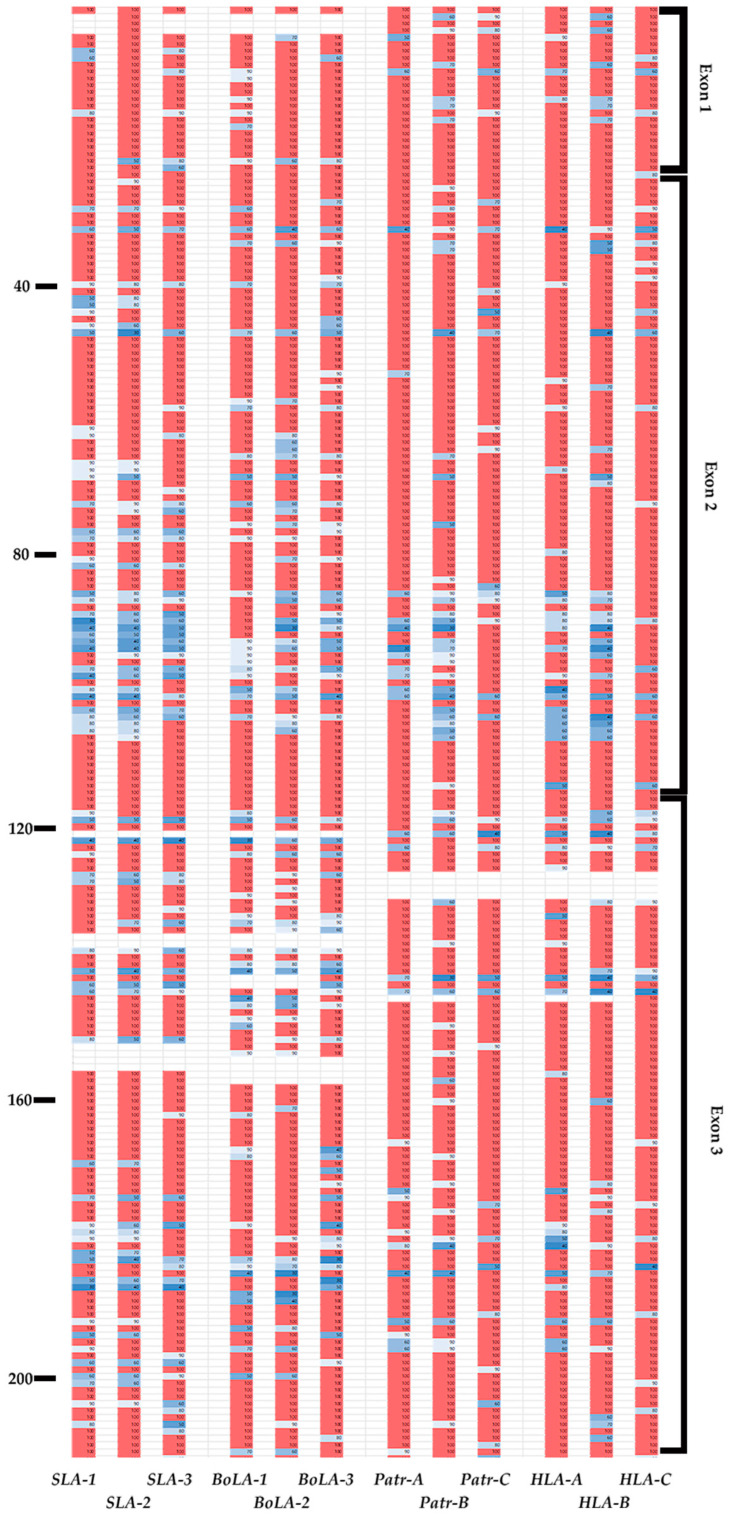

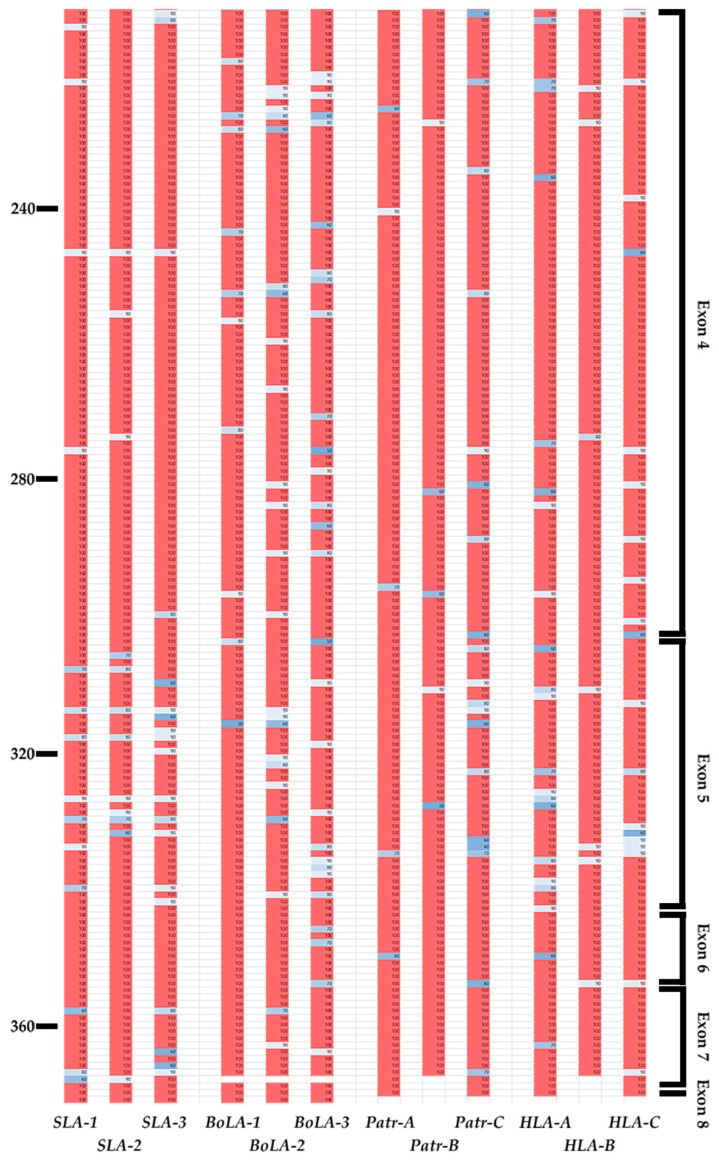
Sequence conservation patterns in the coding region of classical MHC class I genes of pigs, cattle, chimpanzees, and humans. The levels of amino-acid sequence conservation for each residue from exons 1 to 8 of 12 MHC class I genes are indicated in the column for each gene and shown with different colors; pink and white represent 100% and 90–95% conservation, respectively. Colors darken to blue (30 to 90%) as conservation decreases. Amino-acid positions with no values represent gaps in the multiple alignment. The position number of amino acids is indicated on the left, and corresponding exon names are indicated on the right. The gene names are indicated at the bottom.

**Figure 5:**
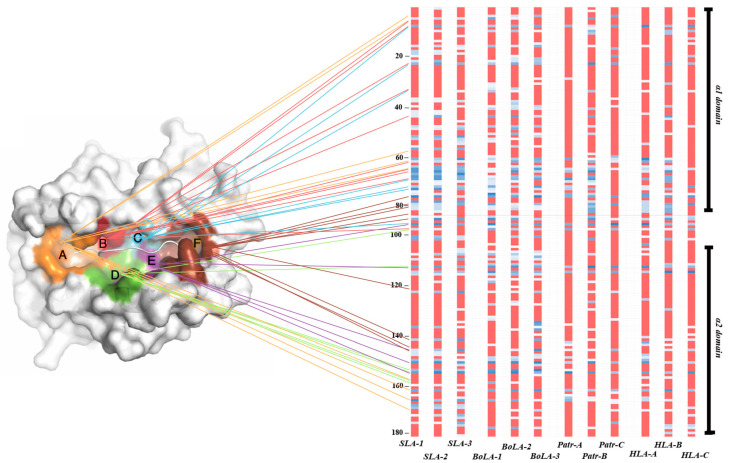
Sequence conservation patterns of the peptide-binding region of classical MHC class I genes relative to peptide-binding pockets in pigs, cattle, chimpanzees, and humans. The 3D structure of the MHC protein is illustrated on the left. Peptide-binding pockets A to F are color-coded (orange, A; red, B; blue, C; green, D; purple, E; brown, F) and adopted from Nguyen et al. [57]. Amino-acid positions corresponding to each pocket are marked with the same color. The sequence alignment on the right represents the level of sequence conservation. Pink and white represent 100% and 90–95% conservation, respectively. Colors darken to blue (50 to 95%) as conservation decreases. The positions of amino acids, gene names, and domain names are indicated on the left, bottom, and right of the alignment, respectively.

We analyzed inter-species conservation of the amino-acid sequences of MHC class I proteins across pigs, cattle, chimpanzees, and humans (Figure 4 and Figure 5). Most amino-acid variations were found to be located between positions 86 and 115 in the α1 domain in exon 2 and positions 170 and 200 in the α2 domain in exon 3 (Figure 4). In the PBR, we observed the complete conservation of amino acids across all four species at positions 24–36, 82–92, and 117–134, which constitutes 23.76% (42 positions) of exons 2 and 3 (Figure 5). Among these conserved positions, seven (A pocket: positions 6, 58, and 158; B pocket: position 6; D pocket: position 158; and F pocket: positions 83, 122, and 145) were involved in peptide-binding. Except for positions 6 and 158, the remaining positions were involved in the formation of the A or F pockets. Gln in position 114 was conserved across all four species, whereas Asp in position 121 was conserved except for *BoLA-2*, which included Asn in this position. Glu in position 127 was conserved except for *Patr-B*, which included Glu, Gln, and Lys, and *BoLA-2* Glu and Gln in this position. In *SLA*, positions 139, 146, and 152 showed complete conservation of Gln, Asp, and Glu across *SLA-1*, *-2*, and *-3*, respectively (Tables S8–S11). This was also observed in *BoLA-1*, *BoLA-3*, *Patr-A*, *Patr-C*, *HLA-A*, *HLA-B*, and *HLA-C*, except for *BoLA-2*, which included Ala and *Patr-B*, which included Ala and Pro in these positions.

### 3.7. Divergence Time of MHC Class I Genes between Artiodactyls and Primate Lineages

**Figure 6:**
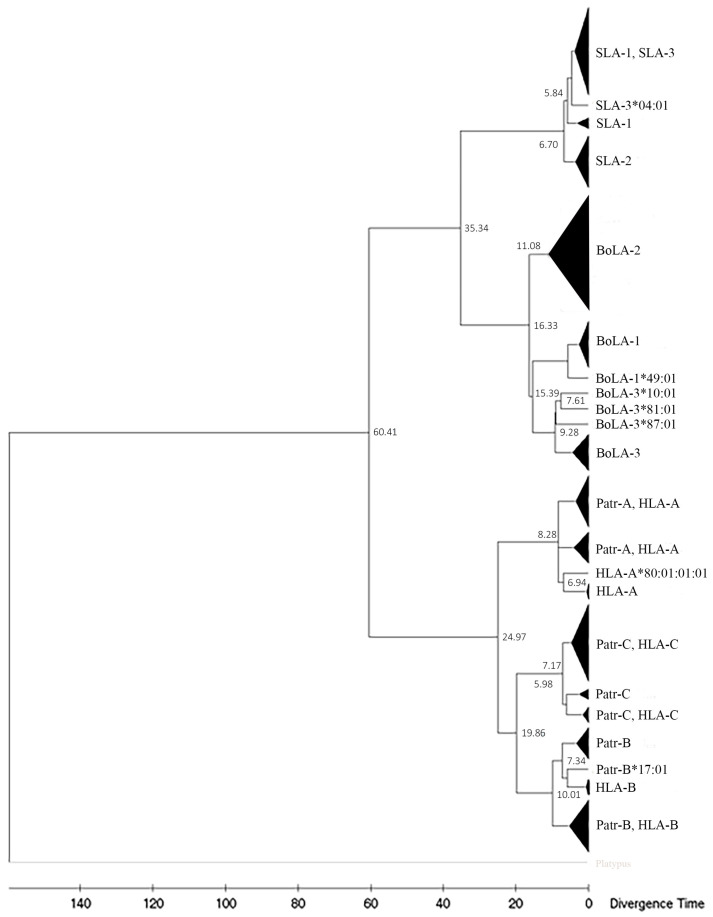
Estimation of divergence times for MHC class I genes of pigs, cattle, sheep, chimpanzees, and humans. A total of 131 amino-acid sequences of the nonpeptide-binding region of MHC class I genes consisting of 10 sequences each for different genes was used. Alleles in the same cluster are merged and represented by black triangles. The X-axis indicates time. The divergence times of each lineage are shown next to each node. Gene names are indicated at branch tips.

We estimated the divergence time of classical MHC class I genes in pigs, cattle, chimpanzees, sheep, and humans, along with platypus as an outgroup, using 131 non-PBR sequences consisting of 10 non-PBR sequences of different allelic groups for each gene (Figure 6). PBR was excluded to minimize the estimation bias from evolutionary forces on the MHC genes. The divergence between primate and artiodactyl MHC class I genes was estimated to be approximately 60.41 Mya. In the artiodactyl lineage, the separation of *BoLA* and *SLA* was estimated to be 35.34 Mya, and subsequently, *BoLA-2* was estimated to be separated from the ancestral gene of *BoLA-1* and *BoLA-3* around 16.33 mya. The divergence between *BoLA-1* and *BoLA-3* was estimated to be 15.39 Mya. In pigs, the divergence of *SLA-1*, *-2*, and *-3* occurred more recently around 6.70 Mya. In the primate lineage, the divergence of MHC-A (*HLA-A*, *Patr-A*, and related genes) from the common ancestral gene MHC-B (*HLA-B*, *Patr-B*, and related genes) and the MHC-C locus (*HLA-C*, *Patr-C*, and related genes) was estimated to be 24.97 Mya. The subsequent separation of MHC-B and MHC-C was estimated to be 19.86 Mya.

## 4. Discussion

Most studies on the evolution and diversity of MHC genes have centered on population studies within a single species under varying demographic or environmental conditions, highlighting the role of MHC genetic variation in resistance to pathogens and parasites and animal survival [16,17,18]. Previous studies have also suggested that MHC variability is driven by pathogen-driven selection, either through heterozygote advantage or frequency-dependent selection [58,59]. To this end, comparative analyses of MHC gene diversity and evolution across different species could offer valuable insights into the evolution of MHC genes. However, challenges in MHC allele typing due to extensive polymorphisms in MHC genes hamper the accumulation and interpretation of large-scale typing results from diverse species.

Here, we conducted a comparative analysis of genetic variations in three major MHC class I genes in pigs, cattle, chimpanzees, and humans. These species were chosen due to the relatively extensive amount of information available on their MHC genetic diversity. Despite unclear orthologous relationships among the paralogous MHC genes of these species, we compared sequence variations in the major classical MHC class I genes both within and across species. We confirmed that consistent with previous reports, the PBR of all analyzed MHC genes harbored much higher variability than non-PBRs across all analyzed species [60,61,62,63]. Additionally, our analysis revealed differences in genetic diversity among the MHC class I genes of different species, showing more substitution sites in classical MHC class I genes in pigs and cattle compared to humans and chimpanzees when an equal number of allelic groups from the IPD were analyzed. Our results may have been affected by a bias in assigning allelic groups of MHC class I genes to different species. However, because the number of allelic groups (*n* = 20) used in this study represents most of the allelic groups reported for the analyzed species, we reasoned that our results represent the evolutionary consequences of MHC class I genes in these species. Furthermore, the analysis results from the sequences of all allelic groups (*n* = 36) in *HLA-B* were consistent with those obtained using 20 allelic groups (Table S4), supporting the hypothesis that the rate of nonsynonymous substitution was higher in artiodactyls than in hominid species, raising questions regarding the underlying mechanism.

It is noteworthy that pigs and cattle exhibit a higher rate of nonsynonymous substitutions in the PBR of MHC class I genes than humans and chimpanzees despite the identical role of MHC in antigen recognition across mammals. Only several differences in the adaptive immune system between the two groups have been reported, including a larger number of γ delta T-cells in pigs and cattle than in humans [64,65,66], yet it remains unclear whether such differences could be related to the genetic diversity of MHC genes. Previous studies have indicated that the number of MHC alleles and their genetic diversity are not always correlated, as demonstrated by a high degree of divergence between MHC alleles in bottlenecked species such as Przewalski’s horse, Arabian oryx, and South African bontebok, despite their low allele numbers [67,68,69]. Certain promiscuous MHC molecules can bind to a much wider range of peptides or show an elevated peptide-binding repertoire than others and, therefore, promote immune responses against a broad range of pathogens. This is a complex aspect of the genetic diversity of MHC [70,71]. Manczinger et al. [72] demonstrated a direct relationship between MHC diversity and pathogens, revealing high levels of promiscuous *HLA* alleles in Southeast Asia, an important hotspot for emerging infectious diseases.

The diversity of MHC molecules should also be related to the diversity of pathogens that infect a species. Therefore, the increased number of substitution sites and rate of nonsynonymous substitution in the PBR of artiodactyl compared to hominid species may indicate the presence of larger pathogen diversity in the former than in the latter. Environmental conditions, such as habitats with a lower chance of pathogen exposure, might have influenced the reduction of antigenic repertoires and resulted in the lower genetic diversity of *HLA* and *Patr* compared to *SLA* and *BoLA*. However, no clear evidence supporting this hypothesis has been obtained to date.

Our phylogenetic analysis of *HLA* and *Patr* MHC I sequences revealed trans-species polymorphisms in MHC, which are often ancient and predate speciation events. Several class I MHC alleles from humans and chimpanzees belong to the allelic lineage that has persisted since before these two species diverged 5–7 Mya (Figure 6) [73,74,75]. However, none of the class I MHC alleles of *SLA* and *BoLA* formed shared clusters, consistent withtheir later divergence, 35.34 Mya, after the speciation of pigs and cattle 60 Mya [76].

Theoretically, under identical selection pressure, the level of genetic diversity of orthologous genes should be the same between two mammalian sublineages. Although accurate assessments of the evolutionary forces affecting the genetic diversity of MHC genes are best conducted in free-ranging animal populations in their natural environments, our results nevertheless imply that the number of variable sites in MHC class I genes is significantly higher in *SLA* and *BoLA* than in *Patr* and *HLA*. This may indicate differences in selective pressures, population structures, or genetic diversity between these two lineages.

Gene duplication, mutation, or other processes can generate new genes and alleles and, therefore, increase genetic variation [77,78]. New genetic variations can emerge within generations in a population, and populations with rapid reproductive rates are likely to exhibit high genetic variation. The shorter generation intervals in artiodactyl species compared to hominid species may contribute to the higher genetic variation observed in *SLA* and *BoLA* MHC class I genes.

Here, we observed the conservation of several polymorphic sites within the peptide-binding region of paralogous MHC class I proteins of the same species and among orthologous proteins of different species. These conserved sites are likely important for the function of MHC molecules. For example, the conserved amino-acid positions 114, 121, and 127 have been reported to be crucial for interactions between *HLA* class I molecules and CD8+ T-cells [79]. Understanding the functional roles of other conserved sites in the PBR across different species in this study could help delineate the interactions between MHC molecules and antigenic peptides as well.

In conclusion, we compared the genetic diversity of MHC class I paralogous and orthologous genes of pigs, cattle, humans, and chimpanzees using representative sequences of diverse allelic groups. We identified characteristics of MHC diversity between artiodactyl and hominid species. These findings stem from comparative analyses conducted on the largest collection of MHC alleles to date, encompassing numerous allelic groups across multiple species. Additionally, we identified potentially important amino-acid residues crucial for the function of MHC class I molecules in the adaptive immune system of mammals. The insights gained from this study enhance our understanding of the conservation and diversity of MHC class I genes across different mammalian species.

## Figures and Tables

**Table 1:** Inter-species pairwise amino-acid sequence differences for the peptide-binding and non-binding regions of the classical MHC class I genes of pigs, cattle, chimpanzees, and humans.

Sequence Differences (%) ^a^													
		*BoLA*			*HLA*			*Patr*			*SLA*		
		*1*	*2*	*3*	*A*	*B*	*C*	*A*	*B*	*C*	*1*	*2*	*3*
*BoLA-1*													
*BoLA-2*	non-PBR ^b^	9.94											
	PBR ^c^	18.43											
*BoLA-3*	non-PBR	12.57	11.02										
	PBR	22.04	17.25										
*HLA-A*	non-PBR	27.66	25.84	29.94									
	PBR	25.10	23.69	26.72									
*HLA-B*	non-PBR	24.86	23.04	26.28	13.79								
	PBR	22.40	23.06	25.46	18.16								
*HLA-C*	non-PBR	29.02	27.01	29.99	17.45	12.47							
	PBR	23.75	21.92	23.55	17.34	13.77							
*Patr-A*	non-PBR	26.20	24.39	28.62	4.26	12.83	16.86						
	PBR	24.69	23.16	26.29	9.84	19.09	17.41						
*Patr-B*	non-PBR	24.24	22.27	25.62	12.97	3.99	11.68	11.83					
	PBR	22.70	23.26	25.63	17.34	12.51	13.86	17.90					
*Patr-C*	non-PBR	29.26	27.21	30.18	17.27	12.22	5.32	16.77	11.56				
	PBR	24.66	22.98	24.60	18.57	15.47	7.93	17.96	15.38				
*SLA-1*	non-PBR	21.18	21.05	23.80	28.47	27.27	29.72	27.22	27.44	29.67			
	PBR	26.28	24.62	27.02	26.69	26.73	25.50	26.30	26.62	26.15			
*SLA-2*	non-PBR	22.46	21.64	24.14	29.29	27.28	30.17	28.00	27.44	30.13	5.66		
	PBR	25.87	24.61	26.83	26.90	26.12	25.29	26.56	26.12	26.07	15.34		
*SLA-3*	non-PBR	21.57	21.12	23.60	29.17	27.78	29.82	27.82	27.92	29.81	4.59	6.38	
	PBR	25.29	24.31	26.43	26.74	25.70	24.92	26.05	25.55	25.24	17.68	17.18	

^a^ The numbers indicate the mean nucleotide sequence differences (%) in the pairwise comparison of the analyzed alleles (*n* = 20 and 10 for PBR and non-PBR, respectively) for each gene. ^b^ Corresponding to exons 1, 4, and 8 of classical MHC class I genes. ^c^ Corresponds to the exons 2 and 3 of the classical MHC class I genes. Note. *SLA*, Swine leukocyte antigen; *BoLA*, Bovine leukocyte antigen; *Patr*, Pan troglodytes leukocyte antigen; *HLA*, Human leukocyte antigen.

**Table 2:** Amino-acid sequence diversities in the classical MHC class I genes of pigs, cattle, chimpanzees, and humans.

Shannon Diversity Index (H)													
Region		*BoLA*			*HLA*			*Patr*			*SLA*		
		*1* *	*2* *	*3* *	*A* *	*B* *	*C* *	*A* *	*B* *^,^**	*C*	*1* *	*2* *	*3* *
PBR ^a^	Entire region	0.30	0.35	0.33	0.21	0.25	0.15	0.15	0.28	0.14	0.36	0.37	0.30
	α1 domain	0.31	0.4	0.34	0.21	0.3	0.13	0.15	0.37	0.12	0.44	0.44	0.31
	α2 domain	0.33	0.34	0.35	0.23	0.22	0.18	0.16	0.21	0.16	0.33	0.34	0.3
Non-PBR ^b^		0.06	0.10	0.13	0.10	0.08	0.08	0.03	0.05	0.10	0.07	0.05	0.08

“*” and “**” following the locus name indicate the statistical significance of sequence diversity (*p* < 0.05) using the Shannon diversity index between PBR and non-PBR strains and between the a1 and a2 domains, respectively. The Student’s *t*-test was used. ^a^ Corresponds to exons 2 and 3 of classical MHC class I genes. ^b^ Corresponds to exons 1, 4, and 8 of classical MHC class I genes. Note. *SLA*, Swine leukocyte antigen; *BoLA*, Bovine leukocyte antigen; *Patr*, Pan troglodytes leukocyte antigen; *HLA*, Human leukocyte antigen.

**Table 3:** The level of genetic variation and substitution rates in the nucleotide sequence of the peptide-binding region for classical MHC class I genes of pigs, cattle, sheep, chimpanzees, and humans.

Species	Locus	No. of Variable Sites (%) ^a^	No. of Synonymous Sites (%) ^b^ (A)	No. of Nonsynonymous Sites (%) ^b^ (B)	(A)/(B) Ratio	Ka/Ks Ratio	
						α1 Domain	α2 Domain
Human	*HLA-A*	71 (13.00)	12 (2.20)	43 (7.88)	0.28	1.74	1.57
	*HLA-B*	83 (15.20)	17 (3.11)	45 (8.24)	0.38	1.40	1.20
	*HLA-C*	58 (10.62)	13 (2.38)	31 (5.68)	0.42	0.75	1.18
	Mean	70.67 (12.94)	14.00 (2.56)	39.67 (7.26)	0.36	1.30	1.32
Chimpanzee	*Patr-A*	54 (9.89)	16 (2.93)	27 (4.95)	0.59	0.89	1.12
	*Patr-B*	100 (18.32)	22 (4.03)	58 (10.62)	0.38	1.08	1.21
	*Patr-C*	52 (9.52)	10 (1.83)	30 (5.49)	0.33	0.70	1.71
	Mean	68.67 (12.58)	16.00 (2.93)	38.33 (7.02)	0.44	0.89	1.35
Cattle	*BoLA-1*	128 (23.44)	30 (5.49)	59 (10.81)	0.51	0.63	1.63
	*BoLA-2*	124 (22.71)	23 (4.21)	55 (10.07)	0.42	1.05	1.65
	*BoLA-3*	119 (21.79)	26 (4.76)	60 (10.99)	0.43	1.80	1.18
	Mean	123.67 (22.65)	26.33 (4.82)	58.00 (10.62)	0.45	1.16	1.49
Pig	*SLA-1*	125 (22.89)	17 (3.11)	56 (10.26)	0.30	1.54	1.66
	*SLA-2*	135 (24.73%)	29 (5.31%)	59 (10.81%)	0.49	1.16	1.86
	*SLA-3*	105 (19.23%)	15 (2.75%)	71 (13.00%)	0.21	2.27	1.49
	Mean	121.67 (22.28%)	20.33 (3.72%)	62.00 (11.3%)	0.34	1.66	1.67
Sheep	*Ovar-N* ^c^	142 (26.01)	26 (4.76)	61 (11.17)	0.43	1.41	1.93

^a^ The number within parentheses indicates the percentage of variable sites across the entire peptide-binding region, 546 bp in size. ^b^ Numbers within parentheses indicate the percentages of synonymous or nonsynonymous substitution sites across the entire peptide-binding region. ^c^ indicates the combined results of all sheep MHC class I alleles because of the unavailability of annotated allelic information for individual classical MHC class I genes. Note. *SLA*, Swine leukocyte antigen; *BoLA*, Bovine leukocyte antigen; *Patr*, Pan troglodytes leukocyte antigen; *HLA*, Human leukocyte antigen; *Ovar*, Ovine leukocyte antigen.

**Table 4:** Sequence diversity of the peptide-binding pockets of classical MHC class I genes of pigs, cattle, sheep, chimpanzees, and humans.

Shannon Diversity Index (H)						
Locus	Peptide-Binding Pockets ^a^					
	A	B	C	D	E *	F
*SLA-1*	0.65	1.12	1.48	1.01	1.59	0.74
*SLA-2*	0.58	1.18	1.55	1.16	1.75	0.88
*SLA-3*	0.38	0.63	0.96	0.68	1.3	0.66
Mean	0.54	0.98	1.33	0.95	1.55	0.76
*BoLA-1*	0.39	0.72	1.15	1.55	1.75	0.7
*BoLA-2*	0.72	1.34	1.2	1.65	1.89	0.66
*BoLA-3*	0.69	0.98	1.23	1.28	1.7	0.85
Mean	0.6	1.01	1.19	1.49	1.78	0.74
*Patr-A*	0.37	0.66	0.91	0.9	1.12	0.28
*Patr-B*	0.41	1.05	0.92	0.84	1.49	0.8
*Patr-C*	0.44	0.42	0.55	0.86	1.36	0.48
Mean	0.41	0.71	0.79	0.87	1.32	0.46
*HLA-A*	0.47	0.58	0.78	0.78	1.38	0.47
*HLA-B*	0.45	1.07	0.97	0.64	1.08	0.88
*HLA-C*	0.2	0.47	0.64	0.81	1.01	0.57
Mean	0.37	0.71	0.8	0.74	1.15	0.64
*Ovar-N*	0.89	1.13	1.19	1.24	1.81	0.88

^a^ Peptide-binding pockets of non-human MHC class I genes were determined based on those of *HLA* genes. Note: The level of sequence variation in pockets was compared using the Shannon diversity index. Statistical significance of the differences between different peptide-binding pockets is indicated with “*” (*p* < 0.05). *SLA*, Swine leukocyte antigen; *BoLA*, Bovine leukocyte antigen; *Patr*, Pan troglodytes leukocyte antigen; *HLA*, Human leukocyte antigen; *Ovar*, Ovine leukocyte antigen. *Ovar-N* indicates the combined results of all sheep MHC class I alleles, owing to the unavailability of annotated allelic information for individual classical MHC class I genes.

## Data Availability

Data are contained within the article and Supplementary Materials.

## Supplementary Materials

The following supporting information can be downloaded at: https://www.mdpi.com/article/10.3390/genes15010007/s1, Figure S1: Alignment of the exons 2 and 3 sequences (546 bp) of 238 representative MHC class I alleles of 20 different allelic groups for each gene from pigs, cattle, chimpanzees, and humans together with a platypus sequence; Table S1: The number of annotated alleles for each MHC class I gene of pigs, cattle, sheep, chimpanzees, and humans available in IPD; Table S2 The list of alleles in different allelic groups of MHC class I genes from different species; Table S3: Pairwise nucleotide sequence differences in the exons 2 and 3 region of the classical MHC class I genes of pigs, cattle, chimpanzees, and humans; Table S4: Comparison of the levels of estimated genetic diversity for classical MHC class I genes of humans, chimpanzees, cattle and pigs between the analysis using all allelic groups and 20 representative allelic groups; Table S5: Identified variable sites for the peptide-binding region of classical MHC class I genes of pigs, cattle, sheep, chimpanzee, and human; Table S6: Identified peptide-binding pockets of MHC class I proteins based on the structure of *HLA* class I; Table S7: Comparison of the amino-acid sequence diversity among the predicted peptide-binding pocket sites of classical MHC class I genes of pigs, cattle, chimpanzee, and human; Table S8: The frequency of amino acid of PBR from *BoLA-2* selected 20 alleles; Table S9: The frequency of amino acid of PBR from *SLA-1* selected 20 alleles; Table S10: The frequency of amino acid of PBR from *SLA-2* selected 20 alleles; Table S11. The frequency of amino acids in the PBR from *SLA-3* was selected as 20 alleles.
