# Supplementary material for: Genetic Diversity and Sequence Conservation of Peptide-Binding Regions of MHC Class I Genes in Pig, Cattle, Chimpanzee, and Human
Source: Genes (Basel). 2023 Dec 20;15(1):7. doi: 10.3390/genes15010007 (PMC10815642; doi:10.3390/genes15010007)
Supplement: Supplementary file 1 [file genes-15-00007-s001.zip › genes-2774059-supplementary.pdf]

		20		40		60		80
BoLA-1*007:01:01:01	SHSLKYFHTA	VSRPGDGEPR	FITVGYVDDT	QFVRFDSDAP	DPRKEPRAPW	I-EKEGPE--	-YWDRETRIS	KENTLVYRGS
BoLA-1*007:01:01:02	SHSLKYFHTA	VSRPGDGEPR	FITVGYVDDT	QFVRFDSDAP	DPRKEPRAPW	I-EKEGPE--	-YWDRETRIS	KENTLVYRGS
BoLA-1*009:01	SHSLRYFYTA	VSRPGLGEPR	FISVGYVDDT	QFVRFDSDAP	DPRMEPRAPW	I-EKEGPE--	-YWDRETRIS	KENTLVYRGS
BoLA-1*009:02	SHSLRYFYTA	VSRPGLGEPR	FISVGYVDDT	QFVRFDSDAP	NPREEPRAPW	I-EKEGPE--	-YWDRETRIS	KENTLVYRES
BoLA-1*019:01	SHSLKYFHTA	VSRPGDGEPR	FITVGYVDDT	QFVRFDSDAP	DPRKEPRAPW	I-EKEGPE--	-YWDRETRIS	KENTLVYRGS
BoLA-1*020:01	SHSLKYFHTA	VSRPGDGEPR	FITVGYVDDT	QFVRFDSDAP	DPRKEPRAPW	I-EKEGPE--	-YWDRETRIS	KENTLVYRGS
BoLA-1*021:01	SHSLRYFYTA	VSRPGDGEPR	FITVGYVDDT	QFVWFDSDAP	DPRKEPRTPW	I-EKEGPE--	-YWDRETRIS	KENTLVYRGS
BoLA-1*023:01	SHSLRYFYTA	VSRPGLGEPR	FISVGYVDDT	QFVRFDSDAP	NPREEPRAPW	I-EKEGPE--	-YWDRETRIS	KENTLVYRES
BoLA-1*028:01	SHSLRYFYTA	VSRPGDGEPR	FITVGYVDDT	QFVWFDSDAP	DPRKEPRTPW	I-EKEGPE--	-YWDRETRIS	KEKTLLYREN
BoLA-1*029:01	SHSMKYFYTA	VSRPGDGEPR	FITVGYVDDT	EFVRFDSDAP	DPRREPRAPW	I-EKEGPE--	-YWDRETRIS	KENTLLYRKN
BoLA-1*031:01	SHSLKYFYTA	VSRPGDGEPR	FITVGYVDDT	QFVRFDSDAP	DPRKEPRAPW	I-EKEGPE--	-YWDDETRIS	KENTLLYRKN
BoLA-1*031:02	SHSLKYFYTA	VSRPGDGEPR	FITVGYVDDT	QFVRFDSDAP	DPRKEPRAPW	I-EKEGPE--	-YWDDETRIS	KENTLLYRKD
BoLA-1*042:01	SHSLKYFHTA	VSRPGDGEPR	FITVGYVDDT	QFVRFDSDAP	DPRKEPRAPW	I-EKEGPE--	-YWDDETRIS	KENTLLYRKD
BoLA-1*049:01	SHSLRYFYTG	VSRPGLGEPR	FIAVGYVDDT	QFTRFDSDAP	NPREEPRVPW	M-EQEGPE--	-YWDRETRIS	KDTAQSFRVG
BoLA-1*061:01	SHSLRYFYTG	VSRPGDGEPR	FITVGYVDDT	QFVWFDSDAP	DPRIEPRTPW	I-EKEGPE--	-YWDRETRIS	KENTLVYRES
BoLA-1*067:01	SHSLRYFYTG	VSRPGLGEPR	FFAVGYVDDT	QIARFDSDAW	NPRMEPRAPW	M-EQKGPE--	-YWEEMTRDA	KEDQQRSQLC
BoLA-1*074:01	SHSMKYFYTA	VSRPGLGEPR	FITVGYVDDT	QFVWFDSDAP	DPRKEPRAPW	I-EKEGPE--	-YWDRETRIS	KENTLLYRKN
BoLA-1*097:01	SHSLRYFYTG	VSRPGDGEPR	FITVGYVDDT	QFVWFDSDAP	DPRMEPRTPW	I-EKEGPE--	-YWDRETRIS	KENTLVYREG
BoLA-2*005:01	SHSLRYFYTG	VSRPGLGEPR	FIAVGYVDDT	QFVRFDSDAP	NPREEPRVPW	M-EQEGPE--	-YWDRNTRIY	KDTAQIFRVD
BoLA-2*006:01	SHSLRYFSTA	VSRPGLGEPR	FIAVGYVDDT	QFTRFDSDAP	NPREEPRAPW	M-EQEGLE--	-YWDRNTRIY	KDTAQTFRVY
BoLA-2*008:01	SHSLRYFLTA	VSRPGLGEPR	FIIVGYVDDT	QFVRFDSNTP	NPRMEPRARW	V-EKEGPE--	-YWDRETRNS	KETAQTFRAN
BoLA-2*016:01	SHSLRYFSTA	VSRPGLGEPR	FIAVGYVDDT	QFTRFDSDAP	NPREEPRAPW	M-EQEGLE--	-YWDRNTRIY	KDTAQTFRVY
BoLA-2*018:01	SHSLRYFYTA	VSRPGLGEPR	FIAVGYVDDT	QFTRFDSDAP	NPRDEPRVPW	M-EQEGPE--	-YWDRNTRIY	KDTAQIFRAN
BoLA-2*022:01	SHSLRYFHTA	VSRPGLGEPR	FISVGYVDDT	QFVRFDSDSA	NPREEPRAPW	M-EQEGPE--	-YWDEQTRIV	KDTAQSFRVG
BoLA-2*025:01	SHSLRYFSTA	VSRPGLGEPR	FIAVGYVDDT	QFTRFDSDAP	NPREEPRVPW	M-EQEGLE--	-YWDRNTRIY	KDTAQTFRVY
BoLA-2*030:01	SHSLKYFYTA	VSRPGLGEPR	FISVGYVDDT	QFVWFDSDAP	NPREEPRAPW	M-EQEGLE--	-YWDRNTRIY	KDTAQNFRVS
BoLA-2*032:02	SHSLRYFYTG	VSRPGLGEPR	FIAVGYVDDT	QFVRFDSDAP	DPRTEPRARW	V-EQEGPE--	-YWDRETRNL	KGAAQTFRVD
BoLA-2*043:01	SHSMRYFSTA	VSRPGFGEPR	YLEVGYVDDT	QFVRFDSDAP	NPRMEPRARW	V-EQEGPE--	-YWDRETQRA	KGNAQTFRVN
BoLA-2*046:01	SHSMRYFSTA	VSRPGFGEPR	YLEVGYVDDT	QFVRFDSDAP	NPRMEPRARW	V-EQEGPE--	-YWDRETQRA	KGNAQTFRVN
BoLA-2*047:01	SHSMRYFSTA	VSRPGFGEPR	YLEVGYVDDT	QFVRFDSDAP	NPRMEPRVRW	M-EQEGPE--	-YWDQETQRA	KGNAQTFRVD
BoLA-2*048:01	SHSLRYFYTA	VSRPGLGEPR	FISVGYVDDT	QFVRFDSDSA	NPREEPRAPW	M-EQEGPE--	-YWDEQTRIV	KDTAQSFRVG
BoLA-2*054:01	SHSLRYFYTG	VSRPGLGEPR	FIIVGYVDDT	QFVRFDSNTP	NPRMEPRARW	V-EQEGPE--	-YWDQETRNS	KGAAQTFRAN
BoLA-2*056:01	SHSLRYFYTG	VSRPGLGEPR	FIAVGYVDDT	QFVRFDSDAA	DPRTEPRVPW	M-EQEGPE--	-YWDQETRNF	KDAAQTFRAN
BoLA-2*057:01	SHSLRYFYTA	VSRPGLGEPS	FIIVGYVDDT	QFVRFDSNTP	NPRMEPRARW	V-EQEGPE--	-YWDRETRIS	KETAQTFRVD
BoLA-2*060:01	SHSLRYFSTA	VSRPGLGEPR	FIAVGYVDDT	QFTRFDSDAP	NPREEPRAPW	M-EQEGLE--	-YWDRNTRIY	KDTAQTFRVY
BoLA-2*062:01	SHSLRYFYTG	VSRPGLGEPR	FIAVGYVDDT	QFVRFDSDAP	DPRTEPRVRW	V-EQEGPE--	-YWDQETRIQ	KENTQTFRAN
BoLA-2*075:01	SHSLRYFYTG	VSRPGLGEPR	FIIVGYVDDT	QFVRFDSNTP	NPRMEPRARW	V-EQEGPE--	-YWDRNTRNA	KDAAQTFRAN
BoLA-2*099:01	SHSLRYFYTG	VSRPGLGEPR	FIAVGYVDDT	QFVRFDSDAP	DPRTEPRVRW	V-EQEGPE--	-YWDRETRNL	KDAAQTFRVN
BoLA-3*001:03	SHSMRYFSTA	VSRPGLGEPR	FIIVGYVDDT	QFVRFDSDAP	NPRMEPRARW	V-EQEGPE--	-YWDRETQRA	KGNAQFFRVS
BoLA-3*002:01	SHSMRYFLTA	VSRPGFGEPR	YLEVGYVDDT	QFVRFDSDAP	NPRMEPRARW	V-EQEGPE--	-YWDQETRKA	KGNAQFFRVS
BoLA-3*010:01	SHSLRYFYTA	VSRPGLGEPR	FISVGYVDNT	EFVRFDSDAP	DPREEPRVRW	M-EQEGLE--	-YWDRNTRIY	KDTAQTFRVD
BoLA-3*011:01	SHSMRYFSTA	VSRPGLGEPR	YLEVGYVDDT	QFVRFDSDAP	NPRMEPRARW	V-EQEGPE--	-YWDQETRKA	KGTAQTFRAN
BoLA-3*017:01	SHSMRYFSTA	VSRPGLGEPR	YLEVGYVDDT	QFVRFDSDAR	NPRMEPRKRW	V-EQEGPE--	-YWDRETQRA	KGNAQIFRVS
BoLA-3*027:01	SHSMRYFSTA	VSRPGFGEPR	YLEVGYVDDT	QFVRFDSDAR	NPRMEPRTRW	V-KQEGPE--	-YWDRNTRNA	KGNAQSFRVG
BoLA-3*035:01	SHSMRYFYTA	VSRPGLGEPR	FINVGYVDDT	QFVRFDSDAP	NPRMEPRARW	V-EQEGPE--	-YWDQETRNA	KGNAQTFRVG
BoLA-3*036:01	SHSLRYFYTG	VSRPGLGEPR	FIAVGYVDDT	QFVRFDSDAP	DPRMEPRARW	V-EQEGPE--	-YWDRNTRNA	KDAAQTFRVN
BoLA-3*038:01	SHSMRYFNTA	VSRPGFGEPR	YLEVGYVDDT	QFVRFDSDAP	NPRMEPRARW	V-EQEGPE--	-YWDRNTRNA	KGNAQDSRVG
BoLA-3*050:01	SHSMRYFSTA	VSRPGLGEPR	YLEVGYVDDT	QFVRFDSDAP	NPRMEPRTRW	V-KQEGPE--	-YWDRNTRNA	KGNAQTFRGS
BoLA-3*051:01	SHSMRYFSTA	VSRPGFGEPR	YLEVGYVDDT	QFVRFDSDAP	NPRMEPRARW	V-EQEGPE--	-YWDRETQRA	KGNAQTFRVN
BoLA-3*052:01	SHSLRYFYTA	VSRPGLGEPR	FISVGYVDDT	QFVRFDSDAP	NPRMEPRARW	V-EQEGPE--	-YWDRETRNS	KDTAQGFRVD
BoLA-3*058:01	SHSMRYFSTA	VSRPGFGEPR	YLEVGYVDDT	QFVRFDSDAP	NPRMEPRARW	V-EQEGPE--	-YWDRETQRA	KGNAQTFRVG
BoLA-3*059:01	SHSMRYFSTA	VSRPGFGEPR	YLEVGYVDDT	QFVRFDSDAR	NPRMEPRTRW	V-KQEGPE--	-YWDRNTRNA	KGNAQSFRVG
BoLA-3*065:01	SHSMRYFSTA	VSRPGFGEPR	YLEVGYVDDT	QFVRFDSDAP	NPRMEPRARW	V-EQEGPE--	-YWDQETRKA	KGTAQSSRVG
BoLA-3*066:01	SHSLRYFYTA	VSRPGLGEPR	YLEVGYVDDT	QFVRFDSDAP	NPRMEPRTRW	V-KQEGPE--	-YWDQETRKA	KDTAQTFRVD
BoLA-3*068:01	SHSLRYFSTA	VSRPGLGEPR	FIIVGYVDDT	QFVRFDSDAG	DPRVEPRARW	V-EQEGPE--	-YWDQNTRNA	KGTAQTFRAN
BoLA-3*073:01	SHSLRYFYTA	VSRPGLGEPR	FIIVGYVDDT	QFVRFDSDAP	NPRIEPRARW	V-EQEGPE--	-YWDQETRIS	KDTAQSFRVS
BoLA-3*080:01	SHSMRYFSTA	VSRPGFGEPR	YLEVGYVDDT	QFVRFDSDAP	NPRMEPRARW	V-EQEGPE--	-YWDHNTRNS	KDTAQSFRVN
BoLA-3*082:01	SHSLRYFYTA	VSRPDLGEPR	YLQVGYVDDT	QFVRFDSDAP	NPRMEPRARW	V-EQEGPE--	-YWDQETRKA	KDAAQSSRVG
HLA-A*01:01	SHSMRYFFTS	VSRPGRGEPR	FIAVGYVDDT	QFVRFDSDAA	SQKMEPRAPW	I-EQEGPE--	-YWDQETRNM	KAHSQTDRAN
HLA-A*02:01	SHSMRYFFTS	VSRPGRGEPR	FIAVGYVDDT	QFVRFDSDAA	SQRMEPRAPW	I-EQEGPE--	-YWDGETRKV	KAHSQTHRVD
HLA-A*03:01	SHSMRYFFTS	VSRPGRGEPR	FIAVGYVDDT	QFVRFDSDAA	SQRMEPRAPW	I-EQEGPE--	-YWDQETRNV	KAQSQTDRVD
HLA-A*11:01	SHSMRYFYTS	VSRPGRGEPR	FIAVGYVDDT	QFVRFDSDAA	SQRMEPRAPW	I-EQEGPE--	-YWDQETRNV	KAQSQTDRVD
HLA-A*23:01	SHSMRYFSTS	VSRPGRGEPR	FIAVGYVDDT	QFVRFDSDAA	SQRMEPRAPW	I-EQEGPE--	-YWDEETGKV	KAHSQTDREN
HLA-A*24:02	SHSMRYFSTS	VSRPGRGEPR	FIAVGYVDDT	QFVRFDSDAA	SQRMEPRAPW	I-EQEGPE--	-YWDEETGKV	KAHSQTDREN
HLA-A*25:01	SHSMRYFYTS	VSRPGRGEPR	FIAVGYVDDT	QFVRFDSDAA	SQRMEPRAPW	I-EQEGPE--	-YWDRNTRNV	KAHSQTDRES
HLA-A*26:01	SHSMRYFYTS	VSRPGRGEPR	FIAVGYVDDT	QFVRFDSDAA	SQRMEPRAPW	I-EQEGPE--	-YWDRNTRNV	KAHSQTDRAN
HLA-A*29:01	SHSMRYFTTS	VSRPGRGEPR	FIAVGYVDDT	QFVRFDSDAA	SQRMEPRAPW	I-EQEGPE--	-YWDLQTRNV	KAQSQTDRAN
HLA-A*30:01	SHSMRYFSTS	VSRPGSGEPR	FIAVGYVDDT	QFVRFDSDAA	SQRMEPRAPW	I-EQERPE--	-YWDQETRNV	KAQSQTDRVD
HLA-A*31:01	SHSMRYFTTS	VSRPGRGEPR	FIAVGYVDDT	QFVRFDSDAA	SQRMEPRAPW	I-EQERPE--	-YWDQETRNV	KAHSQIDRVD
HLA-A*32:01	SHSMRYFFTS	VSRPGRGEPR	FIAVGYVDDT	QFVRFDSDAA	SQRMEPRAPW	I-EQEGPE--	-YWDQETRNV	KAHSQTDRES
HLA-A*33:01	SHSMRYFTTS	VSRPGRGEPR	FIAVGYVDDT	QFVRFDSDAA	SQRMEPRAPW	I-EQEGPE--	-YWDRNTRNV	KAHSQIDRVD
HLA-A*34:01	SHSMRYFYTS	VSRPGRGEPR	FIAVGYVDDT	QFVRFDSDAA	SQRMEPRAPW	I-EQEGPE--	-YWDRNTRKV	KAQSQTDRVD
HLA-A*36:01	SHSMRYFFTS	VSRPGRGEPR	FIAVGYVDDT	QFVRFDSDAA	SQKMEPRAPW	I-EQEGPE--	-YWDQETRNM	KAHSQTDRAN
HLA-A*43:01	SHSMRYFYTS	VSRPGRGEPR	FIAVGYVDDT	QFVRFDSDAA	SQRMEPRAPW	I-EQEGPE--	-YWDLQTRNV	KAHSQTDRAN
HLA-A*66:01	SHSMRYFYTS	VSRPGRGEPR	FIAVGYVDDT	QFVRFDSDAA	SQRMEPRAPW	I-EQEGPE--	-YWDRNTRNV	KAQSQTDRVD
HLA-A*68:01	SHSMRYFYTS	VSRPGRGEPR	FIAVGYVDDT	QFVRFDSDAA	SQRMEPRAPW	I-EQEGPE--	-YWDRNTRNV	KAQSQTDRVD
HLA-A*69:01	SHSMRYFYTS	VSRPGRGEPR	FIAVGYVDDT	QFVRFDSDAA	SQRMEPRAPW	I-EQEGPE--	-YWDRNTRNV	KAQSQTDRVD
HLA-A*80:01	SHSMRYFFTS	VSRPGRGEPR	FIAVGYVDDS	QFVQFDSDAA	SQRMEPRAPW	I-EQEEPE--	-YWDEETRNV	KAHSQTNRAN
HLA-B*07:02:01:01	SHSMRYFYTS	VSRPGRGEPR	FISVGYVDDT	QFVRFDSDAA	SPREEPRAPW	I-EQEGPE--	-YWDRNTQIY	KAQAQTDRES
HLA-B*13:01:01:01	SHSMRYFYTA	MSRPGRGEPR	FITVGYVDDT	QFVRFDSDAT	SPRMAPRAPW	I-EQEGPE--	-YWDRETQIS	KTNTQTYREN
HLA-B*27:01	SHSMRYFHTS	VSRPGRGEPR	FITVGYVDDT	LFVRFDSDAA	SPREEPRAPW	I-EQEGPE--	-YWDRETQIC	KAKAQTYREN
HLA-B*37:01:01:01	SHSMRYFHTS	VSRPGRGEPR	FISVGYVDDT	QFVRFDSDAA	SPRTEPRAPW	I-EQEGPE--	-YWDRETQIS	KTNTQTYRED
HLA-B*38:01:01:01	SHSMRYFYTS	VSRPGRGEPR	FISVGYVDDT	QFVRFDSDAA	SPREEPRAPW	I-EQEGPE--	-YWDRNTQIC	KTNTQTYREN
HLA-B*40:01:01	SHSMRYFHTA	MSRPGRGEPR	FITVGYVDDT	LFVRFDSDAT	SPRKEPRAPW	I-EQEGPE--	-YWDRETQIS	KTNTQTYRES
HLA-B*41:01:01:01	SHSMRYFHTA	MSRPGRGEPR	FITVGYVDDT	LFVRFDSDAT	SPRKEPRAPW	I-EQEGPE--	-YWDRETQIS	KTNTQTYRES
HLA-B*42:01:01:01	SHSMRYFYTS	VSRPGRGEPR	FISVGYVDDT	QFVRFDSDAA	SPREEPRAPW	I-EQEGPE--	-YWDRNTQIY	KAQAQTDRES
HLA-B*45:01:01:01	SHSMRYFHTA	MSRPGRGEPR	FITVGYVDDT	LFVRFDSDAT	SPRKEPRAPW	I-EQEGPE--	-YWDRETQIS	KTNTQTYRES
HLA-B*46:01:01:01	SHSMRYFYTA	MSRPGRGEPR	FIAVGYVDDT	QFVRFDSDAA	SPRMAPRAPW	I-EQEGPE--	-YWDRETQKY	KRQAQTDRVS
HLA-B*47:01:01:02	SHSMRYFYTA	MSRPGRGEPR	FITVGYVDDT	LFVRFDSDAT	SPRKEPRAPW	I-EQEGPE--	-YWDRETQIS	KTNTQTYRED
HLA-B*49:01:01:01	SHSMRYFHTA	MSRPGRGEPR	FITVGYVDDT	LFVRFDSDAT	SPRKEPRAPW	I-EQEGPE--	-YWDRETQIS	KTNTQTYREN
HLA-B*53:01:01:01	SHSMRYFYTA	MSRPGRGEPR	FIAVGYVDDT	QFVRFDSDAA	SPRTEPRAPW	I-EQEGPE--	-YWDRNTQIF	KTNTQTYREN

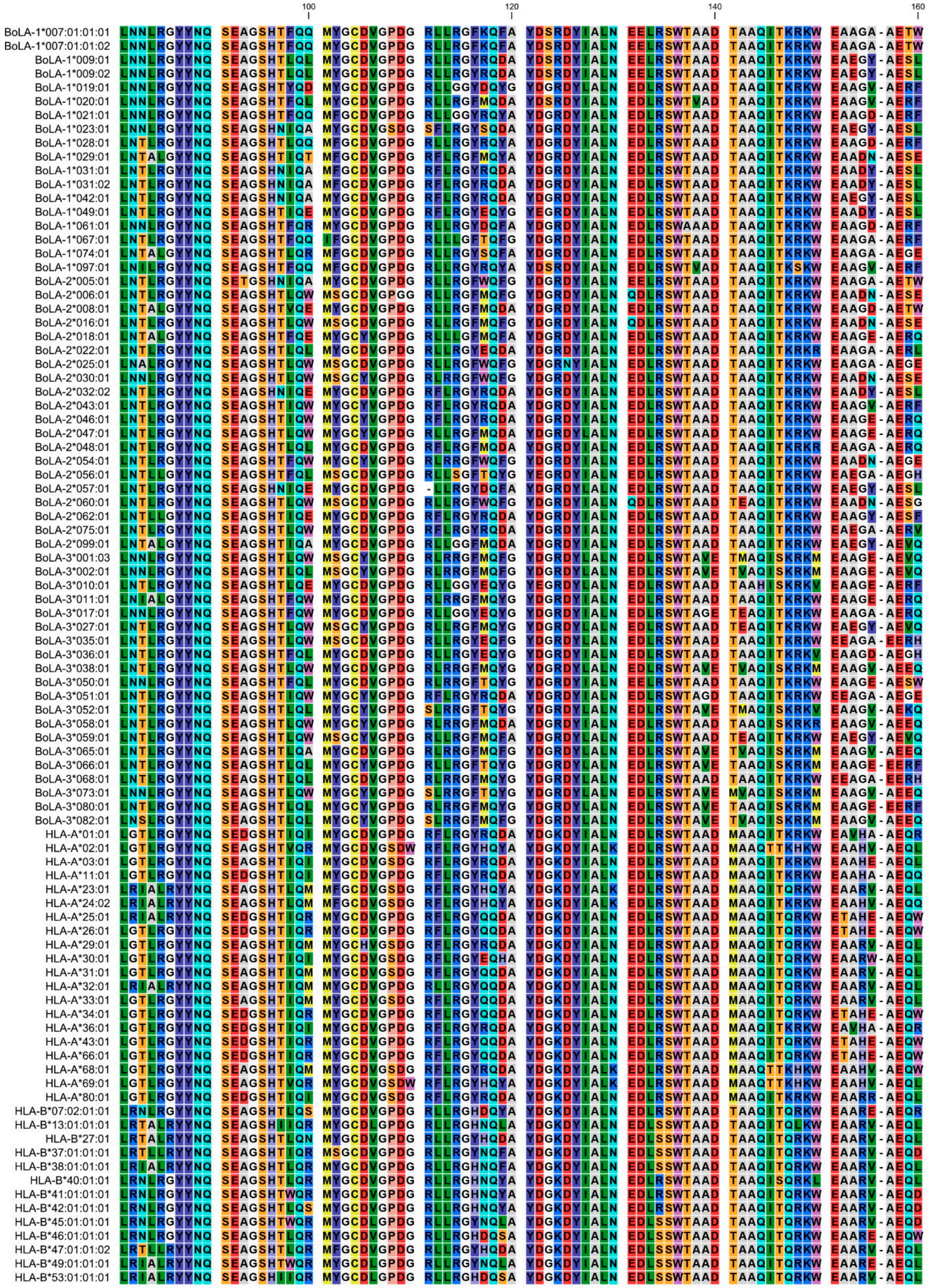
100
120
140
160
BoLA-1*007:01:01:01 LNNLRGYYNQ SEAGSHTFQQ MYGCDVGPDG RLLRGFKQFA YDSRDYIALN EELRSWTAAD TAAQITKRKW EAAGA-AETW
BoLA-1*007:01:01:02 LNNLRGYYNQ SEAGSHTFQQ MYGCDVGPDG RLLRGFKQFA YDSRDYIALN EELRSWTAAD TAAQITKRKW EAAGA-AETW
BoLA-1*009:01 LNNLRGYYNQ SEAGSHTIQL MYGCDVGPDG RLLRGYRQDA YDSRDYIALN EELRSWTAAD TAAQITKRKW EAEGY-AESL
BoLA-1*009:02 LNNLRGYYNQ SEAGSHTIQL MYGCDVGPDG RLLRGYRQDA YDSRDYIALN EELRSWTAAD TAAQITKRKW EAEGY-AESL
BoLA-1*019:01 LNNLRGYYNQ SEAGSHTYQD MYGCDVGSDG RLLGGYDQYG YDGRDYLALN EDLRSWTAAD TAAQITKRKW EAAGV-AERF
BoLA-1*020:01 LNNLRGYYNQ SEAGSHTFQL MYGCDVGPDG RLLRGFMQDA YDSRDYIALN EDLRSWTVAD TAAQITKRKW EAAGV-AERF
BoLA-1*021:01 LNNLRGYYNQ SEAGSHTFQQ MFGCDVGPDG RLLGGYRQYA YDGRDYIALN EDLRSWTAAD TAAQITKRKW EAAGD-AERF
BoLA-1*023:01 LNNLRGYYNQ SEAGSHNIQA MYGCDVGSDG SFLRGYSQDA YDGRDYIALN EDLRSWTAAD TAAQITKRKW EAEGY-AESL
BoLA-1*028:01 LNTLRGYYNQ SEAGSHTLQQ MFGCDVGPDG RLLRGYRQYA YDGRDYIALN EDLRSWTAAD TAAQITKRKW EAAGD-AERF
BoLA-1*029:01 LNTALGYYNQ SEAGSHTIQT MFGCDVGPDG RFLRGFMQYA YDGRDYIALN EDLRSWTAAD TAAQITKRKW EAADN-AESE
BoLA-1*031:01 LNTLRGYYNQ SEAGSHNIQA MFGCDVGPDG RFLRGYRQDA YDGRDYIALN EDLRSWTAAD TAAQITKRKW EAADY-AESL
BoLA-1*031:02 LNTLRGYYNQ SEAGSHNIQA MFGCDVGPDG RFLRGYRQDA YDGRDYIALN EDLRSWTAAD TAAQITKRKW EAADY-AESL
BoLA-1*042:01 LNTLRGYYNQ SEAGSHNIQA MYGCDVGPDG RFLRGYRQDA YDGRDYIALN EDLRSWTAAD TAAQITKRKW EAEGY-AESL
BoLA-1*049:01 LNTLRGYYNQ SEAGSHTIQE MYGCDVGPDG RFLRGYEQYG YEGRDYIALN EDLRSWTAAD TAAQITKRKW EAADY-AESL
BoLA-1*061:01 LNNLRGYYNQ SEAGSHTFQR MYGCDVGPDG RLLRGYDQFA YDGRDYIALN EDLRSWAAAD TAAQITKRKW EAAGD-AERF
BoLA-1*067:01 LNTLRGYYNQ SEAGSHTFQQ IFGCDVGPDG RLLLGFTQFG YDGRDYIALN EDLRSWTAAD TAAQITKRKW EAAGA-AERF
BoLA-1*074:01 LNTALGYYNQ SEAGSHTLQR MFGCDVGPDG RLLRGYSQFA YDGRDYIALN EDLRSWTAAD TAAQITKRKW EAAGA-AEGE
BoLA-1*097:01 LNILRGYYNQ SEAGSHTFQQ MFGCDVGPDG RLLRGYRQYA YDSRDYIALN EDLRSWTVAD TAAQITKSKW EAAGV-AERF
BoLA-2*005:01 LNTLRGYYNQ SETGSHNIQA MYGCDVGPDG RLLRGFWQFG YDGRDYIALN EELRSWTAAD TAAQITKRKW EAAGA-AETW
BoLA-2*006:01 LNTLRGYYNQ SEAGSHTLQW MSGCDVGPGG RLLRGFMQFG YDGRDYIALN QDLRSWTAAD TAAQITKRKW EAADN-AESE
BoLA-2*008:01 LNTALGYYNQ SEAGSHTVQE MYGCDVGPDG RLLRGFMQDA YDGRDYIALN EDLRSWTAAD TAAQITKRKW EAAGD-AETW
BoLA-2*016:01 LNTLRGYYNQ SEAGSHTLQW MSGCDVGPDG RLLRGFMQFG YDGRDYIALN QDLRSWTAAD TAAQITKRKW EAADN-AESE
BoLA-2*018:01 LNTALGYYNQ SEAGSHTFQE MYGCYVGPDG RLLLGFMQFA YDGRDYIALN EDLRSWTAAD TAAQITKRKW EAAGE-AERQ
BoLA-2*022:01 LNTLRGYYNQ SEAGSHTLQL MYGCDVGPDG RLLRGYEQDA YDGRDYIALN EDLRSWTAAD TAAQITKRKR EAAGA-AERL
BoLA-2*025:01 LNALRGYYNQ SEAGSHTLQW MSGCDVGPDG RLLRGFWQFG YDGRNYIALN EDLRSWTAAD TAAQITKRKW EAAGA-AEGE
BoLA-2*030:01 LNNLRGYYNQ SEAGSHTIQW MSGCYVGPDG RLRRGFWQFG YDGRDYIALN EDLRSWTAAD TAAQITKRKW EAADN-AESE
BoLA-2*032:02 LNTLRGYYNQ SEAGSHNIQE MYGCDVGPDG RFLRGYRQDA YDGRDYIALN EDLRSWTAAD TAAQITKRKW EAADY-AESL
BoLA-2*043:01 LNTLRGYYNQ SEAGSHTIQW MYGCYVGPDG RFLRGYRQDA YDGRDYIALN EDLRSWTAAD TAAQITKRKW EAAGV-AERF
BoLA-2*046:01 LNTLRGYYNQ SEAGSHTIQW MYGCYVGPDG RFLRGYRQDA YDGRDYIALN EDLRSWTAAD TAAQITKRKW EAAGE-AERQ
BoLA-2*047:01 LNTLRGYYNQ SEAGSHTIQW MYGCYVGPDG RLLRGFMQDA YDGRDYIALN EDLRSWTAAD TAAQITKRKW EAAGE-AERQ
BoLA-2*048:01 LNTLRGYYNQ SEAGSHTLQL MYGCDVGPDG RLLRGFMQDA YDGRDYIALN EDLRSWTAAD TAAQITKRKR EAAGA-AERQ
BoLA-2*054:01 LNTLRGYYNQ SEAGSHTFQW MYGCYVGPDG RLRRGFWQFG YDGRDYIALN EDLRSWTAAD TAAQITKRKW EAADN-AEGE
BoLA-2*056:01 LNTLLGYYNQ SEAGSHTFQL MSGCDVGPDG RLLSGFTQYG YEGRDYIALN EDLRSWTAAD TAAQITKRKW EAEGA-AEGH
BoLA-2*057:01 LNTLRGYYNQ SEAGSHNIQE MYGCDVGPDG -LLRGYDQFA YDGRDYLALN EDLRSWTAAD TAAQITKRKW EAEGY-AESL
BoLA-2*060:01 LNTLRGYYNQ SEAGSHTLQW MSGCDVGPDG RLLRGFWQFG YDGRDYIALN QDLRSWTAAD TEAQITKRKW EAADN-AESG
BoLA-2*062:01 LNTLLGYYNQ SEAGSHTIQE MYGCDVGPDG RFLRGYRQDA YDGRDYIALN EDLRSWTAAD TAAQITKRKW EAAGY-AESF
BoLA-2*075:01 LNTLRGYYNQ SEAGSHTLQW MYGCDVGPDG RFLRGYRQDA YDGRDYIALN EDLRSWTAAD TAAQITKRKW EAEGA-AERV
BoLA-2*099:01 LNTALGYYNQ SEAGSHTIQA MYGCDVGPDG RLLGGFMQDA YDGRDYIALN EDLRSWTAAD TAAQITKRKW EAEGY-AEVQ
BoLA-3*001:03 LNNLRGYYNQ SEAGSHTLQW MSGCYVGPDG RLRRGFMQFG YDGRDYLALN EDLRSWTAVE TMAQISKRKM EAAGE-AEVQ
BoLA-3*002:01 LNNLRGYYNQ SEAGSHTLQL MSGCYVGPDG RLRRGFMQFG YDGRDYLALN EDLRSWTAVE TVAQISKRKM EAAGE-AEVQ
BoLA-3*010:01 LNTLRGYYNQ SEAGSHTLQE MYGCDVGPDG RLLGGYEQYG YEGRDYLALN EDLRSWTAAD TAAHISKRKV EAAGE-AERF
BoLA-3*011:01 LNIALGYYNQ SEAGSHTFQW MYGCDVGPDG RLRRGFMQYG YDGRDYIALN EDLRSWTAAD TAAQITKRKW EAAGE-AERQ
BoLA-3*017:01 LNNLRGYYNQ SEAGSHTFQW MYGCDVGPDG RLLGGYEQYG YDGRDYIALN EDLRSWTAGE TEAQITKRKW EAAGA-AERQ
BoLA-3*027:01 LNTLRGYYNQ SEAGSHTLQW MSGCYVGPDG RLLRGFMQYG YDGRDYLALN EDLRSWTAAD TEAQITKRKW EAEGY-AEVQ
BoLA-3*035:01 LNTLRGYYNQ SEAGSHTLQW MSGCDVGPDG RLLRGYEQFG YDGRDYIALN EDLRSWTAAD TAAQITKRKW EEAGA-EERH
BoLA-3*036:01 LNTLRGYYNQ SEAGSHTFQL MYGCDVGPDG RLLRGYEQYG YDGRDYIALN EDLRSWTAAD TAAQITKRKV EAAGD-AEGH
BoLA-3*038:01 LNTLRGYYNQ SEAGSHTLQW MSGCDVGPDG RLRRGFMQYG YDGRDYLALN EDLRSWTAVE TVAQISKRKM EAAGV-AEEQ
BoLA-3*050:01 LNNLRGYYNQ SEAGSHTFQL MYGCDVGPDG RLRRGFTQYG YDGRDYIALN EELRSWTAAD TAAQITKRKW EAAGE-AESW
BoLA-3*051:01 LNTLRGYYNQ SEAGSHTIQW MYGCYVGPDG RFLRGYRQDA YDGRDYIALN EDLRSWTAGD TAAQITKRKW EEAGA-AEGE
BoLA-3*052:01 LNTLRGYYNQ SEAGSHTLQL MYGCYVGPDG SLRRGFTQYG YDGRDYLALN EDLRSWTAVE TMAQISKRKV EAAGV-AEKQ
BoLA-3*058:01 LNTLRGYYNQ SEAGSHTIQW MYGCYVGPDG RLRRGFMQDA YDGRDYIALN EDLRSWTAAD TAAQISKRKR EAAGV-AEEQ
BoLA-3*059:01 LNTLRGYYNQ SEAGSHTLQW MSGCYVGPDG RLLRGFMQFA YDGRDYIALN EDLRSWTAAD TEAQITKRKW EAEGY-AEVQ
BoLA-3*065:01 LNTLRGYYNQ SEAGSHTLQA MYGCDVGPDG RLRRGFMQFG YDGRDYLALN EDLRSWTAVE TVAQISKRKM EAAGV-AEEQ
BoLA-3*066:01 LNTLRGYYNQ SEAGSHTLQL MYGCYVGPDG RLLRGFTQYG YDGRDYLALN EDLRSWTAVE TAAQISKRKM EAAGE-EERF
BoLA-3*068:01 LNTLRGYYNQ SEAGSHTLQL MYGCDVGPDG RLRRGFMQYG YDGRDYIALN EDLRSWTAAD TAAQITKRKW EEAGA-EERH
BoLA-3*073:01 LNNLRGYYNQ SEAGSHTLQW MYGCYVGPDG SLRRGFTQYG YDGRDYLALN EDLRSWTAVE MVAQISKRKM EAAGV-AEEQ
BoLA-3*080:01 LNTLRGYYNQ SEAGSHTLQL MYGCYVGPDG RLRRGFMQYG YDGRDYLALN EDLRSWTAVE TAAQISKRKM EAAGE-EERF
BoLA-3*082:01 LNSLRGYYNQ SEAGSHTLQL MYGCYVGPDG SLRRGFMQFG YDGRDYLALN EDLRSWTAVE TVAQISKRKM EAAGV-AEEQ
HLA-A*01:01 LGTLRGYYNQ SEDGSHTIQI MYGCDVGPDG RFLRGYRQDA YDGKDYIALN EDLRSWTAAD MAAQITKRKW EAVHA-AEQR
HLA-A*02:01 LGTLRGYYNQ SEAGSHTVQR MYGCDVGSDW RFLRGYHQYA YDGKDYIALK EDLRSWTAAD MAAQTTKHKW EAAHV-AEQL
HLA-A*03:01 LGTLRGYYNQ SEAGSHTIQI MYGCDVGSDG RFLRGYRQDA YDGKDYIALN EDLRSWTAAD MAAQITKRKW EAAHE-AEQL
HLA-A*11:01 LGTLRGYYNQ SEDGSHTIQI MYGCDVGPDG RFLRGYRQDA YDGKDYIALN EDLRSWTAAD MAAQITKRKW EAAHA-AEQQ
HLA-A*23:01 LRIALRYYNQ SEAGSHTLQM MFGCDVGSDG RFLRGYHQYA YDGKDYIALK EDLRSWTAAD MAAQITQRKW EAARV-AEQL
HLA-A*24:02 LRIALRYYNQ SEAGSHTLQM MFGCDVGSDG RFLRGYHQYA YDGKDYIALK EDLRSWTAAD MAAQITKRKW EAAHV-AEQQ
HLA-A*25:01 LRIALRYYNQ SEDGSHTIQR MYGCDVGPDG RFLRGYQQDA YDGKDYIALN EDLRSWTAAD MAAQITQRKW ETAHE-AEQW
HLA-A*26:01 LGTLRGYYNQ SEDGSHTIQR MYGCDVGPDG RFLRGYQQDA YDGKDYIALN EDLRSWTAAD MAAQITQRKW ETAHE-AEQW
HLA-A*29:01 LGTLRGYYNQ SEAGSHTLQM MYGCHVGSDG RFLRGYRQDA YDGKDYIALN EDLRSWTAAD MAAQITQRKW EAARV-AEQL
HLA-A*30:01 LGTLRGYYNQ SEAGSHTIQI MYGCDVGSDG RFLRGYEQHA YDGKDYIALN EDLRSWTAAD MAAQITQRKW EAARW-AEQL
HLA-A*31:01 LGTLRGYYNQ SEAGSHTIQM MYGCDVGSDG RFLRGYQQDA YDGKDYIALN EDLRSWTAAD MAAQITQRKW EAARV-AEQL
HLA-A*32:01 LRIALRYYNQ SEAGSHTIQM MYGCDVGPDG RFLRGYQQDA YDGKDYIALN EDLRSWTAAD MAAQITQRKW EAARV-AEQL
HLA-A*33:01 LGTLRGYYNQ SEAGSHTIQM MYGCDVGSDG RFLRGYQQDA YDGKDYIALN EDLRSWTAAD MAAQITQRKW EAARV-AEQL
HLA-A*34:01 LGTLRGYYNQ SEDGSHTIQR MYGCDVGPDG RFLRGYQQDA YDGKDYIALN EDLRSWTAAD MAAQITQRKW ETAHE-AEQW
HLA-A*36:01 LGTLRGYYNQ SEDGSHTIQI MYGCDVGPDG RFLRGYRQDA YDGKDYIALN EDLRSWTAAD MAAQITKRKW EAVHA-AEQR
HLA-A*43:01 LGTLRGYYNQ SEDGSHTIQR MYGCDVGPDG RFLRGYQQDA YDGKDYIALN EDLRSWTAAD MAAQITQRKW ETAHE-AEQW
HLA-A*66:01 LGTLRGYYNQ SEDGSHTIQR MYGCDVGPDG RFLRGYQQDA YDGKDYIALN EDLRSWTAAD MAAQITQRKW ETAHE-AEQW
HLA-A*68:01 LGTLRGYYNQ SEAGSHTIQM MYGCDVGSDG RFLRGYRQDA YDGKDYIALK EDLRSWTAAD MAAQTTKHKW EAAHV-AEQW
HLA-A*69:01 LGTLRGYYNQ SEAGSHTVQR MYGCDVGSDW RFLRGYHQYA YDGKDYIALK EDLRSWTAAD MAAQTTKHKW EAAHV-AEQL
HLA-A*80:01 LGTLRGYYNQ SEDGSHTIQI MYGCDVGSDG RFLRGYRQDA YDGKDYIALN EDLRSWTAAD MAAQITKRKW EAARR-AEQL
HLA-B*07:02:01:01 LRNLRGYYNQ SEAGSHTLQS MYGCDVGPDG RLLRGHDQYA YDGKDYIALN EDLRSWTAAD TAAQITQRKW EAARE-AEQR
HLA-B*13:01:01:01 LRTALRYYNQ SEAGSHIIQR MYGCDLGPDG RLLRGHNQLA YDGKDYIALN EDLSSWTAAD TAAQITQLKW EAARV-AEQL
HLA-B*27:01 LRTALRYYNQ SEAGSHTLQN MYGCDVGPDG RLLRGYHQDA YDGKDYIALN EDLSSWTAAD TAAQITQRKW EAARV-AEQL
HLA-B*37:01:01:01 LRTLLRYYNQ SEAGSHTLQR MSGCDVGPDG RLLRGYNQFA YDGKDYIALN EDLSSWTAAD TAAQITQRKW EAARV-AEQD
HLA-B*38:01:01:01 LRIALRYYNQ SEAGSHTLQR MYGCDVGPDG RLLRGHNQFA YDGKDYIALN EDLSSWTAAD TAAQITQRKW EAARV-AEQL
HLA-B*40:01 LRNLRGYYNQ SEAGSHTLQR MYGCDVGPDG RLLRGHNQYA YDGKDYIALN EDLRSWTAAD TAAQISQRKL EAARV-AEQL
HLA-B*41:01:01:01 LRNLRGYYNQ SEAGSHTWQR MYGCDVGPDG RLLRGHNQYA YDGKDYIALN EDLRSWTAAD TAAQITQRKW EAARV-AEQD
HLA-B*42:01:01:01 LRNLRGYYNQ SEAGSHTLQS MYGCDVGPDG RLLRGHNQYA YDGKDYIALN EDLRSWTAAD TAAQITQRKW EAARV-AEQD
HLA-B*45:01:01:01 LRNLRGYYNQ SEAGSHTWQR MYGCDLGPDG RLLRGYNQLA YDGKDYIALN EDLSSWTAAD TAAQITQRKW EAARV-AEQD
HLA-B*46:01:01:01 LRNLRGYYNQ SEAGSHTLQR MYGCDVGPDG RLLRGHDQSA YDGKDYIALN EDLSSWTAAD TAAQITQRKW EAARE-AEQW
HLA-B*47:01:01:02 LRTLLRYYNQ SEAGSHTLQR MFGCDVGPDG RLLRGYHQDA YDGKDYIALN EDLSSWTAAD TAAQITQRKW EAARV-AEQL
HLA-B*49:01:01:01 LRIALRYYNQ SEAGSHTWQR MYGCDLGPDG RLLRGYNQLA YDGKDYIALN EDLRSWTAAD TAAQITQRKW EAARE-AEQL
HLA-B*53:01:01:01 LRIALRYYNQ SEAGSHIIQR MYGCDLGPDG RLLRGHDQSA YDGKDYIALN EDLSSWTAAD TAAQITQRKW EAARV-AEQL

		180		
BoLA-1*007:01:01:01	RNYLEGECVE	WLRRYLENGK	DTLLRA	181
BoLA-1*007:01:01:02	RNYLEGECVE	WLRRYLENGK	DTLLRA	181
BoLA-1*009:01	RNYLEGRCVE	WLRRYLENGK	DALLRA	181
BoLA-1*009:02	RNYLEGRCVE	WLRRYLENGK	DALLRA	181
BoLA-1*019:01	RNYVEGRCVE	GLRRYLENGK	DALLRA	181
BoLA-1*020:01	RNYVEGRCVE	GLRRYLENGK	DALLRA	181
BoLA-1*021:01	RNYVEGECVE	GLRRYLENGK	DTLLRA	181
BoLA-1*023:01	RNYLEGRCVE	WLRRYLENGK	DALLRA	181
BoLA-1*028:01	RNYVEGECVE	WLRRYLENGK	DTLLRA	181
BoLA-1*029:01	RNYLEGRCVE	GLRRYLENGK	DALLRA	181
BoLA-1*031:01	RNYLEGTCVE	WLRRYLENGK	DTLLRA	181
BoLA-1*031:02	RNYLEGTCVE	WLRRYLENGK	DTLLRA	181
BoLA-1*042:01	RNYLEGRCVE	GLRRYLENGK	DALLRA	181
BoLA-1*049:01	RNYLEGECVE	GLRRYLENGK	DTLLRA	181
BoLA-1*061:01	RNYVEGRCVE	WLRRYLENGK	DALLRA	181
BoLA-1*067:01	RNYLEGTCVE	WLRRYLENGK	DALLRA	181
BoLA-1*074:01	RNYVEGRCVE	GLRRYLENGK	DALLRA	181
BoLA-1*097:01	RNYVEGRCVE	WLRRYLENGK	DALLRA	181
BoLA-2*005:01	RNYLEGECVE	WLRRYLENGK	DTLLRA	181
BoLA-2*006:01	RNYLEGECVE	WLRRYLENGK	DTLLRA	181
BoLA-2*008:01	RNYLEGRCVE	WLRRYLENGK	DALLRA	181
BoLA-2*016:01	RNYLEGECVE	GLRRYLENGK	DALLRA	181
BoLA-2*018:01	RNYLEGRCVE	GLRRYLENGK	DTLLRA	181
BoLA-2*022:01	RNYLEGTCVE	GLRRYLENGK	DALLRA	181
BoLA-2*025:01	RNYVEGRCVE	GLRRYLENGK	DALLRA	181
BoLA-2*030:01	RNYLEGECVE	WLRRYLENGK	DALLRA	181
BoLA-2*032:02	RNYLEGTCVE	WLRRYLENGK	DALLRA	181
BoLA-2*043:01	RNYLEGLCVE	GLRRYLENGK	DTLLRA	181
BoLA-2*046:01	RNYLEGTCVE	WLRRYLENGK	DTLLRA	181
BoLA-2*047:01	RNYLEGTCVE	WLRRYLENGK	DTLLRA	181
BoLA-2*048:01	RNYLEGRCVE	GLRRYLENGK	DALLRA	181
BoLA-2*054:01	RNYVEGRCVE	GLRRYLENGK	DTLLRA	181
BoLA-2*056:01	RNYLEGRCVE	GLRRYLENGK	DALLRA	181
BoLA-2*057:01	RNYLEGRCVE	GLRRYLENGK	DTLLRA	180
BoLA-2*060:01	RNYLEGECVE	GLRRYLENGK	DALLRA	181
BoLA-2*062:01	RNYLEGRCVE	WLRRYLENGK	DTLLRA	181
BoLA-2*075:01	RNYLEGLCVE	WLRRHLENGK	DALLRA	181
BoLA-2*099:01	RNYLEGECVE	WLRRYLETGK	DTLLRA	181
BoLA-3*001:03	RNYLEGRCVE	WLRRYLENGK	DTLLRA	181
BoLA-3*002:01	RNYLEGRCVE	WLRRYLENGK	DTLLRA	181
BoLA-3*010:01	RNYLEGRCVE	GLRRYLENGK	DALLRA	181
BoLA-3*011:01	RNYLEGTCVE	WLRRYLETGK	DTLLRA	181
BoLA-3*017:01	RNYLEGRCVE	WLRRYLENGK	DTLLRA	181
BoLA-3*027:01	RNYLEGECVE	WLRRHLENGK	DTLLRA	181
BoLA-3*035:01	RNYLEGTCVE	WLRRYLENGK	DTLLRA	181
BoLA-3*036:01	RNYLEGRCVE	WLRRYLENGK	DTLLRA	181
BoLA-3*038:01	RNYLEGTCVE	WLRRYLENGK	DTLLRA	181
BoLA-3*050:01	RNYLEGTCVE	WLRRHLENGK	DTLLRA	181
BoLA-3*051:01	RNYLEGTCVE	WLRRYLENGK	DALLRA	181
BoLA-3*052:01	RNYLEGRCVE	WLRRYLENGK	DTLLRA	181
BoLA-3*058:01	RNYLEGRCVE	WLRRYLENGK	DTLLRA	181
BoLA-3*059:01	RNYLEGRCVE	WLRRHLENGK	DTLLRA	181
BoLA-3*065:01	RNYLEGECVE	WLRRYLENGK	DTLLRA	181
BoLA-3*066:01	RNYLEGTCVE	WLRRYLENGK	DTLLRA	181
BoLA-3*068:01	TNYLEGECVE	WLRRYLENGK	DTLLRA	181
BoLA-3*073:01	RNYLEGECVE	WLRRYLENGK	DTLLRA	181
BoLA-3*080:01	RNYLEGTCVE	WLRRHLENGK	DTLLRA	181
BoLA-3*082:01	RNYLEGRCVE	WLRRYLENGK	DTLLRA	181
HLA-A*01:01	RVYLEGRCVD	GLRRYLENGK	ETLQRT	181
HLA-A*02:01	RAYLEGTCVE	WLRRYLENGK	ETLQRT	181
HLA-A*03:01	RAYLDGTCVE	WLRRYLENGK	ETLQRT	181
HLA-A*11:01	RAYLEGRCVE	WLRRYLENGK	ETLQRT	181
HLA-A*23:01	RAYLEGTCVD	GLRRYLENGK	ETLQRT	181
HLA-A*24:02	RAYLEGTCVD	GLRRYLENGK	ETLQRT	181
HLA-A*25:01	RAYLEGRCVE	WLRRYLENGK	ETLQRT	181
HLA-A*26:01	RAYLEGRCVE	WLRRYLENGK	ETLQRT	181
HLA-A*29:01	RAYLEGTCVE	WLRRYLENGK	ETLQRT	181
HLA-A*30:01	RAYLEGTCVE	WLRRYLENGK	ETLQRT	181
HLA-A*31:01	RAYLEGTCVE	WLRRYLENGK	ETLQRT	181
HLA-A*32:01	RAYLEGTCVE	WLRRYLENGK	ETLQRT	181
HLA-A*33:01	RAYLEGTCVE	WLRRHLENGK	ETLQRT	181
HLA-A*34:01	RAYLEGTCVE	WLRRYLENGK	ETLQRT	181
HLA-A*36:01	RVYLEGTCVE	WLRRYLENGK	ETLQRT	181
HLA-A*43:01	RAYLEGRCVE	WLRRYLENGK	ETLQRT	181
HLA-A*66:01	RAYLEGRCVE	WLRRYLENGK	ETLQRT	181
HLA-A*68:01	RAYLEGTCVE	WLRRYLENGK	ETLQRT	181
HLA-A*69:01	RAYLEGTCVE	WLRRYLENGK	ETLQRT	181
HLA-A*80:01	RAYLEGECVD	GLRRYLENGK	ETLQRT	181
HLA-B*07:02:01:01	RAYLEGECVE	WLRRYLENGK	DKLERA	181
HLA-B*13:01:01:01	RAYLEGECVE	WLRRYLENGK	ETLQRA	181
HLA-B*27:01	RAYLEGECVE	WLRRYLENGK	ETLQRA	181
HLA-B*37:01:01:01	RAYLEGTCVE	WLRRYLENGK	ETLQRA	181
HLA-B*38:01:01:01	RTYLEGTCVE	WLRRYLENGK	ETLQRA	181
HLA-B*40:01:01	RAYLEGECVE	WLRRYLENGK	DKLERA	181
HLA-B*41:01:01:01	RAYLEGTCVE	WLRRYLENGK	DTLERA	181
HLA-B*42:01:01:01	RAYLEGTCVE	WLRRYLENGK	DTLERA	181
HLA-B*45:01:01:01	RAYLEGLCVE	SLRRYLENGK	ETLQRA	181
HLA-B*46:01:01:01	RAYLEGLCVE	WLRRYLENGK	ETLQRA	181
HLA-B*47:01:01:02	RAYLEGECVE	WLRRYLENGK	ETLQRA	181
HLA-B*49:01:01:01	RAYLEGLCVE	WLRRYLENGK	ETLQRA	181
HLA-B*53:01:01:01	RAYLEGLCVE	WLRRYLENGK	ETLQRA	181

		20		40		60		80
HLA-B*54:01:01:01	SHSMRYFYTA	MSRPGRGEPR	FIAVGYVDDT	QFVRFDSDAA	SPRGEPRAPW	V-EQEGPE--	-YWDRNTQIY	KAQAQTDRES
HLA-B*57:01:01:01	SHSMRYFYTA	MSRPGRGEPR	FIAVGYVDDT	QFVRFDSDAA	SPRMAPRAPW	I-EQEGPE--	-YWDGETRNM	KASAQTYREN
HLA-B*58:01:01:01	SHSMRYFYTA	MSRPGRGEPR	FIAVGYVDDT	QFVRFDSDAA	SPRTEPRAPW	I-EQEGPE--	-YWDGETRNM	KASAQTYREN
HLA-B*59:01:01:01	SHSMRYFYTA	MSRPGRGEPR	FIAVGYVDDT	QFVRFDSDAA	SPREEPRAPW	I-EQEGPE--	-YWDRNTQIF	KTNTQTYREN
HLA-B*73:01:01:01	SHSMRYFHTS	VSRPGRGEPR	FITVGYVDDT	QFVRFDSDAA	SPREEPRAPW	I-EQEGPE--	-YWDRNTQIC	KAKAQTDRVG
HLA-B*81:01:01:01	SHSMRYFYTS	VSRPGRGEPR	FISVGYVDDT	QFVRFDSDAA	SPREEPRAPW	I-EQEGPE--	-YWDRNTQIY	KAQAQTDRES
HLA-B*83:01	SHSMRYFYTA	MSRPGRGEPR	FISVGYVDDT	QFVRFDSDAA	SPREEPRAPW	I-EQEGPE--	-YWDRNTQIY	KAQAQTDRES
HLA-C*01:02:01:01	SHSMKYFFTS	VSRPGRGEPR	FISVGYVDDT	QFVRFDSDAA	SPRGEPRAPW	V-EQEGPE--	-YWDRETQKY	KRQAQTDRVS
HLA-C*01:23	SHSMKYFFTS	VSRPGRGEPR	FISVGYVDDT	QFVRFDSDAA	SPRGEPRAPW	V-EQEGPE--	-YWDRETQKY	KRQAQADRVS
HLA-C*02:02:01	SHSMRYFYTA	VSRPSRGEPH	FIAVGYVDDT	QFVRFDSDAA	SPRGEPRAPW	V-EQEGPE--	-YWDRETQKY	KRQAQTDRVN
HLA-C*03:02:01	SHSMRYFYTA	VSRPGRGEPH	FIAVGYVDDT	QFVRFDSDAA	SPRGEPRAPW	V-EQEGPE--	-YWDRETQKY	KRQAQTDRVS
HLA-C*03:274	SHSMRYFYTA	VSRPGRGEPH	FIAVGYVDDT	QFVRFDSDAA	SPRGEPRAPW	V-EQEGPE--	-YWDRETQKY	KRQAQADRES
HLA-C*04:01:01:01	SHSMRYFSTS	VSWPGRGEPR	FIAVGYVDDT	QFVRFDSDAA	SPRGEPREPW	V-EQEGPE--	-YWDRETQKY	KRQAQADRVN
HLA-C*04:77	SHSMRYFSTS	VSWPGRGEPR	FIAVGYVDDT	QFVRFDSDAA	SPRGEPREPW	V-EQEGPE--	-YWDRETQKY	KRQAQADRVN
HLA-C*05:01:01:01	SHSMRYFYTA	VSRPGRGEPR	FIAVGYVDDT	QFVQFDSDAA	SPRGEPRAPW	V-EQEGPE--	-YWDRETQKY	KRQAQTDRVN
HLA-C*06:02:01:01	SHSMRYFDTA	VSRPGRGEPR	FISVGYVDDT	QFVRFDSDAA	SPRGEPRAPW	V-EQEGPE--	-YWDRETQKY	KRQAQADRVN
HLA-C*07:01:01:01	SHSMRYFDTA	VSRPGRGEPR	FISVGYVDDT	QFVRFDSDAA	SPRGEPRAPW	V-EQEGPE--	-YWDRETQNY	KRQAQADRVS
HLA-C*07:38:02	SHSMRYFDTA	VSRPGRGEPR	FISVGYVDDT	QFVRFDSDAA	SPRGEPRAPW	V-EQEGPE--	-YWDRETQKY	KRQAQADRVS
HLA-C*08:01:01:01	SHSMRYFYTA	VSRPGRGEPR	FIAVGYVDDT	QFVQFDSDAA	SPRGEPRAPW	V-EQEGPE--	-YWDRETQKY	KRQAQTDRVS
HLA-C*12:02:01	SHSMRYFYTA	VSRPGRGEPR	FIAVGYVDDT	QFVRFDSDAA	SPRGEPRAPW	V-EQEGPE--	-YWDRETQKY	KRQAQADRVS
HLA-C*14:02:01:01	SHSMRYFSTS	VSRPGRGEPR	FIAVGYVDDT	QFVRFDSDAA	SPRGEPRAPW	V-EQEGPE--	-YWDRETQKY	KRQAQTDRVS
HLA-C*15:02:01:01	SHSMRYFYTA	VSRPGRGEPH	FIAVGYVDDT	QFVRFDSDAA	SPRGEPRAPW	V-EQEGPE--	-YWDRETQNY	KRQAQTDRVN
HLA-C*15:36	SHSMRYFSTS	VSWPGRGEPH	FIAVGYVDDT	QFVRFDSDAA	SPRGEPRAPW	V-EQEGPE--	-YWDRETQNY	KRQAQTDRVN
HLA-C*16:01:01:01	SHSMRYFYTA	VSRPGRGEPR	FIAVGYVDDT	QFVRFDSDAA	SPRGEPRAPW	V-EQEGPE--	-YWDRETQKY	KRQAQTDRVS
HLA-C*17:01:01:02	SHSMRYFYTA	VSRPGRGEPR	FIAVGYVDDT	QFVRFDSDAA	SPRGEPRAPW	V-EQEGPE--	-YWDRETQKY	KRQAQADRVN
HLA-C*17:06	SHSMRYFYTA	VSRPGRGEPR	FIAVGYVDDT	QFVRFDSDAA	SPRGEPRAPW	V-EQEGPE--	-YWDRETQKY	KRQAQADRVN
HLA-C*18:01:01:01	SHSMRYFDTA	VSRPGRGEPR	FISVGYVDDT	QFVRFDSDAA	SPRGEPRAPW	V-EQEGPE--	-YWDRETQKY	KRQAQADRVN
Ovar-N*01:01	PHSLRYVYTG	VSRPGLGEPR	FIAVGYVDDT	QFVRFDSDAP	DPREEPRARW	V-EQEGPE--	-YWDRNTRIY	KDTAQIFRAN
Ovar-N*05:01	PHSMRYFSTA	VSRPGRGEPR	YLEVGYVDDT	QFVRFDSDAP	DPKMEQRAPW	M-EQVGPE--	-YWDRETRRA	KGNGQSFRVG
Ovar-N*06:01	SHSLRYVYTA	VSRPGRGEPR	FIAVGYVDDT	QFVRFDSDAA	DPRMEPRARW	V-EQEGPE--	-YWDQETQGT	KDAAQTFRAN
Ovar-N*07:01	PHSLRYFLTA	VSRPGRGEPR	FIAVGYVDDT	QFVRFDSDAA	DPRMEPRARW	V-EQEGPE--	-YWDQETRSA	KGHAQTFRVN
Ovar-N*08:01	PHSLRYFLTA	VSRPGRGEPR	FIAVGYVDDT	QFVRFDSDAA	DPRMEPRARW	V-EQEGPE--	-YWDRETRKA	NDDAQTFRVN
Ovar-N*11:01	PHSMRYVYTG	VSRPGLGEPR	FIAVGYVDDT	QFVRFDSDAP	DPRMEPRARW	V-EQEGPE--	-YWDRETRNM	KDATQSFRVG
Ovar-N*12:01	PHSLRYFLTA	VSRPGRGEPR	FIAVGYVDDT	QFVRFDSDAA	DPRMEPRARW	V-EQEGPE--	-YWDRETRKA	NDDAQTFRVN
Ovar-N*13:01	SHSMRYFSTA	VSRPGLGEPR	YLEVGYVDDT	QFVRFDSDAP	DPRMEPRAPW	M-EQVGPE--	-YWDRNTRNA	KGNAQTFRVN
Ovar-N*14:01	PHSLRYFYTG	VSRPGRGEPR	FIIVGYVDDT	QFVRFDSDAP	DPRDEPRAPW	I-EQEGPE--	-YWDQETRKS	KDAAQTFRVD
Ovar-N*15:01	PHSMRYFSTA	VSRPGRGEPR	YLEVGYVDDT	QFVRFDSDAP	DPKMEQRAPW	M-EQVGPE--	-YWDRNTRNA	KGNAQTFRVG
Ovar-N*16:01	PHSMRYFSTA	VSRPGLGEPR	YLEVGYVDDT	QFVRFDSDAP	DPKMEPRAPW	M-EQEGPE--	-YWDRETRRA	KGNAQTFRVG
Ovar-N*18:01	PHSMRYFSTA	VSRPGRGEPR	YLEVGYVDDT	QFVRFDSDAP	DPKMEQRAPW	M-EQVGPE--	-YWDRNTRNA	KGTAQTFRVD
Ovar-N*19:01	PHSLRYFLTA	VSRPGLGEPR	FIAVGYVDDT	QFVRFDSDAP	DPRMEPRAPW	M-EQEGPE--	-YWDLNTRNA	KGHAQTFRVS
Ovar-N*20:01	SHSMRYFSTA	VSRPGLGEPR	YLEVGYVDDT	QFVRFDSDAP	DPRMEPRARW	V-EQEGPE--	-YWDLNTRNA	KGTALTFRVS
Ovar-N*21:01	SHSMRYFSTA	VSRPGLGEPR	YLEVGYVDDT	QFVRFDSDAP	DPRMEPRAPW	M-EQVGPE--	-YWDRNTRNA	KGNAQTFRVN
Ovar-N*22:01	PHSLRYFLTA	VSRPGRGEPR	FIAVGYVDDT	QFVRFDSDAA	DPRMEPRARW	V-EQEGPE--	-YWDRETRKA	NDDAQTFRVN
Ovar-N*24:01	SHSLRYFLTA	VSRPGRGEPR	YLEVGYVDDM	QFVRFDSDAP	DPKMEPRARW	V-EQEGPE--	-YWDQETRRA	KDTAQTFRVS
Ovar-N*26:01	PHSLRYFYTA	VSRPGRGEPR	FIAVGYVDDT	QFVRFDSDAP	DPREEPRARW	V-EQEGPE--	-YWDRNTRIY	KDAAQIFRVS
Ovar-N*27:01	PHSMRYFSTA	VSRPGRGEPR	YLEVGYVDDT	QFVRFDSDAP	DPRMEQRAPW	M-EQVGPE--	-YWDQETRRA	KDTAQTFRVN
Ovar-N*50:01	SHSLRYVYTA	VSRPGRGEPR	FIAVGYVDDT	QFVRFDSDAP	DPRMEPRARW	V-EQEGPE--	-YWDQETRIY	KDAAQNFRAN
Patr-A*01:01:01:01	SHSMRYFFTS	VSRPGRGEPR	FIAVGYVDDT	QFVRFDSDAA	SQRMEPRAPW	I-EQEGPE--	-YWDQETRSA	KAHSQTDRVD
Patr-A*02:01:01:01	SHSMRYFFTS	VSRPGRGEPR	FIAVGYVDDT	QFVRFDSDAA	SQRMEPRAPW	I-EQEGPE--	-YWDEETRSA	KAHSQTDRVD
Patr-A*03:01:01:01	SHSMRYFYTS	VSRPGRGEPR	FIAVGYVDDT	QFVRFDSDAA	SQRMEPRAPW	I-EQEGPE--	-YWDQETRNM	KASAQTDRVD
Patr-A*05:01:01:01	SHSMRYFSTS	VSRPGRGEPR	FIAVGYVDDT	QFVRFDSDAA	SQRMEPRAPW	I-EQEGPE--	-YWDEETRSV	KAFAQTDRVD
Patr-A*06:01:01:01	SHSMRYFSTS	VSRPGRGEPR	FIAVGYVDDT	QFVRFDSDAA	SQRMEPRAPW	I-EQEGPE--	-YWDQETRSV	KASAQTDRAN
Patr-A*07:01:01:01	SHSMRYFSTS	VSRPGRGEPR	FIAVGYVDDT	QFVRFDSDAA	SQRMEPRAPW	I-EQEGPE--	-YWDRETRSV	KAGAQIYRAN
Patr-A*08:01:01:01	SHSMRYFSTS	VSRPGRGEPR	FIAVGYVDNT	QFVRFDSDAA	SQRMEPRAPW	I-EQEGPE--	-YWDQETRSV	KAGAQIYRAN
Patr-A*09:01:01:01	SHSMRYFSTS	VSRPGRGEPR	FIAVGYVDDT	QFVRFDSDAA	SQRMEPRAPW	I-EQEGPE--	-YWDEETRSV	KASAQTDRVD
Patr-A*10:01:01:01	SHSMRYFSTS	VSRPGRGEPR	FIAVGYVDNT	QFVRFDSDAA	SQRMEPRAPW	I-EQEGPE--	-YWDQETRNM	KAFAQIYRAN
Patr-A*11:01:01:01	SHSMRYFYTS	VSRPGRGEPR	FIAVGYVDDT	QFVRFDSDAA	SQRMEPRAPW	I-EQEGPE--	-YWDQETRNM	KASAQTDRAN
Patr-A*14:01:01:01	SHSMRYFSTS	VSRPGRGEPR	FIAVGYVDDT	QFVRFDSDAA	SQRMEPRAPW	I-EQEGPE--	-YWDEETRSV	KAFAQTDRAN
Patr-A*16:01:01:01	SHSMRYFYTS	VSRPGRGEPR	FIAVGYVDDT	QFVRFDSDAA	SQRMEPRAPW	I-EQEGPE--	-YWDEETRSA	KAHSQTDRVD
Patr-A*17:01:01:01	SHSMRYFYTS	VSRPGRGEPR	FIAVGYVDDT	QFVRFDSDAA	SQRMEPRAPW	I-EQEGPE--	-YWDEETRST	KASAQTNRVD
Patr-A*18:01:01:01	SHSMRYFSTS	VSRPGRGEPR	FIAVGYVDDT	QFVRFDSDAA	SQRMEPRAPW	I-EQEGPE--	-YWDEETRSV	KAFAQTDRVD
Patr-A*21:01	SHSMRYFYTS	VSRPGRGEPR	FIAVGYVDDT	QFVRFDSDAA	SQRMEPRAPW	I-EQEGPE--	-YWDQETRNV	KAQAQTDRVD
Patr-A*22:01:01:01	SHSMRYFSTS	VSRPGRGEPR	FIAVGYVDDT	QFVRFDSDAA	SQRMEPRAPW	I-EQEGPE--	-YWDRETRSV	KASAQTDRAN
Patr-A*24:01	SHSMRYFSTS	VSRPGRGEPR	FIAVGYVDDT	QFVRFDSDAA	SQRMEPRAPW	I-EQEGPE--	-YWDRETRSV	KAGAQTDRAN
Patr-A*25:01:01:01	SHSMRYFSTS	VSRPGRGEPR	FIAVGYVDNT	QFVRFDSDAA	SQRMEPRAPW	I-EQEGPE--	-YWDRETRSV	KAGAQIYRAN
Patr-A*26:01:01:01	SHSMRYFSTS	VSRPGRGEPR	FIAVGYVDDT	QFVRFDSDAA	SQRMEPRAPW	I-EQEGPE--	-YWDEETRSV	KASAQIYRAN
Patr-A*27:01:01:01	SHSMRYFFTS	VSRPGRGEPR	FIAVGYVDDT	QFVRFDSDAA	SQRMEPRAPW	I-EQEGPE--	-YWDEETRSV	KAFAQTDRVD
Patr-B*02:01	SHSMKYFYTA	VSRPGRGEPR	FISVGYVDDT	QFVWFDSDAA	SPREEPRAPW	I-EQEGPE--	-YWDRETQIS	KTNAQTYRES
Patr-B*05:01:01:01	SHSMKYFYTA	VSRPGRGEPR	FISVGYVDDT	QFVWFDSDAA	SPREEPRAPW	I-EQEGPE--	-YWDRETQIS	KTNAQTYRES
Patr-B*07:01	SHSMKYFLTS	VSRPGRGEPR	FITVGYVDDT	QFVRFDSDAA	SPRMEPRAPW	I-EQEGPE--	-YWDRETRNV	KASAQTYREN
Patr-B*08:01	SHSMRYFYTA	MSRPGGGEPR	FIAVGYVDDT	QFVRFDSDAA	IPRMEPRAPW	I-EQEGPE--	-YWDRETQIS	KTNAQTYRVG
Patr-B*11:01	SHSMRYFYTS	VSRPGRGEPR	FISVGYVDDT	QFVRFDSDAA	SPREEPRAPW	M-EQEGPE--	-YWDRNTQIY	KAQAQTDRVG
Patr-B*12:02	SHSMRYFYTS	VSRPGRGEPR	FISVGYVDDT	QFVRFDSDAA	SPREEPRAPW	M-EQEGPE--	-YWDRNTQIC	KAQAQTDRGS
Patr-B*13:01:01:01	SHSMRYFYTS	VSRPGRGEPR	FISVGYVDDT	QFVRFDSDAA	SPREEPRAPW	M-EQEGPE--	-YWDRNTQIY	KAQAQTDRVS
Patr-B*14:01:01:01	SQSMRYFYTA	MSRPGRGEPR	FIAVGYVDDT	QFVRFDSDAA	IPRMEPRAPW	I-EQEGPE--	-YWDRNTQGI	KTQAQTDREN
Patr-B*17:01	SHSMKYFYTS	VSRPGRGEPR	FISVGYVDDT	QFVRFDSDAA	SPRVEPRAPW	M-EQEGPE--	-YCDRETQIF	KTNTQTDRVS
Patr-B*18:01:01:01	SHSMKYFFTS	VSRPGRGEPR	FITVGYVDDT	QFVRFDSDAA	SPRMEPRAPW	I-EQEGPE--	-YWDRETRNV	KASAQTDREN
Patr-B*23:01:01	SHSMKYFYTA	VSRPGRGEPR	FISVGYVDDT	QFVWFDSDAA	SPREEPRAPW	I-EQEGPE--	-YWDRETQIS	KTNTQTYREN
Patr-B*24:01:01:01	SHSMKYFYTA	VSRPGRGEPR	FITVGYVDDT	QFVWFDSDAA	SPRKEPRTPW	I-EQEGPE--	-YWDRETQIS	KTNAQTDREN
Patr-B*25:01	SHSMKYFLTS	VSRPGRGEPR	FITVGYVDDT	QFVRFDSDAA	SPRMEPRAPW	I-EQEGPE--	-YWDRETRNM	KASAQTYREN
Patr-B*26:01	SHSMRYFYTS	VSRPGRGEPR	FITVGYVDDT	QFVRFDSDAA	SPRMEPRAPW	I-EQEGPE--	-YWDRETRNV	KASALTDREN
Patr-B*29:01:01:01	SHSMRYFYTA	MSRPGRGEPR	FIAVGYVDDT	QFVRFDSDAA	IPRMEPRAPW	I-EQEGPE--	-YWDGNTQGV	KTQAQTDRES
Patr-B*30:01	SHSMKYFYTA	VSRPGRGEPR	FITVGYVDDT	QFVWFDSDAA	SPRKEPRTPW	I-EQEGPE--	-YWDRETQIS	KTNAQTYREN
Patr-B*35:01	SHSMRYFYIA	MSRPGRGEPR	FIAVGYVDDT	QFVRFDSDAA	IPRMEPRAPW	I-EQEGPE--	-YWDRNTQIY	KAQAQTDRES
Patr-B*36:01:01:01	SHSMRYFYTA	MSRPGRGEPR	FIAVGYVDDT	QFVRFDSDAA	IPRMEPRAPW	I-EQEGPE--	-YWDRNTQGV	KAQAQTDREN
Patr-B*37:01	SHSMRYFYTA	MSRPGRGEPR	FIAVGYVDDT	QFVRFDSDAA	IPRMEPRAPW	I-EQEGPE--	-YWDRNTQGV	KAQAQTDREN
Patr-B*39:01	SHSMKYFYTA	VSRPGRGEPR	FITVGYVDDT	QFVWFDSDAA	SPRKEPRTPW	I-EQEGPE--	-YWDRETQIS	KTNTQTYRES
Patr-C*02:01	SHSLRYFDTA	VSRPGRREPR	FISVGYVDDT	QFVRFDSDAA	SPRGEPRAPW	V-EQEGPE--	-YWDRETQKY	KRQAQADRVS
Patr-C*02:03:01	SHSLRYFDTA	VSRPGRREPR	FISVGYVDDT	QFVRFDSDAA	SPRGEPRAPW	V-EQEGPE--	-YWDRETQKY	KRQAQADRVN
Patr-C*02:04:01:01	SHSLRYFDTA	VSRPGRREPR	FISVGYVDDT	QFVRFDSDAA	SPRGEPRAPW	V-EQEGPE--	-YWDRETQKY	KRQAQADRVS
Patr-C*02:05:01:01	SHSLRYFDTA	VSRPGRREPR	FISVGYVDDT	QFVRFDSDAA	SPRGEPRAPW	V-EQEGPE--	-YWDRETQKY	KRQAQADRVS

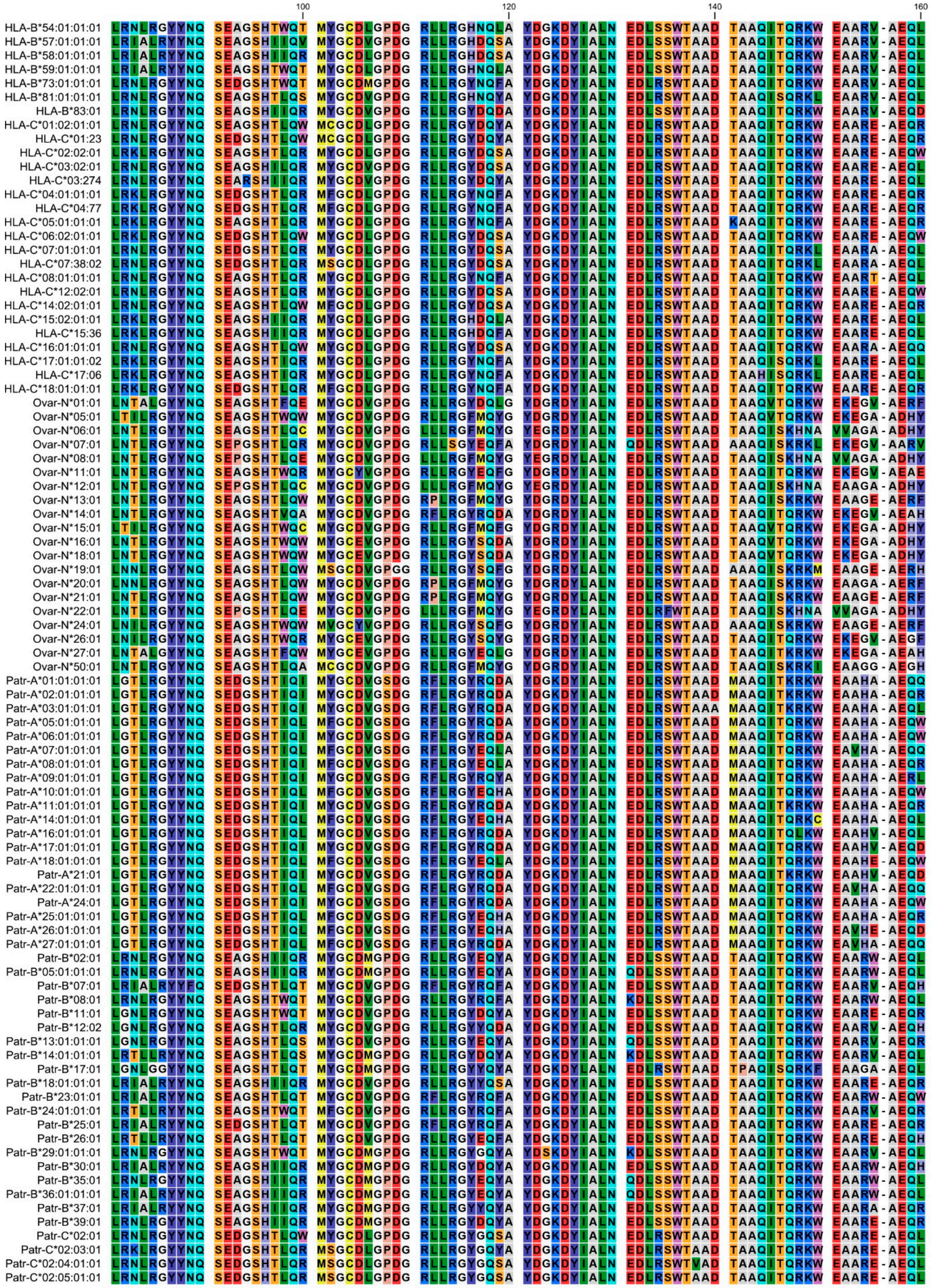
100
120
140
160
HLA-B*54:01:01:01
HLA-B*57:01:01:01
HLA-B*58:01:01:01
HLA-B*59:01:01:01
HLA-B*73:01:01:01
HLA-B*81:01:01:01
HLA-B*83:01
HLA-C*01:02:01:01
HLA-C*01:23
HLA-C*02:02:01
HLA-C*03:02:01
HLA-C*03:274
HLA-C*04:01:01:01
HLA-C*04:77
HLA-C*05:01:01:01
HLA-C*06:02:01:01
HLA-C*07:01:01:01
HLA-C*07:38:02
HLA-C*08:01:01:01
HLA-C*12:02:01
HLA-C*14:02:01:01
HLA-C*15:02:01:01
HLA-C*15:36
HLA-C*16:01:01:01
HLA-C*17:01:01:02
HLA-C*17:06
HLA-C*18:01:01:01
Ovar-N*01:01
Ovar-N*05:01
Ovar-N*06:01
Ovar-N*07:01
Ovar-N*08:01
Ovar-N*11:01
Ovar-N*12:01
Ovar-N*13:01
Ovar-N*14:01
Ovar-N*15:01
Ovar-N*16:01
Ovar-N*18:01
Ovar-N*19:01
Ovar-N*20:01
Ovar-N*21:01
Ovar-N*22:01
Ovar-N*24:01
Ovar-N*26:01
Ovar-N*27:01
Ovar-N*50:01
Patr-A*01:01:01:01
Patr-A*02:01:01:01
Patr-A*03:01:01:01
Patr-A*05:01:01:01
Patr-A*06:01:01:01
Patr-A*07:01:01:01
Patr-A*08:01:01:01
Patr-A*09:01:01:01
Patr-A*10:01:01:01
Patr-A*11:01:01:01
Patr-A*14:01:01:01
Patr-A*16:01:01:01
Patr-A*17:01:01:01
Patr-A*18:01:01:01
Patr-A*21:01
Patr-A*22:01:01:01
Patr-A*24:01
Patr-A*25:01:01:01
Patr-A*26:01:01:01
Patr-A*27:01:01:01
Patr-B*02:01
Patr-B*05:01:01:01
Patr-B*07:01
Patr-B*08:01
Patr-B*11:01
Patr-B*12:02
Patr-B*13:01:01:01
Patr-B*14:01:01:01
Patr-B*17:01
Patr-B*18:01:01:01
Patr-B*23:01:01:01
Patr-B*24:01:01:01
Patr-B*25:01
Patr-B*26:01
Patr-B*29:01:01:01
Patr-B*30:01
Patr-B*35:01
Patr-B*36:01:01:01
Patr-B*37:01
Patr-B*39:01
Patr-C*02:01
Patr-C*02:03:01
Patr-C*02:04:01:01
Patr-C*02:05:01:01

		180		
HLA-B*54:01:01:01	RAYLEGTCVE	WLRRYLENGK	ETLQRA	181
HLA-B*57:01:01:01	RAYLEGLCVE	WLRRYLENGK	ETLQRA	181
HLA-B*58:01:01:01	RAYLEGLCVE	WLRRYLENGK	ETLQRA	181
HLA-B*59:01:01:01	RAYLEGTCVE	WLRRYLENGK	ETLQRA	181
HLA-B*73:01:01:01	RAYLEGECVE	WLRRHLENGK	ETLQRA	181
HLA-B*81:01:01:01	RAYLEGECVE	WLRRYLENGK	DKLERA	181
HLA-B*83:01	RAYLEGLCVE	SLRRYLENGK	ETLQRA	181
HLA-C*01:02:01:01	RAYLEGTCVE	WLRRYLENGK	ETLQRA	181
HLA-C*01:23	RAYLEGTCVE	WLRRYLENGK	ETLQRA	181
HLA-C*02:02:01	RAYLEGECVE	WLRRYLENGK	ETLQRA	181
HLA-C*03:02:01	RAYLEGLCVE	WLRRYLKNGK	ETLQRA	181
HLA-C*03:274	RAYLEGLCVE	WLRRYLKNGK	ETLQRA	181
HLA-C*04:01:01:01	RAYLEGTCVE	WLRRYLENGK	ETLQRA	181
HLA-C*04:77	RAYLEGECVE	WLRRYLENGK	DKLERA	181
HLA-C*05:01:01:01	RAYLEGTCVE	WLRRYLENGK	KTLQRA	181
HLA-C*06:02:01:01	RAYLEGTCVE	WLRRYLENGK	ETLQRA	181
HLA-C*07:01:01:01	RAYLEGTCVE	WLRRYLENGK	ETLQRA	181
HLA-C*07:38:02	RAYLEGTCVE	WLRRYLENGK	DKLERA	181
HLA-C*08:01:01:01	RAYLEGTCVE	WLRRYLENGK	KTLQRA	181
HLA-C*12:02:01	RAYLEGTCVE	WLRRYLENGK	ETLQRA	181
HLA-C*14:02:01:01	RAYLEGTCVE	WLRRYLENGK	ETLQRA	181
HLA-C*15:02:01:01	RAYLEGTCVE	WLRRYLENGK	ETLQRA	181
HLA-C*15:36	RAYLEGTCVE	WLRRYLENGK	ETLQRA	181
HLA-C*16:01:01:01	RAYLEGTCVE	WLRRYLENGK	ETLQRA	181
HLA-C*17:01:01:02	RAYLEGECVE	WLRGYLENGK	ETLQRA	181
HLA-C*17:06	RAYLEGECVE	WLRGYLENGK	ETLQRA	181
HLA-C*18:01:01:01	RAYLEGTCVE	WLRRYLENGK	ETLQRA	181
Ovar-N*01:01	RNYVEGRCVE	GLRRYLEIGK	DTLLRA	181
Ovar-N*05:01	RNYLEGTCVE	WLRRYLEIGK	DTLQRA	181
Ovar-N*06:01	RNYLEGECVE	WLRRHLETGK	DTLLRA	181
Ovar-N*07:01	RIYLEGRCVE	GLRRYLETGK	DTLQRA	181
Ovar-N*08:01	RNYLVGECVE	WLLRHLETGK	DTLLRA	181
Ovar-N*11:01	RNYLEGRCVE	WLRRYLETGK	DTLLRA	181
Ovar-N*12:01	RNYLVGECVE	WLLRHLETGK	DTLLRA	181
Ovar-N*13:01	RNYLEGTCVE	WLRRYLETGK	DTLQRA	181
Ovar-N*14:01	RNYLEGECVE	WLRRYLENGK	DTLLRA	181
Ovar-N*15:01	RNYVEGTCVE	WLRRYLEIGK	DTLQRA	181
Ovar-N*16:01	RNYLEGTCVE	WLRRHLENGK	DTLLRA	181
Ovar-N*18:01	RNYLEGTCVE	WLRRHLENGK	DTLLRA	181
Ovar-N*19:01	RNYVEGTCVE	WLRRYLENGK	DTLLRA	181
Ovar-N*20:01	RNYLEGTCVE	WLRRYLETGK	DTLQRA	181
Ovar-N*21:01	RNYLEGTCVE	WLRRYLETGK	DTLQRA	181
Ovar-N*22:01	RNYLVGECVE	WLLRHLETGK	DTLLRA	181
Ovar-N*24:01	RNYLEGRCVE	GLRRYLEIGK	DTLLRA	181
Ovar-N*26:01	RNYVEGLCVE	GLRRYLEIGK	DTLLRA	181
Ovar-N*27:01	RNYLEGECVE	WLHRHLETGK	DTLLRA	181
Ovar-N*50:01	RNYFEGLCVE	ELRRYLETGK	DTLLRA	181
Patr-A*01:01:01:01	RAYLEGTCVE	WLRRYLENGK	ETLQRT	181
Patr-A*02:01:01:01	RAYLEGTCVE	WLRRYLENGK	ETLQRT	181
Patr-A*03:01:01:01	RAYLEGRCVE	WLRRYLENGK	ETLQRT	181
Patr-A*05:01:01:01	RAYLEGTCVD	GLRRYLENGK	ETLQRT	181
Patr-A*06:01:01:01	RAYLEGTCVD	GLRRYLENGK	ETLQRT	181
Patr-A*07:01:01:01	RAYLEGRCVE	SLRRYLENGK	ETLQRT	181
Patr-A*08:01:01:01	RAYLEGTCVE	WLRRYLENGK	ETLQRT	181
Patr-A*09:01:01:01	RAYLEGTCVE	WLRRYLENGK	ETLQRT	181
Patr-A*10:01:01:01	RAYLEGRCVD	GLRRYLENGK	ETLQRT	181
Patr-A*11:01:01:01	RAYLEGTCVD	GLRRYLENGK	ETLQRT	181
Patr-A*14:01:01:01	RAYLEGTCVE	WLRRYLENGK	ETLQRT	181
Patr-A*16:01:01:01	RAYLEGLCVD	GLRRYLENGK	ETLQRT	181
Patr-A*17:01:01:01	RAYLEGTCVE	WLRRYLENGK	ETLQRT	181
Patr-A*18:01:01:01	RAYLEGRCVE	WLRRYLENGK	ETLQRT	181
Patr-A*21:01	RAYLEGTCVE	WLRRYLENGK	ETLQRA	181
Patr-A*22:01:01:01	RAYLEGRCVE	SLRRYLENGK	ETLQRT	181
Patr-A*24:01	RAYLEGTCVD	GLRRYLENGK	ETLQRT	181
Patr-A*25:01:01:01	RAYLEGLCVD	GLRRYLENGK	ETLQRT	181
Patr-A*26:01:01:01	RAYLEGTCVE	WLRRYLENGK	ETLQRT	181
Patr-A*27:01:01:01	RAYLEGRCME	WLRRYLENGK	ETLQRT	181
Patr-B*02:01	RAYLEGTCVE	WLRRYLENGK	ETLQRA	181
Patr-B*05:01:01:01	RAYLEGTCVE	WLRRYLENGK	ETLQRA	181
Patr-B*07:01	RAYLEGLCVE	WLRRYLENGK	ETLQRA	181
Patr-B*08:01	RAYLEGTCVE	WLRRYLENGK	ETLQRA	181
Patr-B*11:01	RAYLEGLCVE	WLRRYLENGK	ETLQRA	181
Patr-B*12:02	RAYLEGLCVE	WLRRYLENGK	ETLQRA	181
Patr-B*13:01:01:01	RAYLEGTCVE	WLRRYLENGK	ETLQRA	181
Patr-B*14:01:01:01	RAYLEGTCVE	WLRRYLENGK	ETLQRA	181
Patr-B*17:01	RAYLEGTCVE	WLRRYLENGK	EMLQRA	181
Patr-B*18:01:01:01	RAYLEGTCVE	WLRRYLENGK	ETLQRA	181
Patr-B*23:01:01	RAYLEGTCVE	WLRRYLENGK	ETLQRA	181
Patr-B*24:01:01:01	RAYLEGTCVE	WLRRYLENGK	ETLQRA	181
Patr-B*25:01	RAYLEGLCVE	WLRRYLENGK	ETLQRA	181
Patr-B*26:01	RAYLEGLCVD	GLRRYLENGK	ETLQRA	181
Patr-B*29:01:01:01	RAYLEGTCVE	WLRRYLENGK	ETLQRA	181
Patr-B*30:01	RAYLEGECVE	WLRRYLENGK	ETLQRA	181
Patr-B*35:01	RAYLEGTCVE	WLRRYLENGK	ETLQRA	181
Patr-B*36:01:01:01	RAYLEGTCVE	WLRRYLENGK	ETLQRA	181
Patr-B*37:01	RAYLEGTCVE	WLRRYLENGK	ETLQRA	181
Patr-B*39:01	RAYLEGTCVE	WLRRYLENGK	ETLQRA	181
Patr-C*02:01	RAYLEGKRVE	SCRRYLENGK	ETLQRT	181
Patr-C*02:03:01	RAYLEGKCVE	WLRRYLENGK	ETLQRT	181
Patr-C*02:04:01:01	RAYLEGKCVE	WLRRYLENGK	ETLQRT	181
Patr-C*02:05:01:01	RAYLEGKCVE	WLRRYLENGK	ETLQRT	181

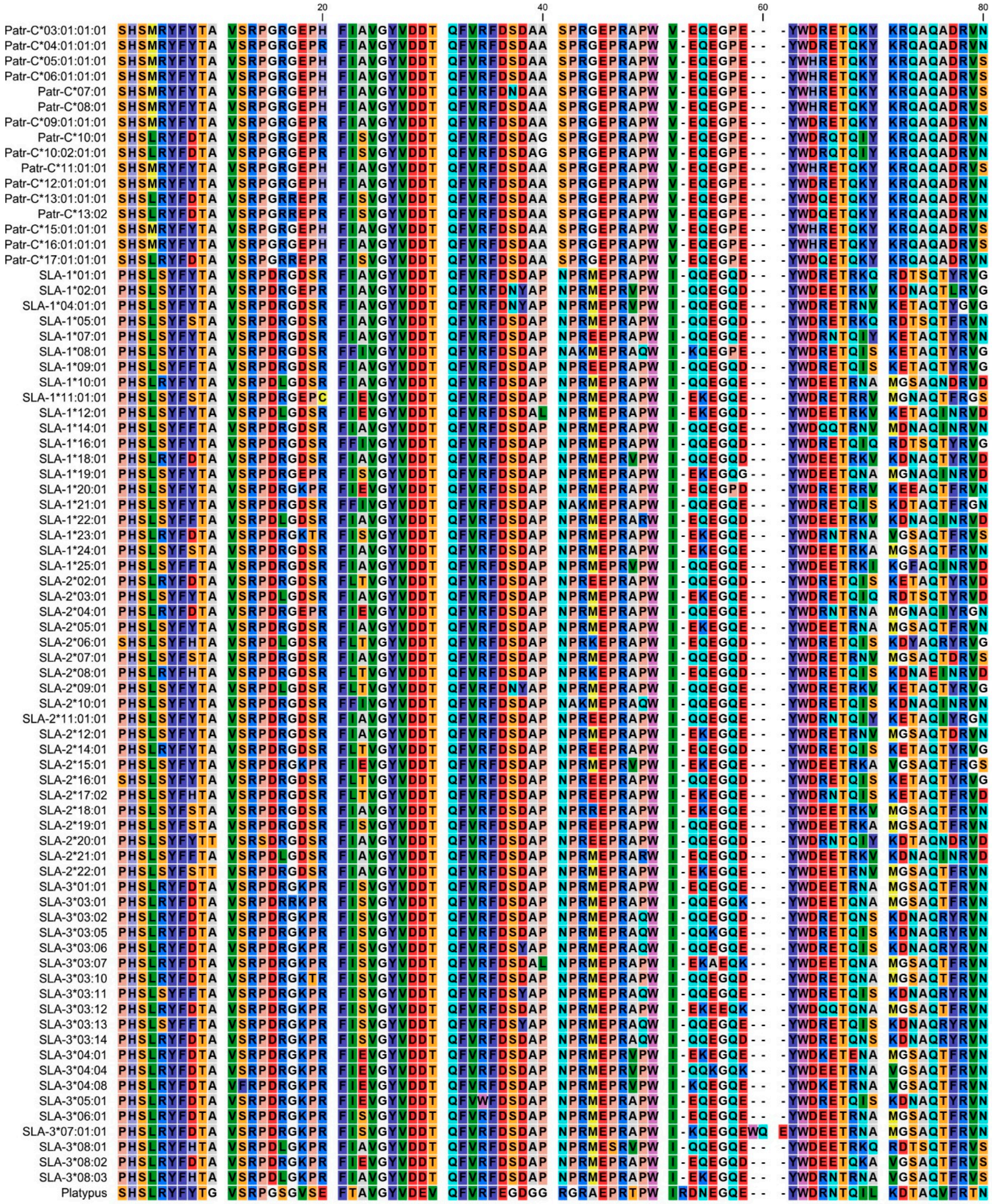
20
40
60
80
Patr-C*03:01:01:01
Patr-C*04:01:01:01
Patr-C*05:01:01:01
Patr-C*06:01:01:01
Patr-C*07:01
Patr-C*08:01
Patr-C*09:01:01:01
Patr-C*10:01
Patr-C*10:02:01:01
Patr-C*11:01:01
Patr-C*12:01:01:01
Patr-C*13:01:01:01
Patr-C*13:02
Patr-C*15:01:01:01
Patr-C*16:01:01:01
Patr-C*17:01:01:01
SLA-1*01:01
SLA-1*02:01
SLA-1*04:01:01
SLA-1*05:01
SLA-1*07:01
SLA-1*08:01
SLA-1*09:01
SLA-1*10:01
SLA-1*11:01:01
SLA-1*12:01
SLA-1*14:01
SLA-1*16:01
SLA-1*18:01
SLA-1*19:01
SLA-1*20:01
SLA-1*21:01
SLA-1*22:01
SLA-1*23:01
SLA-1*24:01
SLA-1*25:01
SLA-2*02:01
SLA-2*03:01
SLA-2*04:01
SLA-2*05:01
SLA-2*06:01
SLA-2*07:01
SLA-2*08:01
SLA-2*09:01
SLA-2*10:01
SLA-2*11:01:01
SLA-2*12:01
SLA-2*14:01
SLA-2*15:01
SLA-2*16:01
SLA-2*17:02
SLA-2*18:01
SLA-2*19:01
SLA-2*20:01
SLA-2*21:01
SLA-2*22:01
SLA-3*01:01
SLA-3*03:01
SLA-3*03:02
SLA-3*03:05
SLA-3*03:06
SLA-3*03:07
SLA-3*03:10
SLA-3*03:11
SLA-3*03:12
SLA-3*03:13
SLA-3*03:14
SLA-3*04:01
SLA-3*04:04
SLA-3*04:08
SLA-3*05:01
SLA-3*06:01
SLA-3*07:01:01
SLA-3*08:01
SLA-3*08:02
SLA-3*08:03
Platypus

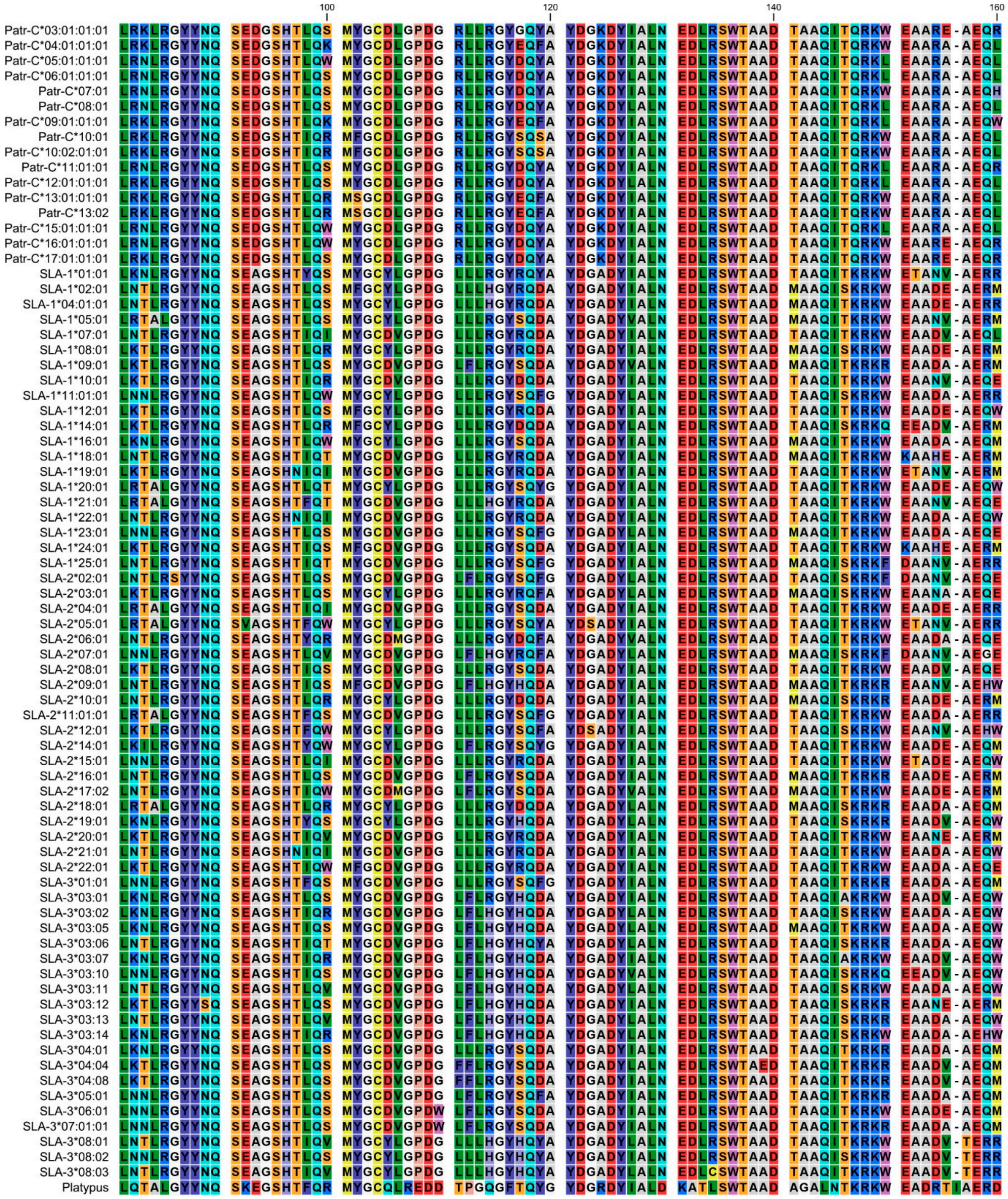
100
120
140
160
Patr-C*03:01:01:01
Patr-C*04:01:01:01
Patr-C*05:01:01:01
Patr-C*06:01:01:01
Patr-C*07:01
Patr-C*08:01
Patr-C*09:01:01:01
Patr-C*10:01
Patr-C*10:02:01:01
Patr-C*11:01:01
Patr-C*12:01:01:01
Patr-C*13:01:01:01
Patr-C*13:02
Patr-C*15:01:01:01
Patr-C*16:01:01:01
Patr-C*17:01:01:01
SLA-1*01:01
SLA-1*02:01
SLA-1*04:01:01
SLA-1*05:01
SLA-1*07:01
SLA-1*08:01
SLA-1*09:01
SLA-1*10:01
SLA-1*11:01:01
SLA-1*12:01
SLA-1*14:01
SLA-1*16:01
SLA-1*18:01
SLA-1*19:01
SLA-1*20:01
SLA-1*21:01
SLA-1*22:01
SLA-1*23:01
SLA-1*24:01
SLA-1*25:01
SLA-2*02:01
SLA-2*03:01
SLA-2*04:01
SLA-2*05:01
SLA-2*06:01
SLA-2*07:01
SLA-2*08:01
SLA-2*09:01
SLA-2*10:01
SLA-2*11:01:01
SLA-2*12:01
SLA-2*14:01
SLA-2*15:01
SLA-2*16:01
SLA-2*17:02
SLA-2*18:01
SLA-2*19:01
SLA-2*20:01
SLA-2*21:01
SLA-2*22:01
SLA-3*01:01
SLA-3*03:01
SLA-3*03:02
SLA-3*03:05
SLA-3*03:06
SLA-3*03:07
SLA-3*03:10
SLA-3*03:11
SLA-3*03:12
SLA-3*03:13
SLA-3*03:14
SLA-3*04:01
SLA-3*04:04
SLA-3*04:08
SLA-3*05:01
SLA-3*06:01
SLA-3*07:01:01
SLA-3*08:01
SLA-3*08:02
SLA-3*08:03
Platypus

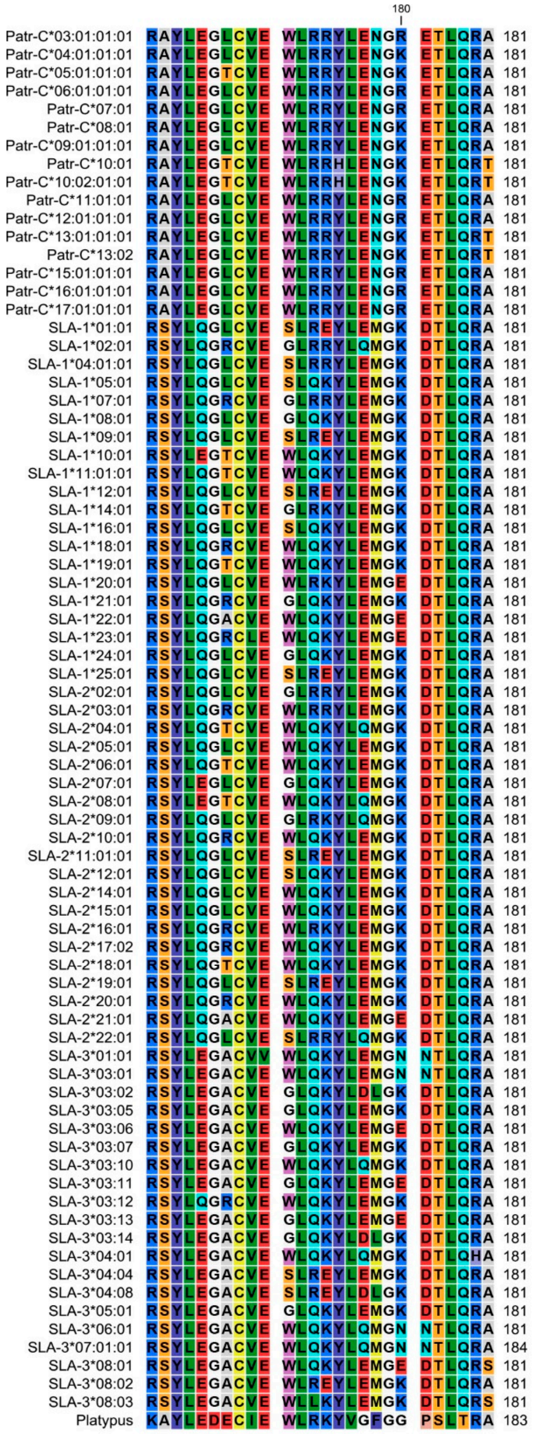

Supplementary Figure S1. Alignment of exons 2 and 3 sequences (546bp) of 238 representative MHC class I alleles of 20 different allelic groups for each gene from pigs, cattle, chimpanzees, and humans, together with a platypus sequence.

Supplementary Table S1. The number of annotated alleles for each MHC class I gene of pigs, cattle, sheep, chimpanzees, and humans available in IPD

	Pig			Cattle			Sheep	Chimpanzee			Human		
	SLA-1	SLA-2	SLA-3	BoLA-1	BoLA-2	BoLA-3	Ovar-N	Patr-A	Patr-B	Patr-C	HLA-A	HLA-B	HLA-C
No. of allelic groups*	24	22	7	15	32	27	27	24	37	15	21	36	14
No. of alleles	100	105	47	18	48	44	32	47	90	52	7894	9386	7872

Note. "*" indicates the number of distinct alleles at each locus according to the 2-digit nomenclature downloaded from the IPD. SLA, Swine leukocyte antigen; BoLA, Bovine leukocyte antigen; Patr, Pan troglodytes leukocyte antigen; HLA, Human leukocyte antigen; Ovar, Ovine leukocyte antigen. Ovar-N indicates the combined results of all sheep MHC class I alleles, owing to the unavailability of annotated allelic information for individual classical MHC class I genes.

Supplementary Table S2. The list of alleles in 20 different allelic groups of each MHC class I genes for each species based on 2-digint MHC nomenclature.

Species	Locus	Allele (accession number)
Human	HLA-A	HLA-A*01:01:01:01(HLA00001), HLA-A*02:01:01:01(HLA00005), HLA-A*03:01:01:01(HLA00037), HLA-A*11:01:01:01(HLA00043), HLA-A*23:01:01:01(HLA00048), HLA-A*24:02:01:01(HLA00050), HLA-A*25:01:01:01(HLA00071), HLA-A*26:01:01:01(HLA00073), HLA-A*29:01:01:01(HLA00085), HLA-A*30:01:01:01(HLA00089), HLA-A*31:01:02:01(HLA00097), HLA-A*32:01:01:01(HLA00101), HLA-A*33:01:01:01(HLA00104), HLA-A*34:01:01:01(HLA00108), HLA-A*36:01:01:01(HLA00110), HLA-A*43:01(HLA00111), HLA-A*66:01:01:01(HLA00112), HLA-A*68:01:01:01(HLA00115), HLA-A*69:01:01:01(HLA00126), HLA-A*74:01:01:01(HLA00127), HLA-A*80:01:01:01(HLA00130)
	HLA-B	HLA-B*07:02:01:01(HLA00132), HLA-B*08:01:01:01(HLA00146), HLA-B*13:01:01:01(HLA00152), HLA-B*14:01:01:01(HLA00157), HLA-B*15:01:01:01(HLA00162), HLA-B*18:01:01:01(HLA00213), HLA-B*27:01(HLA00220), HLA-B*35:01:01:01(HLA00237), HLA-B*37:01:01:01(HLA00265), HLA-B*38:01:01:01(HLA00267), HLA-B*39:01:01:01(HLA00271), HLA-B*40:01:01(HLA00291), HLA-B*41:01:01:01(HLA00312), HLA-B*42:01:01:01(HLA00315), HLA-B*44:02:01:01(HLA00318), HLA-B*45:01:01:01(HLA00329), HLA-B*46:01:01:01(HLA00331), HLA-B*47:01:01:02(HLA01437), HLA-B*48:01:01:01(HLA00335), HLA-B*49:01:01:01(HLA00340), HLA-B*50:01:01:01(HLA00341), HLA-B*51:01:01:01(HLA00344), HLA-B*52:01:01:01(HLA00362), HLA-B*53:01:01:01(HLA00364), HLA-B*54:01:01:01(HLA00367), HLA-B*55:01:01:01(HLA00368), HLA-B*56:01:01:01(HLA00376), HLA-B*57:01:01:01(HLA00381), HLA-B*58:01:01:01(HLA00386), HLA-B*59:01:01:01(HLA00389), HLA-B*67:01:01(HLA00390), HLA-B*73:01:01:01(HLA00392), HLA-B*78:02:01(HLA00394), HLA-B*81:01:01:01(HLA00398), HLA-B*82:01:01:01(HLA00399), HLA-B*83:01(HLA01135)

	HLA-C	HLA-C*01:02:01:01(HLA00401), HLA-C*02:02:01(HLA00404), HLA-C*03:02:01(HLA00410), HLA-C*04:01:01:01(HLA00420), HLA-C*05:01:01:01(HLA00427), HLA-C*06:02:01:01(HLA00430), HLA-C*07:01:01:01(HLA00433), HLA-C*08:01:01:01(HLA00445), HLA-C*12:02:01(HLA00453), HLA-C*14:02:01:01(HLA00462), HLA-C*15:02:01:01(HLA00467), HLA-C*16:01:01:01(HLA00475), HLA-C*17:01:01:02(HLA04311), HLA-C*18:01:01:01(HLA00483), HLA-A*01:01:01:01(HLA00662), HLA-A*01:01:01:01(HLA00485), HLA-A*01:01:01:01(HLA00494)
Sheep	Ovar-N	Ovar-N*01:01(OLA02432), Ovar-N*05:01(OLA02439), Ovar-N*06:01(OLA02443), Ovar-N*07:01(OLA02436), Ovar-N*08:1(OLA02438), Ovar-N*11:01(OLA02781), Ovar-N*12:01(OLA08695), Ovar-N*13:01(OLA08697), Ovar-N*14:01(OLA08698), Ovar-N*15:01(OLA08699), Ovar-N*16:01(OLA08700), Ovar-N*18:01(OLA08702), Ovar-N*19:01(OLA08703), Ovar-N*20:01(OLA8704), Ovar-N*21:01(OLA08705), Ovar-N*22:01(OLA08723), Ovar-N*24:01(OLA08724), Ovar-N*26:01(OLA0827), Ovar-N*27:01(OLA08728), Ovar-N*50:01(OLA02435)
Chimpanzee	Patr-A	Patr-A*01:01:01:01(NHP00705), Patr-A*02:01:01:01(NHP00706), Patr-A*03:01:01:01(NHP00707), Patr-A*04:01:01:01(NHP00709), Patr-A*05:01:01:01(NHP00712), Patr-A*06:01:01:01(NHP00713), Patr-A*07:01:01:01(NHP00715), Patr-A*08:01:01:01(NHP00716), Patr-A*09:01:01:01(NHP00719), Patr-A*10:01:01:01(NHP00720), Patr-A*11:01:01:01(NHP00721), Patr-A*12:01:01:01(NHP00722), Patr-A*13:01:01:01(NHP00723), Patr-A*14:01:01:01(NHP00724), Patr-A*15:01:01:01(NHP00725), Patr-A*16:01:01:01(NHP00727), Patr-A*17:01:01:01(NHP00728), Patr-A*18:01:01:01(NHP00730), Patr-A*21:01(NHP04210), Patr-A*22:01:01:01(NHP01096), Patr-A*24:01(NHP01991), Patr-A*25:01:01:01(NHP09272), Patr-A*26:01:01:01(NHP09274), Patr-A*27:01:01:01(NHP09275)

Patr-B	Patr-B*01:01:01:01(NHP00731), Patr-B*02:01(NHP00732), Patr-B*03:01(NHP00733), Patr-B*04:01(NHP00735), Patr-B*05:01:01:01(NHP00736), Patr-B*06:01(NHP00738), Patr-B*07:01(NHP00739), Patr-B*08:01(NHP00740), Patr-B*09:01:01:01(NHP00742), Patr-B*10:01(NHP00743), Patr-B*11:01(NHP00744), Patr-B*12:02(NHP00747), Patr-B*13:01:01:01(NHP00748), Patr-B*14:01:01:01(NHP00749), Patr-B*16:01:01(NHP00751), Patr-B*17:01(NHP00754), Patr-B*18:01:01:01(NHP00757), Patr-B*19:01(NHP00758), Patr-B*20:01:01(NHP00759), Patr-B*21:01:01:01(NHP00760), Patr-B*22:01:01:01(NHP00761), Patr-B*23:01:01(NHP00763), Patr-B*24:01:01:01(NHP00766), Patr-B*25:01(NHP00768), Patr-B*26:01(NHP00769), Patr-B*27:01(NHP00770), Patr-B*28:01(NHP00771), Patr-B*29:01:01:01(NHP00772), Patr-B*33:01:01:01(NHP04216), Patr-B*34:01(NHP04217), Patr-B*35:01(NHP01100), Patr-B*36:01:01:01(NHP02055), Patr-B*37:01(NHP02057), Patr-B*38:01(NHP05206), Patr-B*39:01(NHP05210), Patr-B*40:01:01:01(NHP09214), Patr-B*30:01(NHP00773)
Patr-C	Patr-C*02:01(NHP00775), Patr-C*03:01:01:01(NHP00778), Patr-C*04:01:01:01(NHP00782), Patr-C*05:01:01:01(NHP00783), Patr-C*06:01:01:01(NHP00785), Patr-C*07:01(NHP00786), Patr-C*08:01(NHP00787), Patr-C*09:01:01:01(NHP00788), Patr-C*10:01(NHP00792), Patr-C*11:01:01(NHP00793), Patr-C*12:01:01:01(NHP00794), Patr-C*13:01:01:01(NHP00795), Patr-C*15:01:01:01(NHP01993), Patr-C*16:01:01:01(NHP01994), Patr-C*17:01:01:01(NHP09240)

	BoLA-1	BoLA-1*007:01:01:01(BOLA03175), BoLA-1*007:01:01:02(BOLA10102), BoLA-1*020:01(BOLA03190), BoLA-1*019:01(BOLA03189), BoLA-1*061:01(BOLA07819), BoLA-1*097:01(BOLA10063), BoLA-1*021:01(BOLA03191), BoLA-1*028:01(BOLA03201), BoLA-1*031:01(BOLA03204), BoLA-1*031:02(BOLA07812), BoLA-1*042:01(BOLA03248), BoLA-1*009:02(BOLA03249), BoLA-1*009:01(BOLA03177), BoLA-1*023:01(BOLA03193), BoLA-1*029:01(BOLA03202), BoLA-1*074:01(BOLA07833), BoLA-1*049:01(BOLA03257), BoLA-1*067:01(BOLA07826)
Cattle	BoLA-2	BoLA-2*005:01(BOLA03173), BoLA-2*006:01(BOLA03174), BoLA-2*008:01(BOLA03176), BoLA-2*012:01(BOLA02982), BoLA-2*016:01(BOLA03185), BoLA-2*018:01(BOLA03187), BoLA-2*022:01(BOLA03192), BoLA-2*025:01(BOLA03196), BoLA-2*026:01(BOLA03197), BoLA-2*030:01(BOLA03203), BoLA-2*032:02(BOLA07813), BoLA-2*043:01(BOLA03250), BoLA-2*044:01(BOLA03251), BoLA-2*045:01(BOLA03252), BoLA-2*046:01(BOLA03253), BoLA-2*047:01(BOLA03254), BoLA-2*048:01(BOLA03255), BoLA-2*054:01(BOLA03263), BoLA-2*055:01(BOLA03264), BoLA-2*057:01(BOLA07815), BoLA-2*056:01(BOLA03265), BoLA-2*060:01(BOLA07818), BoLA-2*062:01(BOLA07820), BoLA-2*069:01(BOLA07828), BoLA-2*070:01(BOLA07829), BoLA-2*071:01(BOLA07830), BoLA-2*075:01(BOLA09830), BoLA-2*079:01(BOLA10068), BoLA-2*085:01(BOLA10064), BoLA-2*089:01(BOLA10067), BoLA-2*096:01(BOLA10107), BoLA-2*099:01(BOLA10189)
	BoLA-3	BoLA-3*001:01(BOLA03166), BoLA-3*002:01(BOLA03169), BoLA-3*004:01(BOLA03171), BoLA-3*010:01(BOLA03178), BoLA-3*011:01(BOLA03179), BoLA-3*017:01(BOLA03186), BoLA-3*027:01(BOLA03199), BoLA-3*035:01(BOLA03208), BoLA-3*036:01(BOLA03209), BoLA-3*037:01(BOLA03210), BoLA-3*038:01(BOLA03211), BoLA-3*050:01(BOLA03258), BoLA-3*051:01(BOLA03259), BoLA-3*052:01(BOLA03260), BoLA-3*053:01(BOLA03261), BoLA-3*058:01(BOLA07816), BoLA-3*059:01(BOLA07817), BoLA-3*065:01(BOLA07823), BoLA-3*066:01(BOLA07824), BoLA-3*068:01(BOLA07827), BoLA-3*073:01(BOLA07832), BoLA-3*078:01(BOLA10076), BoLA-3*080:01(BOLA10081), BoLA-3*081:01(BOLA10082), BoLA-3*082:01(BOLA10085), BoLA-3*083:01(BOLA10072), BoLA-3*087:01(BOLA10075)

Pig	SLA-1	SLA-1*01:01(SLA06100), SLA-1*02:01(SLA06103), SLA-1*04:01:01(SLA06106), SLA-1*05:01(SLA06110), SLA-1*06:01(SLA06111), SLA-1*07:01(SLA06113), SLA-1*08:01(SLA06115), SLA-1*09:01(SLA08449), SLA-1*10:01(SLA06143), SLA-1*11:01:01(SLA06123), SLA-1*12:01(SLA06128), SLA-1*13:01(SLA06131), SLA-1*14:01(SLA06137), SLA-1*15:01(SLA08457), SLA-1*16:01(SLA06133), SLA-1*17:01(SLA06135), SLA-1*18:01(SLA06139), SLA-1*19:01(SLA08461), SLA-1*20:01(SLA08462), SLA-1*21:01(SLA09713), SLA-1*22:01(SLA08463), SLA-1*23:01(SLA09717), SLA-1*24:01(SLA09783), SLA-1*25:01(SLA09784)
	SLA-2	SLA-2*01:01(SLA08464), SLA-2*02:01(SLA08466), SLA-2*03:01(SLA06145), SLA-2*04:01(SLA06148), SLA-2*05:01(SLA06151), SLA-2*06:01(SLA06157), SLA-2*07:01(SLA06162), SLA-2*08:01(SLA08491), SLA-2*09:01(SLA06181), SLA-2*10:01(SLA06165), SLA-2*11:01:01(SLA06170), SLA-2*12:01(SLA06173), SLA-2*13:01(SLA06184), SLA-2*14:01(SLA06185), SLA-2*15:01(SLA06177), SLA-2*16:01(SLA06221), SLA-2*17:02(SLA09786), SLA-2*18:01(SLA08503), SLA-2*19:01(SLA09753), SLA-2*20:01(SLA09752), SLA-2*21:01(SLA09773), SLA-2*22:01(SLA09801)
	SLA-3	SLA-3*01:01(SLA06186), SLA-3*03:01(SLA06190), SLA-3*04:01(SLA06198), SLA-3*05:01(SLA06201), SLA-3*06:01(SLA06205), SLA-3*07:01:01(SLA06207), SLA-3*08:01(SLA06211)
Platypus	-	AY112715*

*Annotated information on the MHC class I genes of platypus is not available.

Supplementary Table S3. Pairwise nucleotide sequence differences in exons 2 and 3 of the classical MHC class I genes in pigs, cattle, chimpanzees, and humans

	Sequence differences (%) [a]											
	SLA-1	SLA-2	SLA-3	BoLA-2	BoLA-3	BoLA-1	Patr-B	HLA-B	Patr-C	HLA-C	Patr-A	HLA-A
SLA-1												
SLA-2	7.82											
SLA-3	8.77	8.79										
BoLA-2	14.20	14.19	14.33									
BoLA-3	15.03	15.05	14.76	9.51								
BoLA-1	15.49	15.35	15.28	10.44	12.31							
Patr-B	15.53	15.29	14.86	14.44	15.18	14.86						
HLA-B	15.47	15.17	14.84	14.63	15.36	15.02	6.65					
Patr-C	16.13	15.99	15.64	15.02	15.22	16.30	8.83	8.39				
HLA-C	15.57	15.39	14.92	14.50	14.65	16.02	8.01	7.42	3.85			
Patr-A	15.22	15.39	15.15	15.32	16.26	16.56	10.04	10.87	11.22	10.65		
HLA-A	15.03	15.26	15.00	15.10	15.88	16.16	9.26	9.86	10.67	9.74	5.10	

[a] The numbers indicate the mean nucleotide sequence differences (%) in the pairwise comparisons of analyzed alleles (n=20 for each gene) between the two different genes. Note. SLA, Swine leukocyte antigen; BoLA, Bovine leukocyte antigen; Patr, Pan troglodytes leukocyte antigen; HLA, Human leukocyte antigen; Ovar, Ovine leukocyte antigen.

Supplementary Table S4. Comparison of the estimated genetic diversity levels for classical MHC class I genes in humans, chimpanzees, cattle, and pigs using all allelic groups and 20 representative allelic groups.

Species	Locus	No. of allelic groups [a]	No. of variable sites (A)		No. of variations (B) [b]		(B) / (A) ratio [c]	
			From all allelic groups	From 20 allelic groups	From all allelic groups	From 20 allelic groups	From total allelic groups	From 20 allelic groups
Human	HLA-A	21	71	71	85	85	1.20	1.20
	HLA-B	36	89	83	111	103	1.25	1.24
	HLA-C	14	51	58	59	68	1.16	1.17
	Mean	23.67	70.33	70.67	85.00	85.33	1.20	1.20
Chimpanzee	Patr-A	24	54	54	65	64	1.20	1.19
	Patr-B	37	107	100	128	118	1.20	1.18
	Patr-C	15	51	52	56	57	1.10	1.10
	Mean	25.33	70.67	68.67	83.00	79.67	1.17	1.15
Cattle	BoLA-1	15	128	128	155	155	1.21	1.21
	BoLA-2	32	135	124	177	164	1.31	1.32
	BoLA-3	27	126	119	164	156	1.30	1.31
	Mean	24.67	129.67	123.67	165.33	158.33	1.27	1.28
Pig	SLA-1	24	131	125	169	163	1.29	1.30
	SLA-2	22	136	135	173	172	1.27	1.27
	SLA-3	7	69	105	80	125	1.16	1.19
	Mean	17.67	112.00	121.67	140.67	153.33	1.24	1.26

[a] The entire allelic groups using 2-digit MHC nomenclature available in IPD.

[b] Number of observed variations, including changes in different amino acids at each site.

[c] The number of variations per variable site.

Supplementary Table S5. Variable sites were identified in the peptide-binding region of classical MHC class I genes in pigs, cattle, sheep, chimpanzees, and humans.

Species	Locus	Positions of synonymous SNPs (total number)	Positions of non-synonymous SNPs (total number)
Human	HLA-A	5 29 50 53 71 107 146 170 338 350 375 416 (12)	48 90 103 130 186 188 192 197 198 209 217 219 226 228 229 234 238 240 241 244 246 268 282 289 290 295 303 312 318 324 345 380 424 429 433 444 448 451 472 482 497 498 510 (43)
	HLA-B	44 71 92 102 131 140 149 155 173 212 236 281 314 338 404 413 545 (17)	24 30 33 69 94 120 136 153 183 186 188 193 196 197 204 205 210 219 226 228 229 238 240 241 244 246 268 280 306 336 339 390 426 433 439 454 471 499 510 530 532 537 (42)
	HLA-C	29 32 44 53 128 140 242 314 380 386 404 521 545 (13)	16 30 39 45 61 69 103 145 197 216 226 229 239 268 280 282 288 306 336 339 422 426 439 453 454 507 516 528 530 532 537 (31)
Chimpanzee	Patr-A	29 32 146 167 191 197 218 335 338 341 374 380 416 425 486 545 (16)	25 87 183 184 196 210 217 219 226 228 288 295 339 340 409 429 433 440 448 454 463 487 492 497 498 499 543 (27)
	Patr-B	29 71 74 80 89 92 131 140 149 170 185 206 251 269 272 281 296 338 356 371 413 425 (22)	8 16 25 26 28 30 33 48 69 102 124 132 133 144 155 179 183 186 188 193 195 196 204 210 214 219 226 228 229 234 238 240 241 243 244 246 255 256 268 280 288 289 295 306 324 357 369 381 381 390 414 426 439 440 450 497 498 532 (58)
	Patr-C	32 71 74 86 92 128 185 194 263 281 371 404 428 545 (10)	12 24 51 61 69 112 121 180 184 186 196 197 229 239 282 295 346 369 403 439 454 486 487 489 499 501 502 510 526 543 (30)
Cattle	BoLA-1	11 35 38 59 62 68 74 83 92 95 128 131 137 140 164 167 170 173 191 197 221 231 281 320 323 338 449 458 479 533 (30)	**12** 16 24 **31** 48 49 **66** 69 93 96 99 100 102 **120** 121 123 144 145 155 159 162 182 **186** 187 195 196 198 206 210 211 214 220 223 **238** **244** 280 293 295 312 321 324 330 331 337 345 346 **349** **356** 357 **369** 386 399 403 432 448 451 477 **498** 531 (59)

	BoLA-2	11 56 59 62 68 80 101 128 137 140 161 170 224 227 281 287 299 317 320 338 350 458 518 (23)	**12** 16 **31** 48 60 64 **66** 99 100 102 114 117 **120** 123 145 148 153 159 169 **186** 188 205 206 207 208 210 226 237 **238** **244** 267 280 282 295 303 316 328 337 345 346 **349** **356** 363 **369** 381 386 415 438 448 451 477 **498** 510 520 531 (55)
	BoLA-3	11 62 68 80 107 131 137 147 170 192 215 224 242 296 317 323 326 335 338 350 359 359 431 449 458 521 (26)	**12** **31** 46 48 64 **66** 87 93 **120** 121 123 153 156 169 **186** 188 193 198 199 205 220 226 228 229 **238** 240 241 **244** 282 295 303 321 324 328 330 337 345 346 **349** **356** 369 **386** 406 410 412 422 426 445 448 457 469 **498** 510 520 531 (55)
Pig	SLA-1	62 98 128 140 143 191 206 239 242 296 311 401 458 470 477 485 506 (17)	17 49 54 56 60 66 112 114 126 132 133 145 148 156 159 169 172 173 186 188 193 205 210 217 222 226 228 229 238 240 241 244 280 295 303 306 324 331 349 369 412 426 441 443 450 454 480 492 498 499 505 507 508 516 525 (55)
	SLA-2	29 62 77 102 122 128 129 140 143 155 158 164 183 191 222 242 245 254 296 311 326 329 332 413 419 446 458 485 506 (29)	3 17 30 42 49 54 56 57 66 112 114 126 130 145 148 156 159 173 183 184 186 188 193 205 206 210 213 226 228 229 240 241 244 246 265 280 295 303 306 324 331 349 357 369 412 426 443 444 450 454 480 498 499 505 507 508 516 525 (58)
	SLA-3	86 122 140 200 227 245 305 320 371 447 467 467 479 515 533 (15)	17 24 25 37 49 51 57 102 114 121 138 145 148 156 159 162 163 180 182 195 197 201 202 205 206 214 217 219 226 229 238 245 247 265 291 312 315 330 333 340 348 349 358 378 399 415 421 435 447 448 454 459 463 465 474 475 489 505 507 508 514 516 525 527 528 534 536 537 550 552 (70)
Sheep	Ovar-N	38 62 68 77 122 131 137 140 141 149 170 188 191 206 242 266 311 314 329 338 375 413 449 470 479 503 (26)	3 **12** 21 **31** 49 64 **66** 91 **120** 130 139 148 153 155 163 **186** 188 203 205 210 211 214 217 226 228 229 235 **238** 240 241 **244** 267 303 305 316 330 337 **349** **356** **369** 381 394 411 423 426 429 433 437 442 444 445 448 472 477 481 **498** 499 505 510 520 538 (61)

Note. Common variable sites across paralogous MHC class I genes within a species are indicated by different colors. Only the common variable sites across SLA, Ovar, and HLA are indicated in yellow, and those across Patr and BoLA are indicated in green. Common variable sites across the BoLA, SLA, and ovars are indicated in blue. Red indicates the common variable sites across paralogous MHC class

I genes within a species. SLA, Swine leukocyte antigen; BoLA, Bovine leukocyte antigen; Patr, Pan troglodytes leukocyte antigen; HLA, Human leukocyte antigen; Ovar, Ovine leukocyte antigen. Ovar-N indicates the combined results of all sheep MHC class I alleles, owing to the availability of annotated allelic information for individual classical MHC class I genes.

Supplementary Table S6. Identified peptide biding pockets of MHC class I proteins based on the structure of HLA class I reported by Nguyen, et al. (2021)

Pocket	Participating amino acid residues [a]
A	4, 6, 58, 62, 65, 158, 162, 166, 170
B	6, 8, 23, 33, 44, 62, 65, 66, 69, 98
C	8, 23, 33, 69, 72, 73, 96
D	98, 113, 154, 155, 158, 159
E	96, 113, 146, 151, 155
F	76, 79, 80, 83, 94, 115, 122, 142, 145, 146

[a] The first amino acid of exon 2 and the last amino acid of exon 3 correspond to residues 1 and 181, respectively. The residue numbers were the same across all four species.

Supplementary Table S7. Comparison of amino acid sequence diversity among the predicted peptide-binding pocket sites of classical MHC class I genes in pigs, cattle, chimpanzees, and humans

**MHC genes**	**Difference in Shannon diversity index between compared peptide-binding pockets** [a]	**P-value** [b]
SLA-1, -2, and -3	C : A (-26.837) [c]	0.0013
	E : A (-34.370)	0.0001
	E : B (-22.500)	0.0056
	D : C (17.300)	0.0340
	F : C (19.133)	0.0179
	E : D (-24.833)	0.0025
	F : E (26.667)	0.0014
BoLA-1, -2, and -3	E : A (-31.610)	0.0009
	E : B (-22.000)	0.0161
	E : C (-18.507)	0.0473
	F : E (28.187)	0.0025
Patr-A, -B, and -C	E : A (-34.590)	0.0004
	E : B (-23.833)	0.0082
	E : C (-18.430)	0.0444
	E : D (-20.610)	0.0224
	F : E (29.167)	0.0016
HLA-A, -B, and -C	E : A (-23.517)	0.0250

[a] Indicates statistical significance in the sequence difference ($p<0.05$) using the Shannon diversity index among different peptide-binding pockets.

[b] indicates the p-value in the results of the two-way ANOVA, while paralogous genes and binding pockets are two different parameters. p-value from two-way ANOVA.

[c] indicates the mean difference between Shannon diversity indices of the two pockets. The numbers in parentheses correspond to the value of the second pocket minus that of the first pocket.

Note. SLA, Swine leukocyte antigen; BoLA, Bovine leukocyte antigen; Patr, Pan troglodytes leukocyte antigen; HLA, Human leukocyte antigen; Ovar, Ovine leukocyte antigen.

Supplementary Table S8. Frequency of amino acids for each residue constituting the peptide-binding region of 20 SLA-1 alleles from different allelic groups according to the 2-digit nomenclature

Position	A	C	D	E	F	G	H	I	K	L	M	N	P	Q	R	S	T	V	W	Y
1	0	0	0	0	0	0	0	0	0	0	0	0	20	0	0	0	0	0	0	0
2	0	0	0	0	0	0	20	0	0	0	0	0	0	0	0	0	0	0	0	0
3	0	0	0	0	0	0	0	0	0	0	0	0	0	0	0	20	0	0	0	0
4	0	0	0	0	0	0	0	0	0	20	0	0	0	0	0	0	0	0	0	0
5	0	0	0	0	0	0	0	0	0	0	0	0	0	0	3	17	0	0	0	0
6	0	0	0	0	0	0	0	0	0	0	0	0	0	0	0	0	0	0	0	20
7	0	0	0	0	20	0	0	0	0	0	0	0	0	0	0	0	0	0	0	0
8	0	0	2	0	4	0	0	0	0	0	0	0	0	0	0	3	0	0	0	11
9	0	0	0	0	0	0	0	0	0	0	0	0	0	0	0	0	20	0	0	0
10	20	0	0	0	0	0	0	0	0	0	0	0	0	0	0	0	0	0	0	0
11	0	0	0	0	0	0	0	0	0	0	0	0	0	0	0	0	0	20	0	0
12	0	0	0	0	0	0	0	0	0	0	0	0	0	0	0	20	0	0	0	0
13	0	0	0	0	0	0	0	0	0	0	0	0	0	0	20	0	0	0	0	0
14	0	0	0	0	0	0	0	0	0	0	0	0	20	0	0	0	0	0	0	0
15	0	0	20	0	0	0	0	0	0	0	0	0	0	0	0	0	0	0	0	0
16	0	0	0	0	0	0	0	0	0	3	0	0	0	0	17	0	0	0	0	0
17	0	0	0	0	0	20	0	0	0	0	0	0	0	0	0	0	0	0	0	0
18	0	0	15	3	0	0	0	0	2	0	0	0	0	0	0	0	0	0	0	0
19	0	0	0	0	0	0	0	0	0	0	0	0	4	0	0	15	1	0	0	0
20	0	1	0	0	0	0	0	0	0	0	0	0	0	0	19	0	0	0	0	0
21	0	0	0	0	20	0	0	0	0	0	0	0	0	0	0	0	0	0	0	0
22	0	0	0	0	3	0	0	17	0	0	0	0	0	0	0	0	0	0	0	0
23	12	0	0	3	0	0	0	3	0	0	0	0	0	0	0	2	0	0	0	0
24	0	0	0	0	0	0	0	0	0	0	0	0	0	0	0	0	0	20	0	0
25	0	0	0	0	0	20	0	0	0	0	0	0	0	0	0	0	0	0	0	0
26	0	0	0	0	0	0	0	0	0	0	0	0	0	0	0	0	0	0	0	20
27	0	0	0	0	0	0	0	0	0	0	0	0	0	0	0	0	0	20	0	0
28	0	0	20	0	0	0	0	0	0	0	0	0	0	0	0	0	0	0	0	0
29	0	0	20	0	0	0	0	0	0	0	0	0	0	0	0	0	0	0	0	0
30	0	0	0	0	0	0	0	0	0	0	0	0	0	0	0	0	20	0	0	0
31	0	0	0	0	0	0	0	0	0	0	0	0	0	20	0	0	0	0	0	0
32	0	0	0	0	20	0	0	0	0	0	0	0	0	0	0	0	0	0	0	0

Position	A	C	D	E	F	G	H	I	K	L	M	N	P	Q	R	S	T	V	W	Y
33	0	0	0	0	0	0	0	0	0	0	0	0	0	0	0	0	0	20	0	0
34	0	0	0	0	0	0	0	0	0	0	0	0	0	0	20	0	0	0	0	0
35	0	0	0	0	20	0	0	0	0	0	0	0	0	0	0	0	0	0	0	0
36	0	0	20	0	0	0	0	0	0	0	0	0	0	0	0	0	0	0	0	0
37	0	0	0	0	0	0	0	0	0	0	0	2	0	0	0	18	0	0	0	0
38	0	0	18	0	0	0	0	0	0	0	0	0	0	0	0	0	0	0	0	2
39	20	0	0	0	0	0	0	0	0	0	0	0	0	0	0	0	0	0	0	0
40	0	0	0	0	0	0	0	0	0	1	0	0	19	0	0	0	0	0	0	0
41	0	0	0	0	0	0	0	0	0	0	0	20	0	0	0	0	0	0	0	0
42	2	0	0	0	0	0	0	0	0	0	0	0	18	0	0	0	0	0	0	0
43	0	0	0	0	0	0	0	0	2	0	0	0	0	0	18	0	0	0	0	0
44	0	0	0	2	0	0	0	0	0	0	18	0	0	0	0	0	0	0	0	0
45	0	0	0	20	0	0	0	0	0	0	0	0	0	0	0	0	0	0	0	0
46	0	0	0	0	0	0	0	0	0	0	0	0	20	0	0	0	0	0	0	0
47	0	0	0	0	0	0	0	0	0	0	0	0	0	0	20	0	0	0	0	0
48	16	0	0	0	0	0	0	0	0	0	0	0	0	0	0	0	0	4	0	0
49	0	0	0	0	0	0	0	0	0	0	0	0	18	1	1	0	0	0	0	0
50	0	0	0	0	0	0	0	0	0	0	0	0	0	0	0	0	0	0	20	0
51	0	0	0	0	0	0	0	20	0	0	0	0	0	0	0	0	0	0	0	0
52	0	0	0	7	0	0	0	0	1	0	0	0	0	12	0	0	0	0	0	0
53	0	0	0	0	0	0	0	0	5	0	0	0	0	15	0	0	0	0	0	0
54	0	0	0	20	0	0	0	0	0	0	0	0	0	0	0	0	0	0	0	0
55	0	0	0	0	0	20	0	0	0	0	0	0	0	0	0	0	0	0	0	0
56	0	0	0	0	0	0	0	0	0	0	0	0	2	18	0	0	0	0	0	0
57	0	0	9	10	0	1	0	0	0	0	0	0	0	0	0	0	0	0	0	0
58	0	0	0	0	0	0	0	0	0	0	0	0	0	0	0	0	0	0	0	20
59	0	0	0	0	0	0	0	0	0	0	0	0	0	0	0	0	0	0	20	0
60	0	0	20	0	0	0	0	0	0	0	0	0	0	0	0	0	0	0	0	0
61	0	0	0	8	0	0	0	0	0	0	0	0	0	1	11	0	0	0	0	0
62	0	0	0	17	0	0	0	0	0	0	0	2	0	1	0	0	0	0	0	0
63	0	0	0	0	0	0	0	0	0	0	0	0	0	0	0	0	20	0	0	0
64	0	0	0	0	0	0	0	0	0	0	0	0	0	6	14	0	0	0	0	0
65	0	0	0	0	0	0	0	5	8	0	0	5	0	0	2	0	0	0	0	0
66	4	0	0	0	0	0	0	1	0	0	0	0	0	3	0	3	0	8	0	1
67	0	0	0	0	0	0	0	0	11	0	5	0	0	0	3	0	0	1	0	0
68	0	0	8	6	0	6	0	0	0	0	0	0	0	0	0	0	0	0	0	0

Position	A	C	D	E	F	G	H	I	K	L	M	N	P	Q	R	S	T	V	W	Y
69	0	0	0	1	1	0	0	0	0	0	0	6	0	0	0	3	9	0	0	0
70	17	0	0	0	0	0	0	0	0	0	0	0	0	0	0	3	0	0	0	0
71	0	0	0	0	0	0	0	0	0	0	0	0	0	20	0	0	0	0	0	0
72	0	0	0	0	0	0	0	5	0	0	0	1	0	0	0	0	14	0	0	0
73	0	0	1	0	6	0	0	0	0	1	0	5	0	0	0	0	0	0	0	7
74	0	0	0	0	0	1	0	0	0	0	0	0	0	0	19	0	0	0	0	0
75	0	0	0	0	0	2	0	0	0	0	0	0	0	0	0	0	0	18	0	0
76	0	0	6	0	0	6	0	0	0	0	0	6	0	0	0	2	0	0	0	0
77	0	0	0	0	0	0	0	0	0	20	0	0	0	0	0	0	0	0	0	0
78	0	0	0	0	0	0	0	0	9	0	0	8	0	0	3	0	0	0	0	0
79	0	0	0	0	0	0	0	0	0	0	0	4	0	0	0	0	16	0	0	0
80	3	0	0	0	0	0	0	0	0	17	0	0	0	0	0	0	0	0	0	0
81	0	0	0	0	0	0	0	0	0	3	0	0	0	0	17	0	0	0	0	0
82	0	0	0	0	0	20	0	0	0	0	0	0	0	0	0	0	0	0	0	0
83	0	0	0	0	0	0	0	0	0	0	0	0	0	0	0	0	0	0	0	20
84	0	0	0	0	0	0	0	0	0	0	0	0	0	0	0	0	0	0	0	20
85	0	0	0	0	0	0	0	0	0	0	0	20	0	0	0	0	0	0	0	0
86	0	0	0	0	0	0	0	0	0	0	0	0	0	20	0	0	0	0	0	0
87	0	0	0	0	0	0	0	0	0	0	0	0	0	0	0	20	0	0	0	0
88	0	0	0	20	0	0	0	0	0	0	0	0	0	0	0	0	0	0	0	0
89	20	0	0	0	0	0	0	0	0	0	0	0	0	0	0	0	0	0	0	0
90	0	0	0	0	0	20	0	0	0	0	0	0	0	0	0	0	0	0	0	0
91	0	0	0	0	0	0	0	0	0	0	0	0	0	0	0	20	0	0	0	0
92	0	0	0	0	0	0	20	0	0	0	0	0	0	0	0	0	0	0	0	0
93	0	0	0	0	0	0	0	0	0	0	0	2	0	0	0	0	18	0	0	0
94	0	0	0	0	1	0	0	7	0	11	0	0	0	0	0	0	0	0	0	1
95	0	0	0	0	0	0	0	0	0	0	0	0	0	20	0	0	0	0	0	0
96	0	0	0	0	0	0	0	3	0	0	0	0	0	0	3	8	4	0	2	0
97	0	0	0	0	0	0	0	0	0	0	20	0	0	0	0	0	0	0	0	0
98	0	0	0	0	4	0	0	0	0	0	0	0	0	0	0	0	0	0	0	16
99	0	0	0	0	0	20	0	0	0	0	0	0	0	0	0	0	0	0	0	0
100	0	20	0	0	0	0	0	0	0	0	0	0	0	0	0	0	0	0	0	0
101	0	0	10	0	0	0	0	0	0	0	0	0	0	0	0	0	0	0	0	10
102	0	0	0	0	0	0	0	0	0	10	0	0	0	0	0	0	0	10	0	0
103	0	0	0	0	0	20	0	0	0	0	0	0	0	0	0	0	0	0	0	0
104	0	0	0	0	0	0	0	0	0	0	0	0	20	0	0	0	0	0	0	0

Position	A	C	D	E	F	G	H	I	K	L	M	N	P	Q	R	S	T	V	W	Y
105	0	0	20	0	0	0	0	0	0	0	0	0	0	0	0	0	0	0	0	0
106	0	0	0	0	0	20	0	0	0	0	0	0	0	0	0	0	0	0	0	0
107	0	0	0	0	0	0	0	0	0	20	0	0	0	0	0	0	0	0	0	0
108	0	0	0	0	1	0	0	0	0	19	0	0	0	0	0	0	0	0	0	0
109	0	0	0	0	0	0	0	0	0	20	0	0	0	0	0	0	0	0	0	0
110	0	0	0	0	0	0	3	0	0	0	0	0	0	0	17	0	0	0	0	0
111	0	0	0	0	0	20	0	0	0	0	0	0	0	0	0	0	0	0	0	0
112	0	0	0	0	0	0	0	0	0	0	0	0	0	0	0	0	0	0	0	20
113	0	0	2	0	0	0	0	0	0	0	0	0	0	0	10	8	0	0	0	0
114	0	0	0	0	0	0	0	0	0	0	0	0	0	20	0	0	0	0	0	0
115	0	0	15	0	3	0	0	0	0	0	0	0	0	0	0	0	0	0	0	2
116	16	0	0	0	0	4	0	0	0	0	0	0	0	0	0	0	0	0	0	0
117	0	0	0	0	0	0	0	0	0	0	0	0	0	0	0	0	0	0	0	20
118	0	0	20	0	0	0	0	0	0	0	0	0	0	0	0	0	0	0	0	0
119	0	0	0	0	0	20	0	0	0	0	0	0	0	0	0	0	0	0	0	0
120	20	0	0	0	0	0	0	0	0	0	0	0	0	0	0	0	0	0	0	0
121	0	0	20	0	0	0	0	0	0	0	0	0	0	0	0	0	0	0	0	0
122	0	0	0	0	0	0	0	0	0	0	0	0	0	0	0	0	0	0	0	20
123	0	0	0	0	0	0	0	15	0	3	0	0	0	0	0	0	0	2	0	0
124	20	0	0	0	0	0	0	0	0	0	0	0	0	0	0	0	0	0	0	0
125	0	0	0	0	0	0	0	0	0	20	0	0	0	0	0	0	0	0	0	0
126	0	0	0	0	0	0	0	0	0	0	0	20	0	0	0	0	0	0	0	0
127	0	0	0	20	0	0	0	0	0	0	0	0	0	0	0	0	0	0	0	0
128	0	0	20	0	0	0	0	0	0	0	0	0	0	0	0	0	0	0	0	0
129	0	0	0	0	0	0	0	0	0	20	0	0	0	0	0	0	0	0	0	0
130	0	0	0	0	0	0	0	0	0	0	0	0	0	0	20	0	0	0	0	0
131	0	0	0	0	0	0	0	0	0	0	0	0	0	0	0	20	0	0	0	0
132	0	0	0	0	0	0	0	0	0	0	0	0	0	0	0	0	0	0	20	0
133	0	0	0	0	0	0	0	0	0	0	0	0	0	0	0	0	20	0	0	0
134	20	0	0	0	0	0	0	0	0	0	0	0	0	0	0	0	0	0	0	0
135	20	0	0	0	0	0	0	0	0	0	0	0	0	0	0	0	0	0	0	0
136	0	0	20	0	0	0	0	0	0	0	0	0	0	0	0	0	0	0	0	0
137	0	0	0	0	0	0	0	0	0	0	9	0	0	0	0	0	11	0	0	0
138	20	0	0	0	0	0	0	0	0	0	0	0	0	0	0	0	0	0	0	0
139	20	0	0	0	0	0	0	0	0	0	0	0	0	0	0	0	0	0	0	0
140	0	0	0	0	0	0	0	0	0	0	0	0	0	20	0	0	0	0	0	0

Position	A	C	D	E	F	G	H	I	K	L	M	N	P	Q	R	S	T	V	W	Y
141	0	0	0	0	0	0	0	20	0	0	0	0	0	0	0	0	0	0	0	0
142	0	0	0	0	0	0	0	0	0	0	0	0	0	0	0	5	15	0	0	0
143	0	0	0	0	0	0	0	0	20	0	0	0	0	0	0	0	0	0	0	0
144	0	0	0	0	0	0	0	0	0	0	0	0	0	0	20	0	0	0	0	0
145	0	0	0	0	0	0	0	0	20	0	0	0	0	0	0	0	0	0	0	0
146	0	0	0	0	1	0	0	0	0	0	0	0	0	1	1	0	0	0	17	0
147	0	0	1	17	0	0	0	0	2	0	0	0	0	0	0	0	0	0	0	0
148	17	0	0	1	0	0	0	0	0	0	0	0	0	0	0	0	2	0	0	0
149	20	0	0	0	0	0	0	0	0	0	0	0	0	0	0	0	0	0	0	0
150	0	0	12	0	0	0	2	0	0	0	0	6	0	0	0	0	0	0	0	0
151	5	0	0	7	0	0	0	0	0	0	0	0	0	0	0	0	0	8	0	0
152	20	0	0	0	0	0	0	0	0	0	0	0	0	0	0	0	0	0	0	0
153	0	0	0	20	0	0	0	0	0	0	0	0	0	0	0	0	0	0	0	0
154	0	0	0	0	0	0	0	0	0	0	0	0	0	8	12	0	0	0	0	0
155	0	0	0	3	0	0	0	0	0	1	9	0	0	0	4	0	0	0	3	0
156	0	0	0	0	0	0	0	0	0	0	0	0	0	0	20	0	0	0	0	0
157	0	0	0	0	0	0	0	0	0	0	0	0	0	0	0	20	0	0	0	0
158	0	0	0	0	0	0	0	0	0	0	0	0	0	0	0	0	0	0	0	20
159	0	0	0	0	0	0	0	0	0	20	0	0	0	0	0	0	0	0	0	0
160	0	0	0	1	0	0	0	0	0	0	0	0	0	19	0	0	0	0	0	0
161	0	0	0	0	0	20	0	0	0	0	0	0	0	0	0	0	0	0	0	0
162	1	0	0	0	0	0	0	0	0	10	0	0	0	0	5	0	4	0	0	0
163	0	20	0	0	0	0	0	0	0	0	0	0	0	0	0	0	0	0	0	0
164	0	0	0	0	0	0	0	0	0	1	0	0	0	0	0	0	0	19	0	0
165	0	0	0	20	0	0	0	0	0	0	0	0	0	0	0	0	0	0	0	0
166	0	0	0	0	0	6	0	0	0	0	0	0	0	0	0	7	0	0	7	0
167	0	0	0	0	0	0	0	0	0	20	0	0	0	0	0	0	0	0	0	0
168	0	0	0	0	0	0	0	0	0	0	0	0	0	11	9	0	0	0	0	0
169	0	0	0	4	0	0	0	0	13	0	0	0	0	0	3	0	0	0	0	0
170	0	0	0	0	0	0	0	0	0	0	0	0	0	0	0	0	0	0	0	20
171	0	0	0	0	0	0	0	0	0	20	0	0	0	0	0	0	0	0	0	0
172	0	0	0	19	0	0	0	0	0	0	0	0	0	1	0	0	0	0	0	0
173	0	0	0	0	0	0	0	0	0	0	20	0	0	0	0	0	0	0	0	0
174	0	0	0	0	0	20	0	0	0	0	0	0	0	0	0	0	0	0	0	0
175	0	0	0	3	0	0	0	0	17	0	0	0	0	0	0	0	0	0	0	0
176	0	0	20	0	0	0	0	0	0	0	0	0	0	0	0	0	0	0	0	0

Position	A	C	D	E	F	G	H	I	K	L	M	N	P	Q	R	S	T	V	W	Y
177	0	0	0	0	0	0	0	0	0	0	0	0	0	0	0	0	20	0	0	0
178	0	0	0	0	0	0	0	0	0	20	0	0	0	0	0	0	0	0	0	0
179	0	0	0	0	0	0	0	0	0	0	0	0	0	20	0	0	0	0	0	0
180	0	0	0	0	0	0	0	0	0	0	0	0	0	0	20	0	0	0	0	0
181	20	0	0	0	0	0	0	0	0	0	0	0	0	0	0	0	0	0	0	0

Supplementary Table S9. Frequency of amino acids for each residue constituting the peptide-binding region of 20 SLA-2 alleles from different allelic groups according to the 2-digit nomenclature

Position	A	C	D	E	F	G	H	I	K	L	M	N	P	Q	R	S	T	V	W	Y
1	0	0	0	0	0	0	0	0	0	0	0	0	18	0	0	2	0	0	0	0
2	0	0	0	0	0	0	20	0	0	0	0	0	0	0	0	0	0	0	0	0
3	0	0	0	0	0	0	0	0	0	0	0	0	0	0	0	20	0	0	0	0
4	0	0	0	0	0	0	0	0	0	20	0	0	0	0	0	0	0	0	0	0
5	0	0	0	0	0	0	0	0	0	0	0	0	0	0	4	16	0	0	0	0
6	0	0	0	0	0	0	0	0	0	0	0	0	0	0	0	0	0	0	0	20
7	0	0	0	0	20	0	0	0	0	0	0	0	0	0	0	0	0	0	0	0
8	0	0	2	0	1	0	3	0	0	0	0	0	0	0	0	4	0	0	0	10
9	0	0	0	0	0	0	0	0	0	0	0	0	0	0	0	0	20	0	0	0
10	18	0	0	0	0	0	0	0	0	0	0	0	0	0	0	0	2	0	0	0
11	0	0	0	0	0	0	0	0	0	0	0	0	0	0	0	0	0	20	0	0
12	0	0	0	0	0	0	0	0	0	0	0	0	0	0	0	20	0	0	0	0
13	0	0	0	0	0	0	0	0	0	0	0	0	0	0	20	0	0	0	0	0
14	0	0	0	0	0	0	0	0	0	0	0	0	19	0	0	1	0	0	0	0
15	0	0	20	0	0	0	0	0	0	0	0	0	0	0	0	0	0	0	0	0
16	0	0	0	0	0	0	0	0	0	4	0	0	0	0	16	0	0	0	0	0
17	0	0	0	0	0	20	0	0	0	0	0	0	0	0	0	0	0	0	0	0
18	0	0	18	1	0	0	0	0	1	0	0	0	0	0	0	0	0	0	0	0
19	0	0	0	0	0	0	0	0	0	0	0	0	2	0	0	18	0	0	0	0
20	0	0	0	0	0	0	0	0	0	0	0	0	0	0	20	0	0	0	0	0
21	0	0	0	0	20	0	0	0	0	0	0	0	0	0	0	0	0	0	0	0
22	0	0	0	0	1	0	0	12	0	7	0	0	0	0	0	0	0	0	0	0
23	9	0	0	2	0	0	0	1	0	0	0	0	0	0	0	1	7	0	0	0
24	0	0	0	0	0	0	0	0	0	0	0	0	0	0	0	0	0	20	0	0
25	0	0	0	0	0	20	0	0	0	0	0	0	0	0	0	0	0	0	0	0
26	0	0	0	0	0	0	0	0	0	0	0	0	0	0	0	0	0	0	0	20
27	0	0	0	0	0	0	0	0	0	0	0	0	0	0	0	0	0	20	0	0
28	0	0	20	0	0	0	0	0	0	0	0	0	0	0	0	0	0	0	0	0
29	0	0	20	0	0	0	0	0	0	0	0	0	0	0	0	0	0	0	0	0
30	0	0	0	0	0	0	0	0	0	0	0	0	0	0	0	0	20	0	0	0
31	0	0	0	0	0	0	0	0	0	0	0	0	0	20	0	0	0	0	0	0
32	0	0	0	0	20	0	0	0	0	0	0	0	0	0	0	0	0	0	0	0

Position	A	C	D	E	F	G	H	I	K	L	M	N	P	Q	R	S	T	V	W	Y
33	0	0	0	0	0	0	0	0	0	0	0	0	0	0	0	0	0	20	0	0
34	0	0	0	0	0	0	0	0	0	0	0	0	0	0	20	0	0	0	0	0
35	0	0	0	0	20	0	0	0	0	0	0	0	0	0	0	0	0	0	0	0
36	0	0	20	0	0	0	0	0	0	0	0	0	0	0	0	0	0	0	0	0
37	0	0	0	0	0	0	0	0	0	0	0	1	0	0	0	19	0	0	0	0
38	0	0	19	0	0	0	0	0	0	0	0	0	0	0	0	0	0	0	0	1
39	20	0	0	0	0	0	0	0	0	0	0	0	0	0	0	0	0	0	0	0
40	0	0	0	0	0	0	0	0	0	0	0	0	20	0	0	0	0	0	0	0
41	0	0	0	0	0	0	0	0	0	0	0	20	0	0	0	0	0	0	0	0
42	1	0	0	0	0	0	0	0	0	0	0	0	19	0	0	0	0	0	0	0
43	0	0	0	0	0	0	0	0	1	0	0	0	0	0	19	0	0	0	0	0
44	0	0	0	7	0	0	0	0	2	0	10	0	0	0	1	0	0	0	0	0
45	0	0	0	20	0	0	0	0	0	0	0	0	0	0	0	0	0	0	0	0
46	0	0	0	0	0	0	0	0	0	0	0	0	20	0	0	0	0	0	0	0
47	0	0	0	0	0	0	0	0	0	0	0	0	0	0	20	0	0	0	0	0
48	19	0	0	0	0	0	0	0	0	0	0	0	0	0	0	0	0	1	0	0
49	0	0	0	0	0	0	0	0	0	0	0	0	18	1	1	0	0	0	0	0
50	0	0	0	0	0	0	0	0	0	0	0	0	0	0	0	0	0	0	20	0
51	0	0	0	0	0	0	0	20	0	0	0	0	0	0	0	0	0	0	0	0
52	0	0	0	11	0	0	0	0	0	0	0	0	0	9	0	0	0	0	0	0
53	0	0	0	0	0	0	0	0	7	0	0	0	0	13	0	0	0	0	0	0
54	0	0	0	20	0	0	0	0	0	0	0	0	0	0	0	0	0	0	0	0
55	0	0	0	0	0	20	0	0	0	0	0	0	0	0	0	0	0	0	0	0
56	0	0	0	0	0	0	0	0	0	0	0	0	0	20	0	0	0	0	0	0
57	0	0	8	12	0	0	0	0	0	0	0	0	0	0	0	0	0	0	0	0
58	0	0	0	0	0	0	0	0	0	0	0	0	0	0	0	0	0	0	0	20
59	0	0	0	0	0	0	0	0	0	0	0	0	0	0	0	0	0	0	20	0
60	0	0	20	0	0	0	0	0	0	0	0	0	0	0	0	0	0	0	0	0
61	0	0	0	6	0	0	0	0	0	0	0	0	0	0	14	0	0	0	0	0
62	0	0	0	16	0	0	0	0	0	0	0	4	0	0	0	0	0	0	0	0
63	0	0	0	0	0	0	0	0	0	0	0	0	0	0	0	0	20	0	0	0
64	0	0	0	0	0	0	0	0	0	0	0	0	0	10	10	0	0	0	0	0
65	0	0	0	0	0	0	0	10	5	0	0	5	0	0	0	0	0	0	0	0
66	4	0	0	0	0	0	0	0	0	0	0	0	0	1	0	7	0	6	0	2
67	0	0	0	0	0	0	0	0	11	0	7	0	0	0	1	0	0	1	0	0
68	0	0	6	6	0	8	0	0	0	0	0	0	0	0	0	0	0	0	0	0

Position	A	C	D	E	F	G	H	I	K	L	M	N	P	Q	R	S	T	V	W	Y
69	0	0	0	0	0	0	0	0	0	0	0	4	0	0	0	7	8	0	0	1
70	19	0	0	0	0	0	0	0	0	0	0	0	0	0	0	1	0	0	0	0
71	0	0	0	1	0	0	0	0	0	0	0	0	0	19	0	0	0	0	0	0
72	0	0	0	0	0	0	0	5	0	0	0	1	0	0	1	0	13	0	0	0
73	0	0	3	0	6	0	0	0	0	0	0	3	0	0	0	0	0	0	0	8
74	0	0	0	0	0	0	0	0	0	0	0	0	0	0	20	0	0	0	0	0
75	0	0	0	0	0	3	0	0	0	0	0	0	0	0	0	0	0	17	0	0
76	0	0	6	0	0	4	0	0	0	0	0	8	0	0	0	2	0	0	0	0
77	0	0	0	0	0	0	0	0	0	20	0	0	0	0	0	0	0	0	0	0
78	0	0	0	0	0	0	0	0	7	0	0	9	0	0	4	0	0	0	0	0
79	0	0	0	0	0	0	0	1	0	0	0	3	0	0	0	0	16	0	0	0
80	4	0	0	0	0	0	0	0	0	16	0	0	0	0	0	0	0	0	0	0
81	0	0	0	0	0	0	0	0	0	4	0	0	0	0	16	0	0	0	0	0
82	0	0	0	0	0	19	0	0	0	0	0	0	0	0	0	1	0	0	0	0
83	0	0	0	0	0	0	0	0	0	0	0	0	0	0	0	0	0	0	0	20
84	0	0	0	0	0	0	0	0	0	0	0	0	0	0	0	0	0	0	0	20
85	0	0	0	0	0	0	0	0	0	0	0	20	0	0	0	0	0	0	0	0
86	0	0	0	0	0	0	0	0	0	0	0	0	0	20	0	0	0	0	0	0
87	0	0	0	0	0	0	0	0	0	0	0	0	0	0	0	20	0	0	0	0
88	0	0	0	19	0	0	0	0	0	0	0	0	0	0	0	0	0	1	0	0
89	20	0	0	0	0	0	0	0	0	0	0	0	0	0	0	0	0	0	0	0
90	0	0	0	0	0	20	0	0	0	0	0	0	0	0	0	0	0	0	0	0
91	0	0	0	0	0	0	0	0	0	0	0	0	0	0	0	20	0	0	0	0
92	0	0	0	0	0	0	20	0	0	0	0	0	0	0	0	0	0	0	0	0
93	0	0	0	0	0	0	0	0	0	0	0	1	0	0	0	0	19	0	0	0
94	0	0	0	0	3	0	0	7	0	7	0	0	0	0	0	0	0	0	0	3
95	0	0	0	0	0	0	0	0	0	0	0	0	0	20	0	0	0	0	0	0
96	0	0	0	0	0	0	0	3	0	0	0	0	0	0	3	7	0	2	5	0
97	0	0	0	0	0	0	0	0	0	0	20	0	0	0	0	0	0	0	0	0
98	0	0	0	0	2	0	0	0	0	0	0	0	0	0	0	0	0	0	0	18
99	0	0	0	0	0	20	0	0	0	0	0	0	0	0	0	0	0	0	0	0
100	0	20	0	0	0	0	0	0	0	0	0	0	0	0	0	0	0	0	0	0
101	0	0	13	0	0	0	0	0	0	0	0	0	0	0	0	0	0	0	0	7
102	0	0	0	0	0	0	0	0	0	7	2	0	0	0	0	0	0	11	0	0
103	0	0	0	0	0	20	0	0	0	0	0	0	0	0	0	0	0	0	0	0
104	0	0	0	0	0	0	0	0	0	0	0	0	20	0	0	0	0	0	0	0

Position	A	C	D	E	F	G	H	I	K	L	M	N	P	Q	R	S	T	V	W	Y
105	0	0	20	0	0	0	0	0	0	0	0	0	0	0	0	0	0	0	0	0
106	0	0	0	0	0	20	0	0	0	0	0	0	0	0	0	0	0	0	0	0
107	0	0	0	0	0	0	0	0	0	20	0	0	0	0	0	0	0	0	0	0
108	0	0	0	0	6	0	0	0	0	14	0	0	0	0	0	0	0	0	0	0
109	0	0	0	0	0	0	0	0	0	20	0	0	0	0	0	0	0	0	0	0
110	0	0	0	0	0	0	2	0	0	0	0	0	0	0	18	0	0	0	0	0
111	0	0	0	0	0	20	0	0	0	0	0	0	0	0	0	0	0	0	0	0
112	0	0	0	0	0	0	0	0	0	0	0	0	0	0	0	0	0	0	0	20
113	0	0	3	0	0	0	2	0	0	0	0	0	0	0	6	9	0	0	0	0
114	0	0	0	0	0	0	0	0	0	0	0	0	0	20	0	0	0	0	0	0
115	0	0	12	0	6	0	0	0	0	0	0	0	0	0	0	0	0	0	0	2
116	17	0	0	0	0	3	0	0	0	0	0	0	0	0	0	0	0	0	0	0
117	0	0	0	0	0	0	0	0	0	0	0	0	0	0	0	0	0	0	0	20
118	0	0	20	0	0	0	0	0	0	0	0	0	0	0	0	0	0	0	0	0
119	0	0	0	0	0	18	0	0	0	0	0	0	0	0	0	2	0	0	0	0
120	20	0	0	0	0	0	0	0	0	0	0	0	0	0	0	0	0	0	0	0
121	0	0	20	0	0	0	0	0	0	0	0	0	0	0	0	0	0	0	0	0
122	0	0	0	0	0	0	0	0	0	0	0	0	0	0	0	0	0	0	0	20
123	0	0	0	0	0	0	0	13	0	5	0	0	0	0	0	0	0	2	0	0
124	20	0	0	0	0	0	0	0	0	0	0	0	0	0	0	0	0	0	0	0
125	0	0	0	0	0	0	0	0	0	20	0	0	0	0	0	0	0	0	0	0
126	0	0	0	0	0	0	0	0	0	0	0	20	0	0	0	0	0	0	0	0
127	0	0	0	20	0	0	0	0	0	0	0	0	0	0	0	0	0	0	0	0
128	0	0	20	0	0	0	0	0	0	0	0	0	0	0	0	0	0	0	0	0
129	0	0	0	0	0	0	0	0	0	20	0	0	0	0	0	0	0	0	0	0
130	0	0	0	0	0	0	0	0	0	0	0	0	0	0	20	0	0	0	0	0
131	0	0	0	0	0	0	0	0	0	0	0	0	0	0	0	20	0	0	0	0
132	0	0	0	0	0	0	0	0	0	0	0	0	0	0	0	0	0	0	20	0
133	0	0	0	0	0	0	0	0	0	0	0	0	0	0	0	0	20	0	0	0
134	20	0	0	0	0	0	0	0	0	0	0	0	0	0	0	0	0	0	0	0
135	20	0	0	0	0	0	0	0	0	0	0	0	0	0	0	0	0	0	0	0
136	0	0	20	0	0	0	0	0	0	0	0	0	0	0	0	0	0	0	0	0
137	0	0	0	0	0	0	0	0	0	0	7	0	0	0	0	0	13	0	0	0
138	20	0	0	0	0	0	0	0	0	0	0	0	0	0	0	0	0	0	0	0
139	20	0	0	0	0	0	0	0	0	0	0	0	0	0	0	0	0	0	0	0
140	0	0	0	0	0	0	0	0	0	0	0	0	0	20	0	0	0	0	0	0

Position	A	C	D	E	F	G	H	I	K	L	M	N	P	Q	R	S	T	V	W	Y
141	0	0	0	0	0	0	0	20	0	0	0	0	0	0	0	0	0	0	0	0
142	0	0	0	0	0	0	0	0	0	0	0	0	0	0	0	7	13	0	0	0
143	0	0	0	0	0	0	0	0	20	0	0	0	0	0	0	0	0	0	0	0
144	0	0	0	0	0	0	0	0	0	0	0	0	0	0	20	0	0	0	0	0
145	0	0	0	0	0	0	0	0	20	0	0	0	0	0	0	0	0	0	0	0
146	0	0	0	0	2	0	0	0	0	0	0	0	0	0	5	0	0	0	13	0
147	0	0	2	18	0	0	0	0	0	0	0	0	0	0	0	0	0	0	0	0
148	18	0	0	0	0	0	0	0	0	0	0	0	0	0	0	0	2	0	0	0
149	20	0	0	0	0	0	0	0	0	0	0	0	0	0	0	0	0	0	0	0
150	0	0	13	0	0	0	0	0	0	0	0	7	0	0	0	0	0	0	0	0
151	6	0	0	7	0	0	0	0	0	0	0	0	0	0	0	0	0	7	0	0
152	20	0	0	0	0	0	0	0	0	0	0	0	0	0	0	0	0	0	0	0
153	0	0	0	20	0	0	0	0	0	0	0	0	0	0	0	0	0	0	0	0
154	0	0	0	0	0	1	2	0	0	0	0	0	0	10	7	0	0	0	0	0
155	0	0	0	6	0	0	0	0	0	0	6	0	0	0	3	0	0	0	5	0
156	0	0	0	0	0	0	0	0	0	0	0	0	0	0	20	0	0	0	0	0
157	0	0	0	0	0	0	0	0	0	0	0	0	0	0	0	20	0	0	0	0
158	0	0	0	0	0	0	0	0	0	0	0	0	0	0	0	0	0	0	0	20
159	0	0	0	0	0	0	0	0	0	20	0	0	0	0	0	0	0	0	0	0
160	0	0	0	2	0	0	0	0	0	0	0	0	0	18	0	0	0	0	0	0
161	0	0	0	0	0	20	0	0	0	0	0	0	0	0	0	0	0	0	0	0
162	1	0	0	0	0	0	0	0	0	10	0	0	0	0	5	0	4	0	0	0
163	0	20	0	0	0	0	0	0	0	0	0	0	0	0	0	0	0	0	0	0
164	0	0	0	0	0	0	0	0	0	0	0	0	0	0	0	0	0	20	0	0
165	0	0	0	20	0	0	0	0	0	0	0	0	0	0	0	0	0	0	0	0
166	0	0	0	0	0	3	0	0	0	0	0	0	0	0	0	4	0	0	13	0
167	0	0	0	0	0	0	0	0	0	20	0	0	0	0	0	0	0	0	0	0
168	0	0	0	0	0	0	0	0	0	0	0	0	0	13	7	0	0	0	0	0
169	0	0	0	2	0	0	0	0	15	0	0	0	0	0	3	0	0	0	0	0
170	0	0	0	0	0	0	0	0	0	0	0	0	0	0	0	0	0	0	0	20
171	0	0	0	0	0	0	0	0	0	20	0	0	0	0	0	0	0	0	0	0
172	0	0	0	16	0	0	0	0	0	0	0	0	0	4	0	0	0	0	0	0
173	0	0	0	0	0	0	0	0	0	0	20	0	0	0	0	0	0	0	0	0
174	0	0	0	0	0	20	0	0	0	0	0	0	0	0	0	0	0	0	0	0
175	0	0	0	1	0	0	0	0	19	0	0	0	0	0	0	0	0	0	0	0
176	0	0	20	0	0	0	0	0	0	0	0	0	0	0	0	0	0	0	0	0

Position	A	C	D	E	F	G	H	I	K	L	M	N	P	Q	R	S	T	V	W	Y
177	0	0	0	0	0	0	0	0	0	0	0	0	0	0	0	0	20	0	0	0
178	0	0	0	0	0	0	0	0	0	20	0	0	0	0	0	0	0	0	0	0
179	0	0	0	0	0	0	0	0	0	0	0	0	0	20	0	0	0	0	0	0
180	0	0	0	0	0	0	0	0	0	0	0	0	0	0	20	0	0	0	0	0
181	20	0	0	0	0	0	0	0	0	0	0	0	0	0	0	0	0	0	0	0

Supplementary Table S10. Frequency of amino acids for each residue constituting the peptide-binding region of 20 SLA-3 alleles from different allelic groups according to the 2-digit nomenclature

Position	A	C	D	E	F	G	H	I	K	L	M	N	P	Q	R	S	T	V	W	Y
1	0	0	0	0	0	0	0	0	0	0	0	0	20	0	0	0	0	0	0	0
2	0	0	0	0	0	0	20	0	0	0	0	0	0	0	0	0	0	0	0	0
3	0	0	0	0	0	0	0	0	0	0	0	0	0	0	0	20	0	0	0	0
4	0	0	0	0	0	0	0	0	0	20	0	0	0	0	0	0	0	0	0	0
5	0	0	0	0	0	0	0	0	0	0	0	0	0	0	18	2	0	0	0	0
6	0	0	0	0	0	0	0	0	0	0	0	0	0	0	0	0	0	0	0	20
7	0	0	0	0	20	0	0	0	0	0	0	0	0	0	0	0	0	0	0	0
8	0	0	16	0	2	0	2	0	0	0	0	0	0	0	0	0	0	0	0	0
9	0	0	0	0	0	0	0	0	0	0	0	0	0	0	0	0	20	0	0	0
10	20	0	0	0	0	0	0	0	0	0	0	0	0	0	0	0	0	0	0	0
11	0	0	0	0	0	0	0	0	0	0	0	0	0	0	0	0	0	20	0	0
12	0	0	0	0	1	0	0	0	0	0	0	0	0	0	0	19	0	0	0	0
13	0	0	0	0	0	0	0	0	0	0	0	0	0	0	20	0	0	0	0	0
14	0	0	0	0	0	0	0	0	0	0	0	0	20	0	0	0	0	0	0	0
15	0	0	20	0	0	0	0	0	0	0	0	0	0	0	0	0	0	0	0	0
16	0	0	0	0	0	0	0	0	0	2	0	0	0	0	18	0	0	0	0	0
17	0	0	0	0	0	19	0	0	0	0	0	0	0	0	1	0	0	0	0	0
18	0	0	0	0	0	0	0	0	20	0	0	0	0	0	0	0	0	0	0	0
19	0	0	0	0	0	0	0	0	0	0	0	0	19	0	0	0	1	0	0	0
20	0	0	0	0	0	0	0	0	0	0	0	0	0	0	20	0	0	0	0	0
21	0	0	0	0	20	0	0	0	0	0	0	0	0	0	0	0	0	0	0	0
22	0	0	0	0	0	0	0	20	0	0	0	0	0	0	0	0	0	0	0	0
23	2	0	0	5	0	0	0	0	0	0	0	0	0	0	0	13	0	0	0	0
24	0	0	0	0	0	0	0	0	0	0	0	0	0	0	0	0	0	20	0	0
25	0	0	0	0	0	20	0	0	0	0	0	0	0	0	0	0	0	0	0	0
26	0	0	0	0	0	0	0	0	0	0	0	0	0	0	0	0	0	0	0	20
27	0	0	0	0	0	0	0	0	0	0	0	0	0	0	0	0	0	20	0	0
28	0	0	20	0	0	0	0	0	0	0	0	0	0	0	0	0	0	0	0	0
29	0	0	20	0	0	0	0	0	0	0	0	0	0	0	0	0	0	0	0	0
30	0	0	0	0	0	0	0	0	0	0	0	0	0	0	0	0	20	0	0	0
31	0	0	0	0	0	0	0	0	0	0	0	0	0	20	0	0	0	0	0	0
32	0	0	0	0	20	0	0	0	0	0	0	0	0	0	0	0	0	0	0	0

Position	A	C	D	E	F	G	H	I	K	L	M	N	P	Q	R	S	T	V	W	Y
33	0	0	0	0	0	0	0	0	0	0	0	0	0	0	0	0	0	20	0	0
34	0	0	0	0	0	0	0	0	0	0	0	0	0	0	19	0	0	0	1	0
35	0	0	0	0	20	0	0	0	0	0	0	0	0	0	0	0	0	0	0	0
36	0	0	20	0	0	0	0	0	0	0	0	0	0	0	0	0	0	0	0	0
37	0	0	0	0	0	0	0	0	0	0	0	0	0	0	0	20	0	0	0	0
38	0	0	17	0	0	0	0	0	0	0	0	0	0	0	0	0	0	0	0	3
39	20	0	0	0	0	0	0	0	0	0	0	0	0	0	0	0	0	0	0	0
40	0	0	0	0	0	0	0	0	0	1	0	0	19	0	0	0	0	0	0	0
41	0	0	0	0	0	0	0	0	0	0	0	20	0	0	0	0	0	0	0	0
42	0	0	0	0	0	0	0	0	0	0	0	0	20	0	0	0	0	0	0	0
43	0	0	0	0	0	0	0	0	0	0	0	0	0	0	20	0	0	0	0	0
44	0	0	0	0	0	0	0	0	0	0	20	0	0	0	0	0	0	0	0	0
45	0	0	0	20	0	0	0	0	0	0	0	0	0	0	0	0	0	0	0	0
46	0	0	0	0	0	0	0	0	0	0	0	0	19	0	0	1	0	0	0	0
47	0	0	0	0	0	0	0	0	0	0	0	0	0	0	20	0	0	0	0	0
48	16	0	0	0	0	0	0	0	0	0	0	0	0	0	0	0	0	4	0	0
49	0	0	0	0	0	0	0	0	0	0	0	0	14	6	0	0	0	0	0	0
50	0	0	0	0	0	0	0	0	0	0	0	0	0	0	0	0	0	0	20	0
51	0	0	0	0	0	0	0	20	0	0	0	0	0	0	0	0	0	0	0	0
52	0	0	0	8	0	0	0	0	2	0	0	0	0	10	0	0	0	0	0	0
53	0	0	0	0	0	0	0	0	4	0	0	0	0	16	0	0	0	0	0	0
54	1	0	0	17	0	0	0	0	2	0	0	0	0	0	0	0	0	0	0	0
55	0	0	0	2	0	18	0	0	0	0	0	0	0	0	0	0	0	0	0	0
56	0	0	0	0	0	0	0	0	0	0	0	0	0	20	0	0	0	0	0	0
57	0	0	4	12	0	0	0	0	4	0	0	0	0	0	0	0	0	0	0	0
58	0	0	0	0	0	0	0	0	0	0	0	0	0	0	0	0	0	0	1	0
59	0	0	0	0	0	0	0	0	0	0	0	0	0	1	0	0	0	0	0	0
60	0	0	0	1	0	0	0	0	0	0	0	0	0	0	0	0	0	0	0	0
61	0	0	0	0	0	0	0	0	0	0	0	0	0	0	0	0	0	0	0	20
62	0	0	0	0	0	0	0	0	0	0	0	0	0	0	0	0	0	0	20	0
63	0	0	20	0	0	0	0	0	0	0	0	0	0	0	0	0	0	0	0	0
64	0	0	0	8	0	0	0	0	2	0	0	0	0	1	9	0	0	0	0	0
65	0	0	0	18	0	0	0	0	0	0	0	1	0	1	0	0	0	0	0	0
66	0	0	0	0	0	0	0	0	0	0	0	0	0	0	0	0	20	0	0	0
67	0	0	0	1	0	0	0	0	0	0	0	0	0	12	7	0	0	0	0	0
68	0	0	0	0	0	0	0	5	2	0	0	13	0	0	0	0	0	0	0	0

Position	A	C	D	E	F	G	H	I	K	L	M	N	P	Q	R	S	T	V	W	Y
69	12	0	0	0	0	0	0	0	0	0	0	0	0	1	0	7	0	0	0	0
70	0	0	0	0	0	0	0	0	7	0	8	0	0	0	1	0	0	4	0	0
71	0	0	8	0	0	12	0	0	0	0	0	0	0	0	0	0	0	0	0	0
72	0	0	0	0	0	0	0	0	0	0	0	7	0	0	0	12	1	0	0	0
73	19	0	0	0	0	0	0	0	0	0	0	0	0	0	0	1	0	0	0	0
74	0	0	0	0	0	0	0	0	0	0	0	0	0	20	0	0	0	0	0	0
75	0	0	0	0	0	0	0	0	0	0	0	0	0	0	6	0	14	0	0	0
76	0	0	0	0	13	0	0	0	0	0	0	0	0	0	0	0	0	0	0	7
77	0	0	0	0	0	0	0	0	0	0	0	0	0	0	20	0	0	0	0	0
78	0	0	0	0	0	0	0	0	0	0	0	0	0	0	0	0	0	20	0	0
79	0	0	0	0	0	0	0	0	0	0	0	17	0	0	0	3	0	0	0	0
80	0	0	0	0	0	0	0	0	0	20	0	0	0	0	0	0	0	0	0	0
81	0	0	0	0	0	0	0	0	9	0	0	11	0	0	0	0	0	0	0	0
82	0	0	0	0	0	0	0	0	0	0	0	12	0	0	0	0	8	0	0	0
83	0	0	0	0	0	0	0	0	0	20	0	0	0	0	0	0	0	0	0	0
84	0	0	0	0	0	0	0	0	0	0	0	0	0	0	20	0	0	0	0	0
85	0	0	0	0	0	20	0	0	0	0	0	0	0	0	0	0	0	0	0	0
86	0	0	0	0	0	0	0	0	0	0	0	0	0	0	0	0	0	0	0	20
87	0	0	0	0	0	0	0	0	0	0	0	0	0	0	0	0	0	0	0	20
88	0	0	0	0	0	0	0	0	0	0	0	19	0	0	0	1	0	0	0	0
89	0	0	0	0	0	0	0	0	0	0	0	0	0	20	0	0	0	0	0	0
90	0	0	0	0	0	0	0	0	0	0	0	0	0	0	0	20	0	0	0	0
91	0	0	0	20	0	0	0	0	0	0	0	0	0	0	0	0	0	0	0	0
92	20	0	0	0	0	0	0	0	0	0	0	0	0	0	0	0	0	0	0	0
93	0	0	0	0	0	20	0	0	0	0	0	0	0	0	0	0	0	0	0	0
94	0	0	0	0	0	0	0	0	0	0	0	0	0	0	0	20	0	0	0	0
95	0	0	0	0	0	0	20	0	0	0	0	0	0	0	0	0	0	0	0	0
96	0	0	0	0	0	0	0	0	0	0	0	0	0	0	0	0	20	0	0	0
97	0	0	0	0	1	0	0	8	0	11	0	0	0	0	0	0	0	0	0	0
98	0	0	0	0	0	0	0	0	0	0	0	0	0	20	0	0	0	0	0	0
99	0	0	0	0	0	0	0	0	0	0	0	0	0	0	3	12	1	4	0	0
100	0	0	0	0	0	0	0	0	0	0	20	0	0	0	0	0	0	0	0	0
101	0	0	0	0	0	0	0	0	0	0	0	0	0	0	0	0	0	0	0	20
102	0	0	0	0	0	20	0	0	0	0	0	0	0	0	0	0	0	0	0	0
103	0	20	0	0	0	0	0	0	0	0	0	0	0	0	0	0	0	0	0	0
104	0	0	17	0	0	0	0	0	0	0	0	0	0	0	0	0	0	0	0	3

Position	A	C	D	E	F	G	H	I	K	L	M	N	P	Q	R	S	T	V	W	Y
105	0	0	0	0	0	0	0	0	0	3	0	0	0	0	0	0	0	17	0	0
106	0	0	0	0	0	20	0	0	0	0	0	0	0	0	0	0	0	0	0	0
107	0	0	0	0	0	0	0	0	0	0	0	0	20	0	0	0	0	0	0	0
108	0	0	20	0	0	0	0	0	0	0	0	0	0	0	0	0	0	0	0	0
109	0	0	0	0	0	18	0	0	0	0	0	0	0	0	0	0	0	0	2	0
110	0	0	0	0	2	0	0	0	0	18	0	0	0	0	0	0	0	0	0	0
111	0	0	0	0	15	0	0	0	0	5	0	0	0	0	0	0	0	0	0	0
112	0	0	0	0	0	0	0	0	0	20	0	0	0	0	0	0	0	0	0	0
113	0	0	0	0	0	0	12	0	0	0	0	0	0	0	8	0	0	0	0	0
114	0	0	0	0	0	20	0	0	0	0	0	0	0	0	0	0	0	0	0	0
115	0	0	0	0	0	0	0	0	0	0	0	0	0	0	0	0	0	0	0	20
116	0	0	0	0	0	0	13	0	0	0	0	0	0	0	0	7	0	0	0	0
117	0	0	0	0	0	0	0	0	0	0	0	0	0	20	0	0	0	0	0	0
118	0	0	14	0	2	0	0	0	0	0	0	0	0	0	0	0	0	0	0	4
119	19	0	0	0	0	1	0	0	0	0	0	0	0	0	0	0	0	0	0	0
120	0	0	0	0	0	0	0	0	0	0	0	0	0	0	0	0	0	0	0	20
121	0	0	20	0	0	0	0	0	0	0	0	0	0	0	0	0	0	0	0	0
122	0	0	0	0	0	20	0	0	0	0	0	0	0	0	0	0	0	0	0	0
123	20	0	0	0	0	0	0	0	0	0	0	0	0	0	0	0	0	0	0	0
124	0	0	20	0	0	0	0	0	0	0	0	0	0	0	0	0	0	0	0	0
125	0	0	0	0	0	0	0	0	0	0	0	0	0	0	0	0	0	0	0	20
126	0	0	0	0	0	0	0	10	0	9	0	0	0	0	0	0	0	1	0	0
127	20	0	0	0	0	0	0	0	0	0	0	0	0	0	0	0	0	0	0	0
128	0	0	0	0	0	0	0	0	0	20	0	0	0	0	0	0	0	0	0	0
129	0	0	0	0	0	0	0	0	0	0	0	20	0	0	0	0	0	0	0	0
130	0	0	0	20	0	0	0	0	0	0	0	0	0	0	0	0	0	0	0	0
131	0	0	20	0	0	0	0	0	0	0	0	0	0	0	0	0	0	0	0	0
132	0	0	0	0	0	0	0	0	0	20	0	0	0	0	0	0	0	0	0	0
133	0	1	0	0	0	0	0	0	0	0	0	0	0	0	19	0	0	0	0	0
134	0	0	0	0	0	0	0	0	0	0	0	0	0	0	0	20	0	0	0	0
135	0	0	0	0	0	0	0	0	0	0	0	0	0	0	0	0	0	0	20	0
136	0	0	0	0	0	0	0	0	0	0	0	0	0	0	0	0	20	0	0	0
137	20	0	0	0	0	0	0	0	0	0	0	0	0	0	0	0	0	0	0	0
138	19	0	0	1	0	0	0	0	0	0	0	0	0	0	0	0	0	0	0	0
139	0	0	20	0	0	0	0	0	0	0	0	0	0	0	0	0	0	0	0	0
140	0	0	0	0	0	0	0	0	0	0	1	0	0	0	0	0	19	0	0	0

Position	A	C	D	E	F	G	H	I	K	L	M	N	P	Q	R	S	T	V	W	Y
141	20	0	0	0	0	0	0	0	0	0	0	0	0	0	0	0	0	0	0	0
142	20	0	0	0	0	0	0	0	0	0	0	0	0	0	0	0	0	0	0	0
143	0	0	0	0	0	0	0	0	0	0	0	0	0	20	0	0	0	0	0	0
144	0	0	0	0	0	0	0	20	0	0	0	0	0	0	0	0	0	0	0	0
145	2	0	0	0	0	0	0	0	0	0	0	0	0	0	0	8	10	0	0	0
146	0	0	0	0	0	0	0	0	20	0	0	0	0	0	0	0	0	0	0	0
147	0	0	0	0	0	0	0	0	0	0	0	0	0	0	20	0	0	0	0	0
148	0	0	0	0	0	0	0	0	20	0	0	0	0	0	0	0	0	0	0	0
149	0	0	0	0	0	0	0	0	0	0	0	0	0	1	9	0	0	0	10	0
150	0	0	0	20	0	0	0	0	0	0	0	0	0	0	0	0	0	0	0	0
151	19	0	0	1	0	0	0	0	0	0	0	0	0	0	0	0	0	0	0	0
152	20	0	0	0	0	0	0	0	0	0	0	0	0	0	0	0	0	0	0	0
153	0	0	19	0	0	0	0	0	0	0	0	1	0	0	0	0	0	0	0	0
154	10	0	0	2	0	0	0	0	0	0	0	0	0	0	0	0	0	8	0	0
155	17	0	0	0	0	0	0	0	0	0	0	0	0	0	0	0	3	0	0	0
156	0	0	0	20	0	0	0	0	0	0	0	0	0	0	0	0	0	0	0	0
157	0	0	0	0	0	0	1	0	0	0	0	0	0	15	4	0	0	0	0	0
158	0	0	0	0	0	0	0	0	0	0	8	0	0	0	3	0	0	0	9	0
159	0	0	0	0	0	0	0	0	0	0	0	0	0	0	20	0	0	0	0	0
160	0	0	0	0	0	0	0	0	0	0	0	0	0	0	0	20	0	0	0	0
161	0	0	0	0	0	0	0	0	0	0	0	0	0	0	0	0	0	0	0	20
162	0	0	0	0	0	0	0	0	0	20	0	0	0	0	0	0	0	0	0	0
163	0	0	0	19	0	0	0	0	0	0	0	0	0	1	0	0	0	0	0	0
164	0	0	0	0	0	20	0	0	0	0	0	0	0	0	0	0	0	0	0	0
165	19	0	0	0	0	0	0	0	0	0	0	0	0	0	1	0	0	0	0	0
166	0	20	0	0	0	0	0	0	0	0	0	0	0	0	0	0	0	0	0	0
167	0	0	0	0	0	0	0	0	0	0	0	0	0	0	0	0	0	20	0	0
168	0	0	0	19	0	0	0	0	0	0	0	0	0	0	0	0	0	1	0	0
169	0	0	0	0	0	7	0	0	0	0	0	0	0	0	0	2	0	0	11	0
170	0	0	0	0	0	0	0	0	0	20	0	0	0	0	0	0	0	0	0	0
171	0	0	0	0	0	0	0	0	0	1	0	0	0	16	3	0	0	0	0	0
172	0	0	0	3	0	0	0	0	17	0	0	0	0	0	0	0	0	0	0	0
173	0	0	0	0	0	0	0	0	0	0	0	0	0	0	0	0	0	0	0	20
174	0	0	0	0	0	0	0	0	0	20	0	0	0	0	0	0	0	0	0	0
175	0	0	3	13	0	0	0	0	0	0	0	0	0	4	0	0	0	0	0	0
176	0	0	0	0	0	0	0	0	0	3	17	0	0	0	0	0	0	0	0	0

Position	A	C	D	E	F	G	H	I	K	L	M	N	P	Q	R	S	T	V	W	Y
177	0	0	0	0	0	20	0	0	0	0	0	0	0	0	0	0	0	0	0	0
178	0	0	0	4	0	0	0	0	12	0	0	4	0	0	0	0	0	0	0	0
179	0	0	16	0	0	0	0	0	0	0	0	4	0	0	0	0	0	0	0	0
180	0	0	0	0	0	0	0	0	0	0	0	0	0	0	0	0	20	0	0	0
181	0	0	0	0	0	0	0	0	0	20	0	0	0	0	0	0	0	0	0	0
182	0	0	0	0	0	0	0	0	0	0	0	0	0	20	0	0	0	0	0	0
183	0	0	0	0	0	0	1	0	0	0	0	0	0	0	19	0	0	0	0	0
184	18	0	0	0	0	0	0	0	0	0	0	0	0	0	0	2	0	0	0	0

Supplementary Table S11. Frequency of amino acids for each residue constituting the peptide-binding region of 20 BoLA-2 alleles from different allelic groups according to the 2-digit nomenclature

Position	A	C	D	E	F	G	H	I	K	L	M	N	P	Q	R	S	T	V	W	Y
1	0	0	0	0	0	0	0	0	0	0	0	0	0	0	0	20	0	0	0	0
2	0	0	0	0	0	0	20	0	0	0	0	0	0	0	0	0	0	0	0	0
3	0	0	0	0	0	0	0	0	0	0	0	0	0	0	0	20	0	0	0	0
4	0	0	0	0	0	0	0	0	0	17	3	0	0	0	0	0	0	0	0	0
5	0	0	0	0	0	0	0	0	1	0	0	0	0	0	19	0	0	0	0	0
6	0	0	0	0	0	0	0	0	0	0	0	0	0	0	0	0	0	0	0	20
7	0	0	0	0	20	0	0	0	0	0	0	0	0	0	0	0	0	0	0	0
8	0	0	0	0	0	0	1	0	0	1	0	0	0	0	0	7	0	0	0	11
9	0	0	0	0	0	0	0	0	0	0	0	0	0	0	0	0	20	0	0	0
10	13	0	0	0	0	7	0	0	0	0	0	0	0	0	0	0	0	0	0	0
11	0	0	0	0	0	0	0	0	0	0	0	0	0	0	0	0	0	20	0	0
12	0	0	0	0	0	0	0	0	0	0	0	0	0	0	0	20	0	0	0	0
13	0	0	0	0	0	0	0	0	0	0	0	0	0	0	20	0	0	0	0	0
14	0	0	0	0	0	0	0	0	0	0	0	0	20	0	0	0	0	0	0	0
15	0	0	0	0	0	20	0	0	0	0	0	0	0	0	0	0	0	0	0	0
16	0	0	0	0	3	0	0	0	0	17	0	0	0	0	0	0	0	0	0	0
17	0	0	0	0	0	20	0	0	0	0	0	0	0	0	0	0	0	0	0	0
18	0	0	0	20	0	0	0	0	0	0	0	0	0	0	0	0	0	0	0	0
19	0	0	0	0	0	0	0	0	0	0	0	0	20	0	0	0	0	0	0	0
20	0	0	0	0	0	0	0	0	0	0	0	0	0	0	19	1	0	0	0	0
21	0	0	0	0	17	0	0	0	0	0	0	0	0	0	0	0	0	0	0	3
22	0	0	0	0	0	0	0	17	0	3	0	0	0	0	0	0	0	0	0	0
23	10	0	0	3	0	0	0	4	0	0	0	0	0	0	0	3	0	0	0	0
24	0	0	0	0	0	0	0	0	0	0	0	0	0	0	0	0	0	20	0	0
25	0	0	0	0	0	20	0	0	0	0	0	0	0	0	0	0	0	0	0	0
26	0	0	0	0	0	0	0	0	0	0	0	0	0	0	0	0	0	0	0	20
27	0	0	0	0	0	0	0	0	0	0	0	0	0	0	0	0	0	20	0	0
28	0	0	20	0	0	0	0	0	0	0	0	0	0	0	0	0	0	0	0	0
29	0	0	20	0	0	0	0	0	0	0	0	0	0	0	0	0	0	0	0	0
30	0	0	0	0	0	0	0	0	0	0	0	0	0	0	0	0	20	0	0	0
31	0	0	0	0	0	0	0	0	0	0	0	0	0	20	0	0	0	0	0	0
32	0	0	0	0	20	0	0	0	0	0	0	0	0	0	0	0	0	0	0	0

Position	A	C	D	E	F	G	H	I	K	L	M	N	P	Q	R	S	T	V	W	Y
33	0	0	0	0	0	0	0	0	0	0	0	0	0	0	0	0	5	15	0	0
34	0	0	0	0	0	0	0	0	0	0	0	0	0	0	19	0	0	0	1	0
35	0	0	0	0	20	0	0	0	0	0	0	0	0	0	0	0	0	0	0	0
36	0	0	20	0	0	0	0	0	0	0	0	0	0	0	0	0	0	0	0	0
37	0	0	0	0	0	0	0	0	0	0	0	0	0	0	0	20	0	0	0	0
38	0	0	16	0	0	0	0	0	0	0	0	4	0	0	0	0	0	0	0	0
39	14	0	0	0	0	0	0	0	0	0	0	0	0	0	0	2	4	0	0	0
40	3	0	0	0	0	0	0	0	0	0	0	0	17	0	0	0	0	0	0	0
41	0	0	4	0	0	0	0	0	0	0	0	16	0	0	0	0	0	0	0	0
42	0	0	0	0	0	0	0	0	0	0	0	0	20	0	0	0	0	0	0	0
43	0	0	0	0	0	0	0	0	0	0	0	0	0	0	20	0	0	0	0	0
44	0	0	1	8	0	0	0	0	0	0	7	0	0	0	0	0	4	0	0	0
45	0	0	0	20	0	0	0	0	0	0	0	0	0	0	0	0	0	0	0	0
46	0	0	0	0	0	0	0	0	0	0	0	0	20	0	0	0	0	0	0	0
47	0	0	0	0	0	0	0	0	0	0	0	0	0	0	20	0	0	0	0	0
48	13	0	0	0	0	0	0	0	0	0	0	0	0	0	0	0	0	7	0	0
49	0	0	0	0	0	0	0	0	0	0	0	0	10	0	10	0	0	0	0	0
50	0	0	0	0	0	0	0	0	0	0	0	0	0	0	0	0	0	0	20	0
51	0	0	0	0	0	0	0	0	0	0	11	0	0	0	0	0	0	9	0	0
52	0	0	0	20	0	0	0	0	0	0	0	0	0	0	0	0	0	0	0	0
53	0	0	0	0	0	0	0	0	1	0	0	0	0	19	0	0	0	0	0	0
54	0	0	0	20	0	0	0	0	0	0	0	0	0	0	0	0	0	0	0	0
55	0	0	0	0	0	20	0	0	0	0	0	0	0	0	0	0	0	0	0	0
56	0	0	0	0	0	0	0	0	0	5	0	0	15	0	0	0	0	0	0	0
57	0	0	0	20	0	0	0	0	0	0	0	0	0	0	0	0	0	0	0	0
58	0	0	0	0	0	0	0	0	0	0	0	0	0	0	0	0	0	0	0	20
59	0	0	0	0	0	0	0	0	0	0	0	0	0	0	0	0	0	0	20	0
60	0	0	20	0	0	0	0	0	0	0	0	0	0	0	0	0	0	0	0	0
61	0	0	0	2	0	0	0	0	0	0	0	0	0	4	14	0	0	0	0	0
62	0	0	0	10	0	0	0	0	0	0	0	8	0	2	0	0	0	0	0	0
63	0	0	0	0	0	0	0	0	0	0	0	0	0	0	0	0	20	0	0	0
64	0	0	0	0	0	0	0	0	0	0	0	0	0	3	17	0	0	0	0	0
65	0	0	0	0	0	0	0	11	0	0	0	6	0	0	3	0	0	0	0	0
66	4	0	0	0	1	0	0	0	0	2	0	0	0	1	0	3	0	2	0	7
67	0	0	0	0	0	0	0	0	20	0	0	0	0	0	0	0	0	0	0	0
68	0	0	12	3	0	5	0	0	0	0	0	0	0	0	0	0	0	0	0	0

Position	A	C	D	E	F	G	H	I	K	L	M	N	P	Q	R	S	T	V	W	Y
69	5	0	0	0	0	0	0	0	0	0	0	4	0	0	0	0	11	0	0	0
70	19	0	0	0	0	0	0	0	0	0	0	0	0	0	0	0	1	0	0	0
71	0	0	0	0	0	0	0	0	0	0	0	0	0	20	0	0	0	0	0	0
72	0	0	0	0	0	0	0	2	0	0	0	1	0	0	0	2	15	0	0	0
73	0	0	0	0	20	0	0	0	0	0	0	0	0	0	0	0	0	0	0	0
74	0	0	0	0	0	0	0	0	0	0	0	0	0	0	20	0	0	0	0	0
75	6	0	0	0	0	0	0	0	0	0	0	0	0	0	0	0	0	14	0	0
76	0	0	4	0	0	2	0	0	0	0	0	9	0	0	0	1	0	0	0	4
77	0	0	0	0	0	0	0	0	0	20	0	0	0	0	0	0	0	0	0	0
78	0	0	0	0	0	0	0	0	0	0	0	20	0	0	0	0	0	0	0	0
79	1	0	0	0	0	0	0	0	0	0	0	1	0	0	0	0	18	0	0	0
80	3	0	0	0	0	0	0	0	0	17	0	0	0	0	0	0	0	0	0	0
81	0	0	0	0	0	0	0	0	0	5	0	0	0	0	15	0	0	0	0	0
82	0	0	0	0	0	20	0	0	0	0	0	0	0	0	0	0	0	0	0	0
83	0	0	0	0	0	0	0	0	0	0	0	0	0	0	0	0	0	0	0	20
84	0	0	0	0	0	0	0	0	0	0	0	0	0	0	0	0	0	0	0	20
85	0	0	0	0	0	0	0	0	0	0	0	20	0	0	0	0	0	0	0	0
86	0	0	0	0	0	0	0	0	0	0	0	0	0	20	0	0	0	0	0	0
87	0	0	0	0	0	0	0	0	0	0	0	0	0	0	0	20	0	0	0	0
88	0	0	0	20	0	0	0	0	0	0	0	0	0	0	0	0	0	0	0	0
89	19	0	0	0	0	0	0	0	0	0	0	0	0	0	0	0	1	0	0	0
90	0	0	0	0	0	20	0	0	0	0	0	0	0	0	0	0	0	0	0	0
91	0	0	0	0	0	0	0	0	0	0	0	0	0	0	0	20	0	0	0	0
92	0	0	0	0	0	0	20	0	0	0	0	0	0	0	0	0	0	0	0	0
93	0	0	0	0	0	0	0	0	0	0	0	3	0	0	0	0	17	0	0	0
94	0	0	0	0	3	0	0	7	0	9	0	0	0	0	0	0	0	1	0	0
95	0	0	0	0	0	0	0	0	0	0	0	0	0	20	0	0	0	0	0	0
96	2	0	0	5	0	0	0	0	0	3	0	0	0	0	0	0	0	0	10	0
97	0	0	0	0	0	0	0	0	0	0	20	0	0	0	0	0	0	0	0	0
98	0	0	0	0	0	0	0	0	0	0	0	0	0	0	0	6	0	0	0	14
99	0	0	0	0	0	20	0	0	0	0	0	0	0	0	0	0	0	0	0	0
100	0	20	0	0	0	0	0	0	0	0	0	0	0	0	0	0	0	0	0	0
101	0	0	14	0	0	0	0	0	0	0	0	0	0	0	0	0	0	0	0	6
102	0	0	0	0	0	0	0	0	0	0	0	0	0	0	0	0	0	20	0	0
103	0	0	0	0	0	20	0	0	0	0	0	0	0	0	0	0	0	0	0	0
104	0	0	0	0	0	0	0	0	0	0	0	0	20	0	0	0	0	0	0	0

Position	A	C	D	E	F	G	H	I	K	L	M	N	P	Q	R	S	T	V	W	Y
105	0	0	19	0	0	1	0	0	0	0	0	0	0	0	0	0	0	0	0	0
106	0	0	0	0	0	20	0	0	0	0	0	0	0	0	0	0	0	0	0	0
107	0	0	0	0	0	0	0	0	0	0	0	0	0	0	19	0	0	0	0	0
108	0	0	0	0	6	0	0	0	0	14	0	0	0	0	0	0	0	0	0	0
109	0	0	0	0	0	0	0	0	0	18	0	0	0	0	2	0	0	0	0	0
110	0	0	0	0	0	1	0	0	0	1	0	0	0	0	17	1	0	0	0	0
111	0	0	0	0	0	20	0	0	0	0	0	0	0	0	0	0	0	0	0	0
112	0	0	0	0	13	0	0	0	0	0	0	0	0	0	0	0	0	0	0	7
113	0	0	1	1	0	0	0	0	0	0	7	0	0	0	5	0	1	0	5	0
114	0	0	0	0	0	0	0	0	0	0	0	0	0	20	0	0	0	0	0	0
115	0	0	10	0	9	0	0	0	0	0	0	0	0	0	0	0	0	0	0	1
116	12	0	0	0	0	8	0	0	0	0	0	0	0	0	0	0	0	0	0	0
117	0	0	0	0	0	0	0	0	0	0	0	0	0	0	0	0	0	0	0	20
118	0	0	19	1	0	0	0	0	0	0	0	0	0	0	0	0	0	0	0	0
119	0	0	0	0	0	20	0	0	0	0	0	0	0	0	0	0	0	0	0	0
120	0	0	0	0	0	0	0	0	0	0	0	0	0	0	20	0	0	0	0	0
121	0	0	19	0	0	0	0	0	0	0	0	1	0	0	0	0	0	0	0	0
122	0	0	0	0	0	0	0	0	0	0	0	0	0	0	0	0	0	0	0	20
123	0	0	0	0	0	0	0	18	0	2	0	0	0	0	0	0	0	0	0	0
124	20	0	0	0	0	0	0	0	0	0	0	0	0	0	0	0	0	0	0	0
125	0	0	0	0	0	0	0	0	0	20	0	0	0	0	0	0	0	0	0	0
126	0	0	0	0	0	0	0	0	0	0	0	20	0	0	0	0	0	0	0	0
127	0	0	0	17	0	0	0	0	0	0	0	0	0	3	0	0	0	0	0	0
128	0	0	19	1	0	0	0	0	0	0	0	0	0	0	0	0	0	0	0	0
129	0	0	0	0	0	0	0	0	0	20	0	0	0	0	0	0	0	0	0	0
130	0	0	0	0	0	0	0	0	0	0	0	0	0	0	20	0	0	0	0	0
131	0	0	0	0	0	0	0	0	0	0	0	0	0	0	0	20	0	0	0	0
132	0	0	0	0	0	0	0	0	0	0	0	0	0	0	0	0	0	0	20	0
133	0	0	0	0	0	0	0	0	0	0	0	0	0	0	0	0	20	0	0	0
134	20	0	0	0	0	0	0	0	0	0	0	0	0	0	0	0	0	0	0	0
135	20	0	0	0	0	0	0	0	0	0	0	0	0	0	0	0	0	0	0	0
136	0	0	20	0	0	0	0	0	0	0	0	0	0	0	0	0	0	0	0	0
137	0	0	0	0	0	0	0	0	0	0	0	0	0	0	0	0	20	0	0	0
138	19	0	0	1	0	0	0	0	0	0	0	0	0	0	0	0	0	0	0	0
139	20	0	0	0	0	0	0	0	0	0	0	0	0	0	0	0	0	0	0	0
140	0	0	0	0	0	0	0	0	0	0	0	0	0	20	0	0	0	0	0	0

Position	A	C	D	E	F	G	H	I	K	L	M	N	P	Q	R	S	T	V	W	Y
141	0	0	0	0	0	0	0	20	0	0	0	0	0	0	0	0	0	0	0	0
142	0	0	0	0	0	0	0	0	0	0	0	0	0	0	0	0	20	0	0	0
143	0	0	0	0	0	0	0	0	20	0	0	0	0	0	0	0	0	0	0	0
144	0	0	0	0	0	0	0	0	0	0	0	0	0	0	20	0	0	0	0	0
145	0	0	0	0	0	0	0	0	20	0	0	0	0	0	0	0	0	0	0	0
146	0	0	0	0	0	0	0	0	0	0	0	0	0	0	2	0	0	0	18	0
147	0	0	0	20	0	0	0	0	0	0	0	0	0	0	0	0	0	0	0	0
148	20	0	0	0	0	0	0	0	0	0	0	0	0	0	0	0	0	0	0	0
149	16	0	0	4	0	0	0	0	0	0	0	0	0	0	0	0	0	0	0	0
150	0	0	6	0	0	14	0	0	0	0	0	0	0	0	0	0	0	0	0	0
151	6	0	1	3	0	0	0	0	0	0	0	5	0	0	0	0	0	1	0	4
152	20	0	0	0	0	0	0	0	0	0	0	0	0	0	0	0	0	0	0	0
153	0	0	0	20	0	0	0	0	0	0	0	0	0	0	0	0	0	0	0	0
154	0	0	0	0	0	3	0	0	0	0	0	0	0	0	7	7	2	1	0	0
155	0	0	0	5	2	1	1	0	0	3	0	0	0	5	0	0	0	1	2	0
156	0	0	0	0	0	0	0	0	0	0	0	0	0	0	20	0	0	0	0	0
157	0	0	0	0	0	0	0	0	0	0	0	20	0	0	0	0	0	0	0	0
158	0	0	0	0	0	0	0	0	0	0	0	0	0	0	0	0	0	0	0	20
159	0	0	0	0	0	0	0	0	0	18	0	0	0	0	0	0	0	2	0	0
160	0	0	0	20	0	0	0	0	0	0	0	0	0	0	0	0	0	0	0	0
161	0	0	0	0	0	20	0	0	0	0	0	0	0	0	0	0	0	0	0	0
162	0	0	0	6	0	0	0	0	0	2	0	0	0	0	8	0	4	0	0	0
163	0	20	0	0	0	0	0	0	0	0	0	0	0	0	0	0	0	0	0	0
164	0	0	0	0	0	0	0	0	0	0	0	0	0	0	0	0	0	20	0	0
165	0	0	0	20	0	0	0	0	0	0	0	0	0	0	0	0	0	0	0	0
166	0	0	0	0	0	10	0	0	0	0	0	0	0	0	0	0	0	0	10	0
167	0	0	0	0	0	0	0	0	0	20	0	0	0	0	0	0	0	0	0	0
168	0	0	0	0	0	0	0	0	0	0	0	0	0	0	20	0	0	0	0	0
169	0	0	0	0	0	0	0	0	0	0	0	0	0	0	20	0	0	0	0	0
170	0	0	0	0	0	0	1	0	0	0	0	0	0	0	0	0	0	0	0	19
171	0	0	0	0	0	0	0	0	0	20	0	0	0	0	0	0	0	0	0	0
172	0	0	0	20	0	0	0	0	0	0	0	0	0	0	0	0	0	0	0	0
173	0	0	0	0	0	0	0	0	0	0	0	19	0	0	0	0	1	0	0	0
174	0	0	0	0	0	20	0	0	0	0	0	0	0	0	0	0	0	0	0	0
175	0	0	0	0	0	0	0	0	20	0	0	0	0	0	0	0	0	0	0	0
176	0	0	20	0	0	0	0	0	0	0	0	0	0	0	0	0	0	0	0	0

Position	A	C	D	E	F	G	H	I	K	L	M	N	P	Q	R	S	T	V	W	Y
177	10	0	0	0	0	0	0	0	0	0	0	0	0	0	0	0	10	0	0	0
178	0	0	0	0	0	0	0	0	0	20	0	0	0	0	0	0	0	0	0	0
179	0	0	0	0	0	0	0	0	0	20	0	0	0	0	0	0	0	0	0	0
180	0	0	0	0	0	0	0	0	0	0	0	0	0	0	20	0	0	0	0	0
181	20	0	0	0	0	0	0	0	0	0	0	0	0	0	0	0	0	0	0	0
